# Single cell transcriptomic analysis of human pluripotent stem cell chondrogenesis

**DOI:** 10.1038/s41467-020-20598-y

**Published:** 2021-01-13

**Authors:** Chia-Lung Wu, Amanda Dicks, Nancy Steward, Ruhang Tang, Dakota B. Katz, Yun-Rak Choi, Farshid Guilak

**Affiliations:** 1 Dept. of Orthopaedic Surgery, Washington University in Saint Louis, St. Louis, MO 63110 USA; 2 Shriners Hospitals for Children—St. Louis, St. Louis, MO 63110 USA; 3 Dept. of Biomedical Engineering, Washington University in Saint Louis, St. Louis, MO 63110 USA; 4 Dept. of Orthopaedic Surgery, Yonsei University, Seoul, South Korea; 5 Present Address: Department of Orthopaedics and Rehabilitation, Center for Musculoskeletal Research, University of Rochester, Rochester, NY 14627 USA

**Keywords:** Gene regulatory networks, Cartilage development, Induced pluripotent stem cells, Osteoarthritis

## Abstract

The therapeutic application of human induced pluripotent stem cells (hiPSCs) for cartilage regeneration is largely hindered by the low yield of chondrocytes accompanied by unpredictable and heterogeneous off-target differentiation of cells during chondrogenesis. Here, we combine bulk RNA sequencing, single cell RNA sequencing, and bioinformatic analyses, including weighted gene co-expression analysis (WGCNA), to investigate the gene regulatory networks regulating hiPSC differentiation under chondrogenic conditions. We identify specific *WNT*s and *MITF* as hub genes governing the generation of off-target differentiation into neural cells and melanocytes during hiPSC chondrogenesis. With heterocellular signaling models, we further show that WNT signaling produced by off-target cells is responsible for inducing chondrocyte hypertrophy. By targeting WNTs and MITF, we eliminate these cell lineages, significantly enhancing the yield and homogeneity of hiPSC-derived chondrocytes. Collectively, our findings identify the trajectories and molecular mechanisms governing cell fate decision in hiPSC chondrogenesis, as well as dynamic transcriptome profiles orchestrating chondrocyte proliferation and differentiation.

## Abstract (web summary)

Application of human induced pluripotent stem cells (hiPSCs) for tissue regeneration is hindered by off-target cell differentiation. Here, the authors use bulk and single cell RNA-sequencing to identify WNT and MITF as off-target hubs during chondrogenic differentiation; inhibiting these pathways enhanced homogeneity and yield.

## Introduction

Osteoarthritis (OA) is a debilitating joint disease characterized by cartilage degeneration and pathologic remodeling of other joint tissues. Cartilage has limited intrinsic healing capacity, motivating the application of stem cells for regenerative therapies. In this regard, the advent of human induced pluripotent stem cells (hiPSCs) has served as a major breakthrough toward cartilage regenerative therapies and in vitro disease modeling for OA drug discovery^1^. However, the development of protocols to consistently differentiate hiPSCs into chondrocytes remains challenging. Early studies reported that chondrocytes can be generated from hiPSCs via embryoid body formation followed by monolayer expansion of mesodermal cells and three-dimensional cell pellet culture in chondrogenic induction medium^2,3^. Despite some success, this approach was proven difficult to reproduce across different iPSC lines, potentially due to variability in lots of fetal bovine serum (FBS) generally used for cell expansion. Thus, recent strategies have sought to use serum-free and chemically defined medium^4–6^. By coupling inductive and repressive signals required for mesoderm specification in embryonic development^7^, we established a step-wise hiPSC chondrogenic differentiation protocol that was validated with multiple hiPSC lines and in several laboratories^8^.

An important consideration in the differentiation process of hiPSCs is that they are considered to be in a primed pluripotent state with increased genome-wide DNA methylation compared to ground state naïve pluripotent cells, such as preimplantation blastocysts^9^. Therefore, even directed differentiation of hiPSCs can lead to the unpredictable formation of off-target cell populations. However, the gene regulatory networks (GRNs) leading to on- or off-target differentiation of hiPSCs, as well as the effect of the undesired cells on hiPSC chondrogenesis (i.e., heterocellular signaling), remain to be elucidated, particularly at the single-cell level.

Here, we apply bulk RNA sequencing (bulk RNA-seq) and single-cell RNA sequencing (scRNA-seq) throughout the process of mesodermal and chondrogenic differentiation of hiPSCs to map the dynamics of gene expression. By exploiting single-cell transcriptomics, we confirm the mesodermal and chondrogenic differentiation of hiPSCs in addition to identifying the GRNs and critical hub genes regulating the generation of heterogenous off-target cells. We demonstrate that the homogeneity of hiPSC chondrogenesis can be significantly improved by inhibiting the molecular targets WNTs and MITF. In summary, this study develops and validates an enhanced hiPSC chondrogenic differentiation protocol.

## Results

### Bulk RNA-seq indicates successful differentiation of hiPSCs

**Fig. 1:**
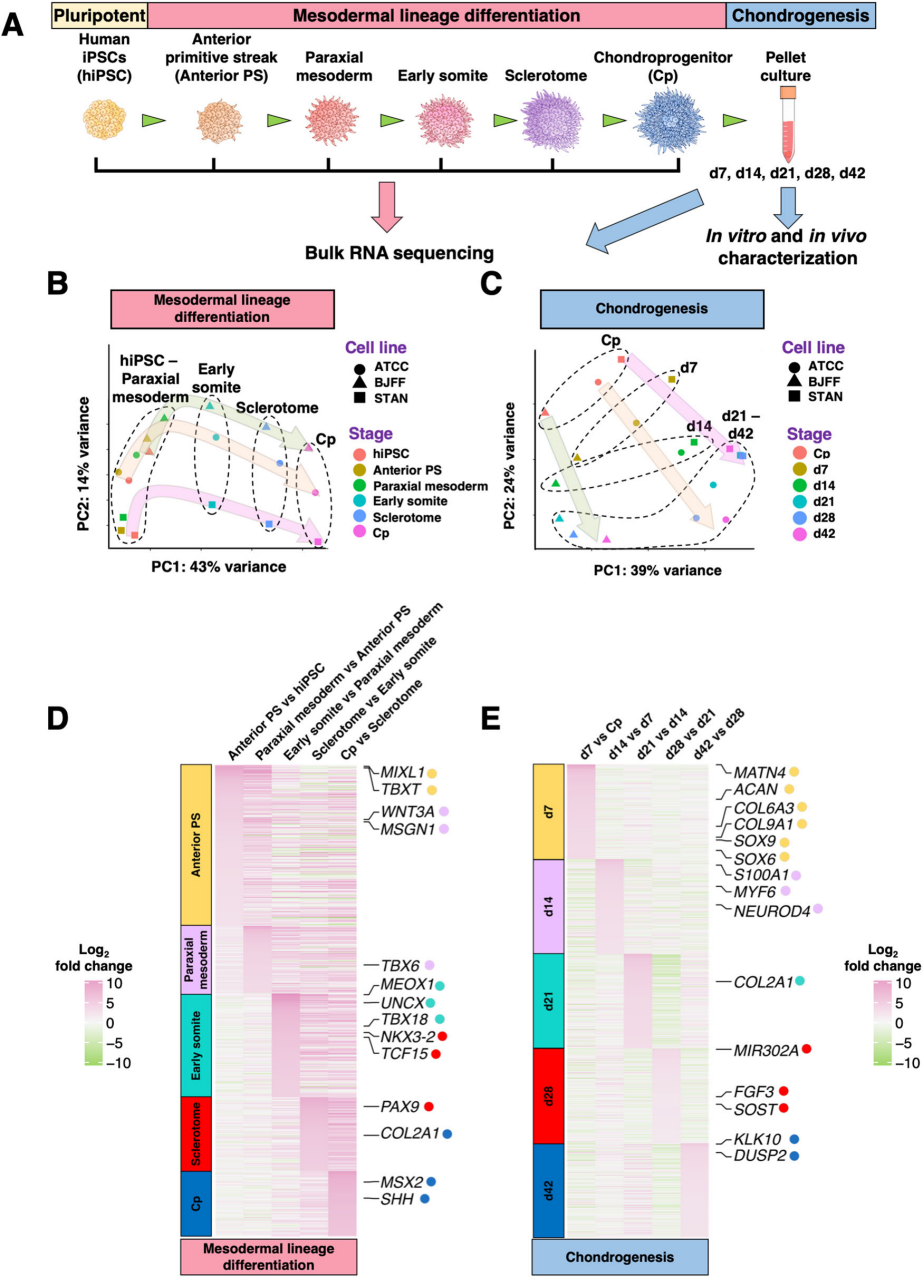
DEGs of mesodermal and chondrogenic differentiation of three hiPSC lines by bulk RNA-seq. **A** Schematic of the chondrogenic differentiation protocol for hiPSCs. **B**, **C** PCA indicates that three unique hiPSC lines followed similar differentiation trajectories. **D**, **E** DEGs averaged from three unique hiPSC lines at each stage of differentiation, respectively. Each column of the heatmap represents a comparison between two stages/time points, and each gene presented was assigned a colored dot (following the gene label). The color of the dot matches the color of the timepoint label on the left side of the heatmap. When the color of a gene label and a timepoint label match, that gene was significantly upregulated at the corresponding time points.

Previously, we reported a robust differentiation protocol that can drive hiPSCs toward a chondrogenic lineage via the paraxial mesoderm^7^ (Supplementary Fig. 1A, B). To determine transcriptome profiles over the course of differentiation, three independent hiPSCs lines (ATCC, BJFF, and STAN) were collected for bulk RNA-seq at various stages (Fig. 1A). Principal component analysis (PCA) reveals that the three hiPSC lines follow similar mesodermal and chondrogenic differentiation trajectories (Fig. 1B, C). Analysis of differentially expressed genes (DEGs) between each stage revealed upregulation of stage-specific markers. For example, T-box transcription factor T (*TBXT*) and mix paired-like homeobox (*MIXL1*) were upregulated at the anterior primitive streak (anterior PS) stage compared to hiPSCs^10^ (Fig. 1D; Supplementary Table 1). Markers representing mesodermal derivatives including T-box 6 (*TBX6*), UNC homeobox (*UNCX*), and paired box 9 (*PAX9*) were upregulated sequentially at the stages of paraxial mesoderm, early somite, and sclerotome, respectively (Fig. 1D; Supplementary Fig. 1C).

Chondrogenic markers such as matrilin 4 (*MATN4*), aggrecan (*ACAN*), collagen type VI alpha 3 chains (*COL6A3*), collagen type IX alpha 1 chain (*COL9A1*), and SRY-box 6 and 9 (*SOX6* and *SOX9*) were upregulated as early as at day 7 (d7), while the expression of collagen type II alpha 1 chain (*COL2A1*) was increased at d21 (Fig. 1E; Supplementary Table 2). Interestingly, microRNA-302a (*MIR302A*), reportedly downregulated in osteoarthritic chondrocytes, had enhanced expression in d28 pellets^11^. Neuronal differentiation 4 (*NEUROD4*), a gene encoding a transcriptional activator essential for neuronal differentiation, had increased expression in d14 pellets^12^.

### In vitro characterization of hiPSC-derived chondrocytes

**Fig. 2:**
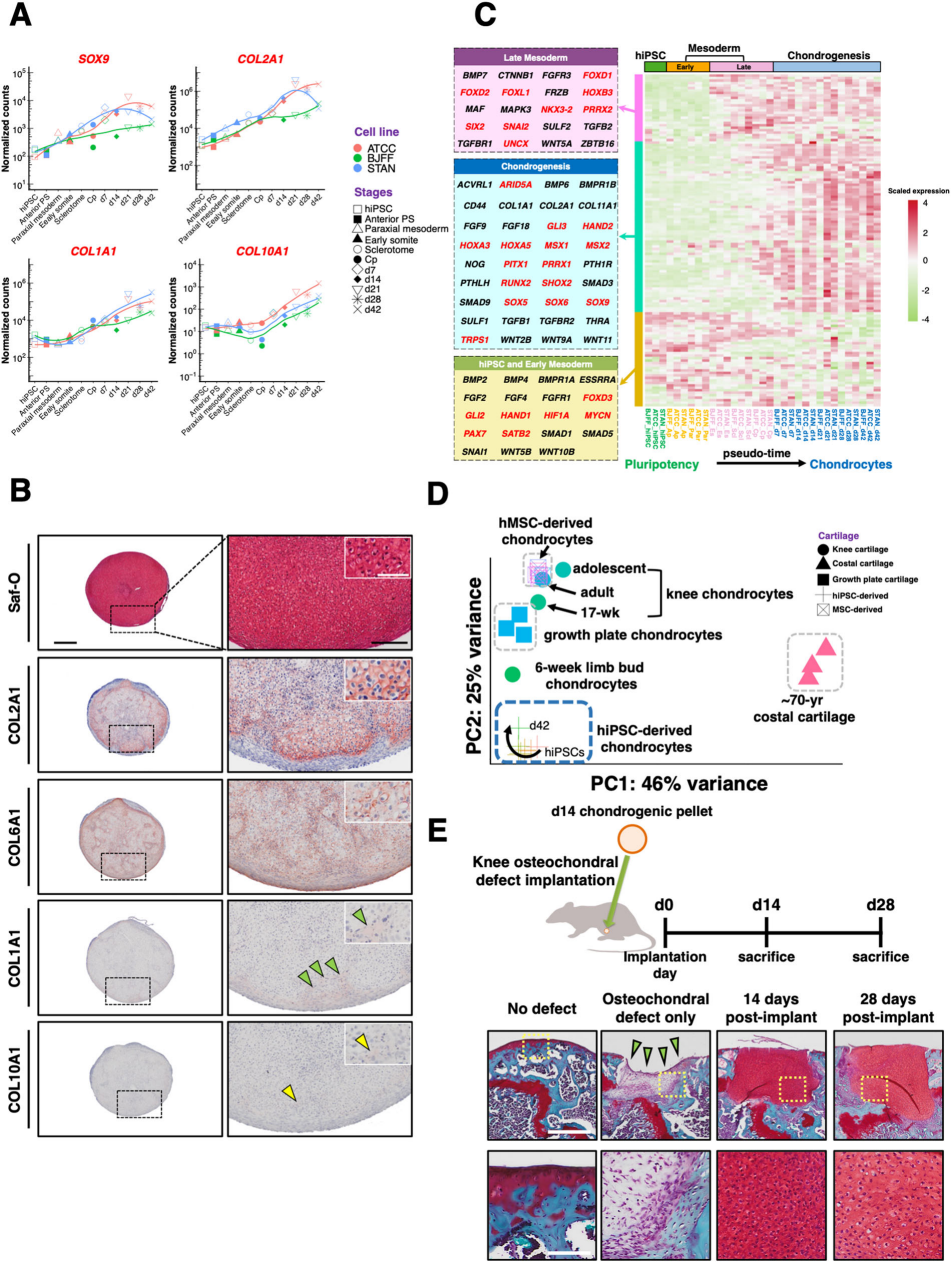
In vitro and in vivo characterization of hiPSC-derived chondrocytes. **A** Temporal gene expression of chondrogenic markers *SOX9* and *COL2A1*, hypertrophic marker *COL10A1*, and osteogenic marker *COL1A1*. **B** Pellets showed enriched Saf-O, COL2A1, and COL6A1 staining. Most COL1A1 staining (green arrowheads) was located at the edge of the pellets, while faint COL10A1 (yellow arrowheads) was observed. Left column scale bar = 400 µm. Right column scale bar = 200 µm. Inset scale bar = 50 µm. The experiment was repeated three times with similar results. **C** Heatmap of 134 significantly upregulated genes identified in GO term cartilage development (GO:0051216). Genes in red font are either TFs or transcription regulators. **D** hiPSC-derived chondrocytes exhibit a similar phenotype to embryonic limb bud chondrocytes. **E** hiPSC-derived chondrocytes repaired osteochondral defects in the cartilage of mouse knee joints and retained a chondrocyte phenotype 28 days post implantation. *n* = 3 mice per group. Top row scale bar = 500 µm. Bottom row scale bar = 100 µm.

While temporal expression of chondrogenic markers such as *SOX9* and *COL2A1* were upregulated in unique hiPSC lines, both the hypertrophic chondrocyte marker collagen type X alpha 1 chain (*COL10A1*) and osteogenic marker collagen type I alpha 1 chain (*COL1A1*) also exhibited increased expression over time (Fig. 2A). It is important to note that COL1A1 is also a marker for fibrous tissues, perichondrium, and many other cell types. The d28 pellet matrix also demonstrated rich proteoglycan staining using Safranin-O (Saf-O) as well as intense labeling for COL2A1 and COL6A1 by immunohistochemistry (IHC). However, little labeling for COL10A1 and COL1A1 was observed despite increased gene expression of *COL10A1* and *COL1A1* at later time points (Fig. 2B). Gene ontology (GO) enrichment analysis of the genes using R package GAGE was performed^13^. Significantly upregulated GO terms in Biological Process highlighted skeletal system and cartilage development (Supplementary Fig. 2A). GAGE analysis also revealed that 134 out of the 205 genes defined by cartilage development (GO:0051216) were significantly increased. Interestingly, in addition to upregulated *SOX5*, *6*, and *9*, which are known to be master transcription factors (TFs) governing chondrogenesis, we also observed several WNTs, including *WNT2B*, had increased gene expression at different stages during differentiation (Fig. 2C).

To determine the phenotype of hiPSC-derived cartilage, we projected our bulk RNA-seq data and publicly available sequencing datasets of primary chondrocytes from a variety of cartilaginous tissues and chondrocytes derived from human mesenchymal stem cells (hMSCs) in a PCA plot (Fig. 2D)^14^. We found that hiPSC-derived chondrocytes demonstrated a similar phenotype to embryonic limb bud chondrocytes.

### In vivo characterization of hiPSC-derived chondrocytes

To determine whether hiPSC-derived chondrocytes could maintain their phenotype in vivo, we implanted d14 pellets subcutaneously in the dorsal region of immunodeficient NSG (NOD.Cg-*Prkdc*^*scid*^ *Il2rg*^*tm1Wjl*^*/*SzJ) mice (Supplementary Fig. 2B). The d14 pellets represented the earliest time point when a chondrocyte-like phenotype was observed in vitro. After 14 days of implantation, pellets were harvested and found to retain a cartilage phenotype, with rich proteoglycan and COL2A1 labeling. No endochondral ossification was observed during this relatively short-term implantation period in our study.

To test whether hiPSC-derived chondrocytes can retain their phenotype within the joint, we created an osteochondral defect in the femoral groove of the mouse (Fig. 2E). Due to the small size of the mouse knee, the osteochondral defect model here also involves a growth plate defect. The defect was either left empty as a non-repair control group or filled with a d14 pellet. Defects left untreated did not exhibit any repair with hyaline cartilage, and only fibrotic tissue was observed. However, defects with pellet implantation demonstrated enhanced repair of the focal cartilage lesion, which was filled with cartilaginous matrix rich in Saf-O staining at both 14 and 28 days post implantation. While this finding provides proof-of-concept of the maintenance of the chondrogenic phenotype over 28 days, future studies may wish to investigate cell fate and implant properties after long-term implantation.

### scRNA-seq mapping of cellular heterogeneity

**Fig. 3:**
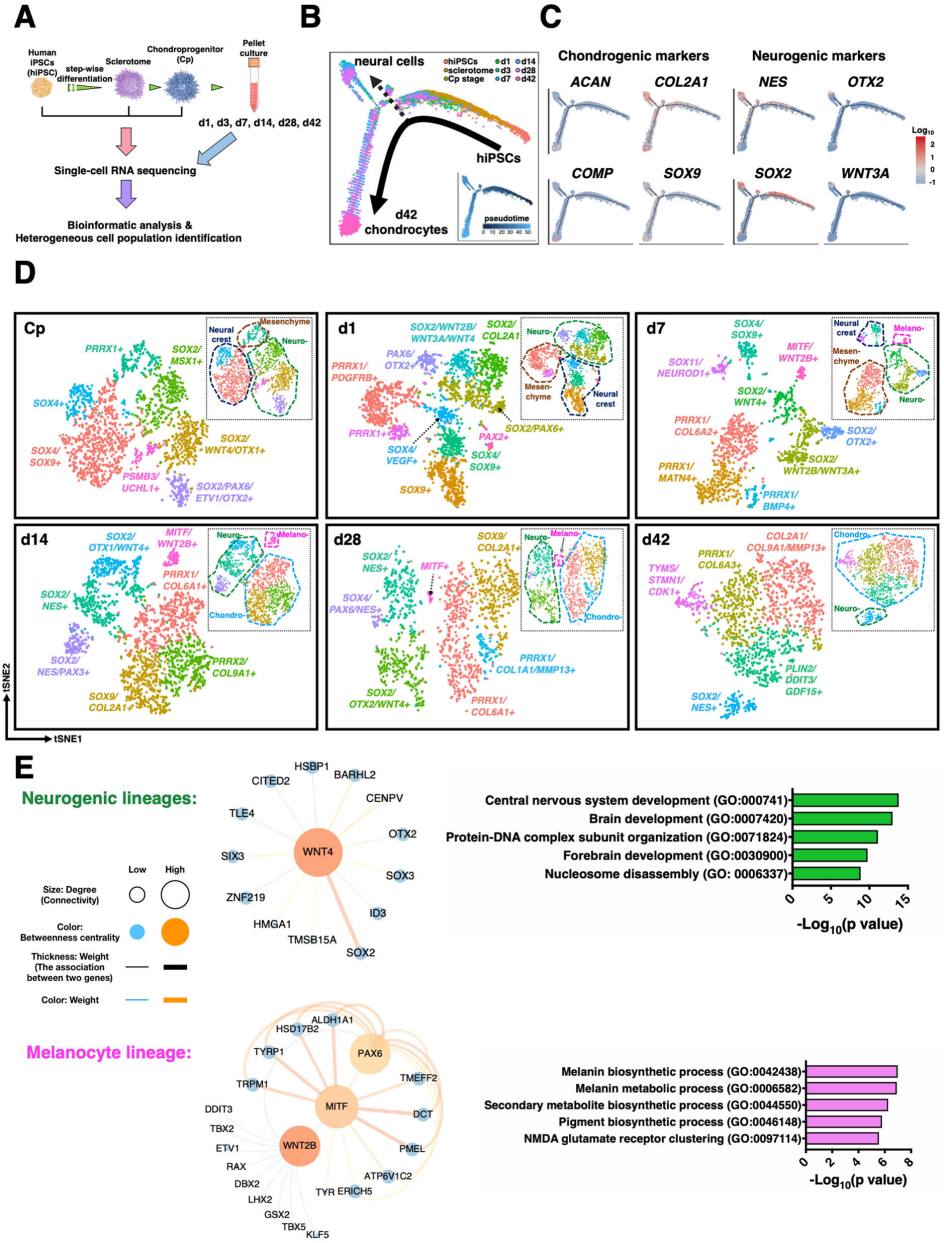
scRNA-seq and WGCNA reveal neural cells and melanocytes as off-target cells. **A** scRNA-seq was performed at hiPSC, Sclerotome, Cp, and six chondrogenic pellet time points. **B** Reconstruction of differentiation trajectory reveals an off-target lineage bifurcation toward neural cells. A total of 19,195 cells that passed quality control from the stage of hiPSC to d42 chondrogenic pellet were used to reconstruct the differentiation trajectory. **C** Chondrogenic markers were enriched in the chondrogenic branch, while neurogenic markers were observed in the branch of neurogenesis. **D** Annotated cell populations at different time points during hiPSC chondrogenesis. Cells that passed quality are used for tSNE plots; Cp: 1888 cells, d1: 2216 cells, d7: 1200 cells, d14: 2148 cells, d28: 1271 cells, and d42: 1328 cells. **E** WGCNA and GO term analysis identified *WNT4* as a hub gene of neurogenesis while *WNT2B* was highly associated with melanocyte development. scRNA-seq data of d14 pellets (with a total of 2148 cells and 3784 genes) was used for this computation.

Although our protocol generates a predominantly chondrocyte-like population as shown by IHC and bulk RNA-seq (Fig. 2B), we often observed non-chondrocyte populations and occasional focal accumulation of black-pigmented regions on the surface of the pellets (Supplementary Fig. 2C, D). These results suggest the presence of off-target differentiation, prompting us to seek their cellular identities. To dissect this cellular heterogeneity, nine samples from the STAN cell line at various differentiation time points were collected for scRNA-seq (Fig. 3A). Detailed cell numbers and median genes per cell for each stage are listed in Supplementary Table 5 (see “Methods” for quality control steps and criteria).

Sequencing of mixed-species ensured a low cell multiplet rate (2.7%) (Supplementary Fig. 3A). To verify the reproducibility of the differentiation, two batches of d28 samples were collected from independent experiments for scRNA-seq. Canonical correlation analysis (CCA) was used to align cells from the two batches^15^ (Supplementary Fig. 3B). The cells in the same cluster from different batches exhibited a high correlation in their gene expression (Spearman’s rank coefficient *r*_*s*_ > 0.87 for all clusters) (Supplementary Fig. 3C). Furthermore, genes that were highly conserved in one particular cluster showed similar expression patterns in the clusters from distinct batches, suggesting that our differentiation is highly reproducible (Supplementary Fig. 3D).

### Lineage bifurcation in hiPSC differentiation trajectory

We used the Monocle2 R package to reconstruct the differentiation trajectory from the stage of hiPSCs to d42 chondrocytes with a total of 19,195 cells that passed quality control (Fig. 3B)^16^. While cells following chondrogenic fate expressed chondrocyte markers, including *ACAN*, *COL2A1*, *SOX9*, and cartilage oligomeric matrix protein (*COMP*), we found one major branchpoint, diverting cell fate toward neural lineage with the expression of neural cell markers such as nestin (*NES*), orthodenticle homeobox 2 (*OTX2*), *SOX2*, and *WNT3A* (Fig. 3C). Other neural cell markers such as *OTX1* and *PAX6* were also enriched in this branch (Supplementary Fig. 3E). The off-target cell differentiation toward neurogenic lineage confirmed our findings of increased *NEUROD4* in the bulk RNA-seq data.

To explore distinct cell populations at each stage, scRNA-seq data were subjected to unsupervised clustering and visualized using t-distributed stochastic neighbor embedding (tSNE) plots (Fig. 3D). By comparing DEGs with signature genes of cell types in the literature and GO term analyses, we annotated broad cell populations by combining clusters expressing similar marker genes. For example, 2 of 7 clusters identified at the chondroprogenitor (Cp) stage not only had high expression levels of *SOX4* and *SOX9* but were also enriched in several markers resembling neural crest cells including *PAX3* and forkhead box D3 (*FOXD3*) (Supplementary Fig. 3F)^17^. Therefore, these two clusters were assigned to a broad cell population referred to as neural crest cells. Similarly, 4 clusters at the Cp stage exhibited markers of the neural lineage including *SOX2*, *OTX1/2*, and *PAX6*, and thus were annotated as neurogenic lineage cells, while *PRRX1*, *COL1A1*, and *COL3A1* are known markers for mesenchyme (Supplementary Fig. 3G)^18^. Similar major cell populations were also observed in d1 and d3 pellets, and it appeared that the percentage of chondrogenic cells increased in d7 while there was a decreased percentage of neural crest cells over time (Supplementary Fig. 3H, I).

Of note, a cluster with high expression of melanocyte-inducing TF (*MITF*) was observed in d7 and d14 pellets. MITF is a master TF regulating the development of melanocytes, cells that produce melanin (i.e., pigment)^19^. IHC of the pellets labeling for NES and MITF further confirmed the presence of neural cells and melanocytes (Supplementary Fig. 3J), suggesting that the focal black dots observed at the surface of pellets are likely to be the pigment accumulation in melanocytes. Furthermore, mesenchymal cells in d14 pellets expressed several conventionally recognized MSC markers (Supplementary Fig. 3K). Nevertheless, as distinct subtypes of hiPSC-derived chondrocytes and off-target cells were defined primarily based on marker genes, the complete functionality of these populations requires future investigation.

### WGCNA identifies GRNs of neurogenesis and melanogenesis

Next, we aimed to improve hiPSC chondrogenesis by decreasing off-target differentiation. We performed weighted gene co-expression network analysis (WGCNA) to reconstruct GRNs and identify the hub genes that modulate neurogenesis and melanogenesis^20^. scRNA-seq data of d14 pellets (with a total of 2148 cells and 3784 genes) were used for this computation due to the earliest presence of both chondrogenic and off-target populations detected. Five major gene modules (each containing >150 genes) were identified and based on GO enrichment analyses, they were categorized into cell division, cilium movement and assembly, skeletal system development, nervous system development, and melanin biosynthetic process. The genes in the modules of nervous system development and melanin biosynthetic process were then used to build corresponding GRNs and subnetworks by Cytoscape, while hub genes were determined by degree (node connectivity), weight (association between two genes), and betweenness centrality (BC) measure of the network (Fig. 3E and Supplementary Fig. 4A–C)^21^. In the GRN of neurogenesis, *WNT4* was strongly associated with several TFs regulating neural differentiation. We also observed that *WNT2B* was associated with both *MITF* and ETS variant 1 (*ETV1*), a gene whose activity has been reported to positively regulate *MITF*^22^.

### Inhibition of WNT signaling enhances hiPSC chondrogenesis

**Fig. 4:**
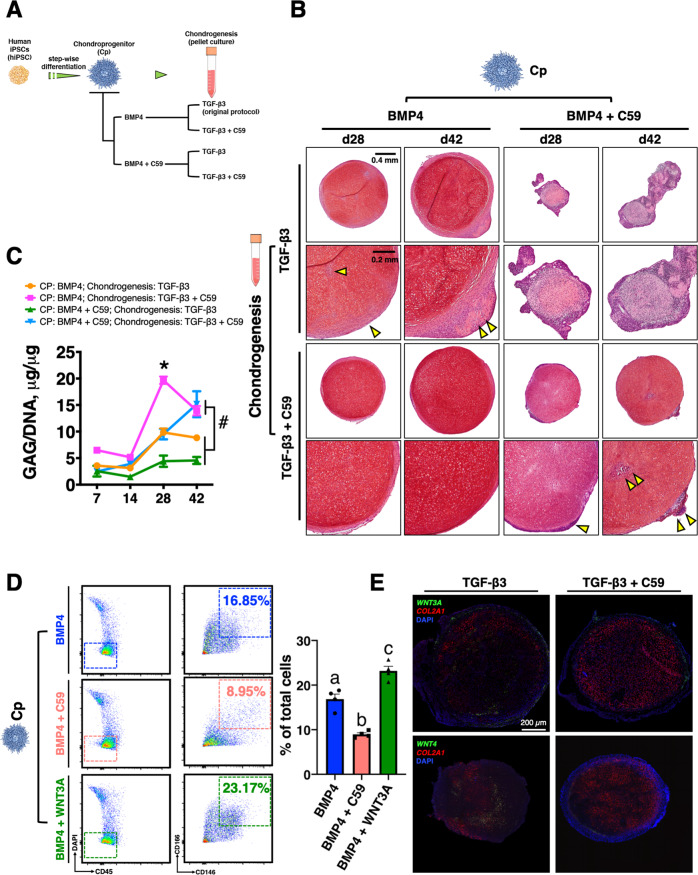
WNT inhibition during pellet culture enhanced homogeneity of hiPSC chondrogenesis. **A** Experimental scheme of WNT inhibition. **B** C59 treatment during pellet culture enhanced Saf-O staining and decreased off-target cells (yellow arrowheads) as compared to other WNT inhibition culture regiments. Top row scale bar = 400 µm. Bottom row scale bar = 200 µm. The experiment was performed twice with similar results. **C** Pellets treated with C59 in only pellet culture exhibited an increased GAG/DNA ratio compared to pellets treated with other culture regiments. *p = 0.00001 at d28. ^#^*p* = 0.0228 at d42. Mean ± SEM. *n* = 4 pellets per group. Statistical significance was determined by one-way ANOVA with Tukey’s post hoc test at a specific time point. **D** C59 significantly decreased, but WNT3A significantly increased, CD146^+^/CD166^+^/CD45^−^ progenitors at the Cp stage. Different letters are significantly different (a vs. b, *p* = 0.0005; a vs. c, *p* = 0.0021; b vs. c, *p* = 0.0001). Mean ± SEM. *n* = 3 per group (independent experiment). Statistical significance was determined by one-way ANOVA with Tukey’s post hoc test. **E** RNA-FISH of d28 pellets showing C59-treated pellets had decreased *WNT3A* and *WNT4* labeling (green) but more homogenous *COL2A1* distribution (red) in the pellets. Scale bar = 200 µm. The experiment was performed twice with similar results.

As WNTs were identified as essential genes in the off-target cells, we hypothesized that inhibition of WNT signaling may improve hiPSC chondrogenesis by decreasing undesired cell populations. It is known that WNTs are required to properly specify somites from pluripotent cells^23^. Therefore, we administrated Wnt-C59 (C59), a WNT inhibitor, at either the Cp stage and/or during the chondrogenic pellet culture (i.e., four different inhibition regimens, Fig. 4A). Chondrocyte homogeneity, as indicated by Saf-O staining, was increased if WNT signaling was inhibited during pellet culture (Fig. 4B). This finding was reflected by the increased production of glycosaminoglycans per cell (GAG/DNA ratio) in the group receiving C59 during the pellet culture (Fig. 4C). However, inhibiting WNTs at the Cp stage severely impaired chondrogenesis. Mesenchymal cells that are positive for CD146 and CD166 are proposed to be putative Cps due to their robust chondrogenic potential^24^. Flow cytometric analysis showed that C59 treatment largely decreased the percentage of CD146/CD166^+^ cells, while WNT3A supplementation increased this population at the Cp stage (Fig. 4D). Similar results were observed using two additional hiPSC lines (ATCC and BJFF) (Supplementary Fig. 4D–G). Interestingly, pellets derived from hMSCs with WNT inhibition also exhibited increased Saf-O staining (Supplementary Fig. 4H, I). In addition, hiPSC pellets receiving combined administration of C59 and ML329 (ML), an MITF antagonist, also exhibited enhanced chondrocyte homogeneity compared to standard TGF-β3 treatment (Supplementary Fig. 4G).

RNA fluorescence in situ hybridization (RNA-FISH) labeling of WNTs and *COL2A1* within d28 pellets indicated that although some labeling could be detected in the center of the pellets, most WNTs were located in the perichondral layer, consistent to the inhomogeneous cell populations observed via IHC staining. Furthermore, C59-treated pellets showed a more homogenous distribution of *COL2A1* RNA-FISH labeling vs. TGF-β3-treated pellets (Fig. 4E and Supplementary Fig. 5).

### scRNA-seq confirms WNT inhibition enhances chondrogenesis

**Fig. 5:**
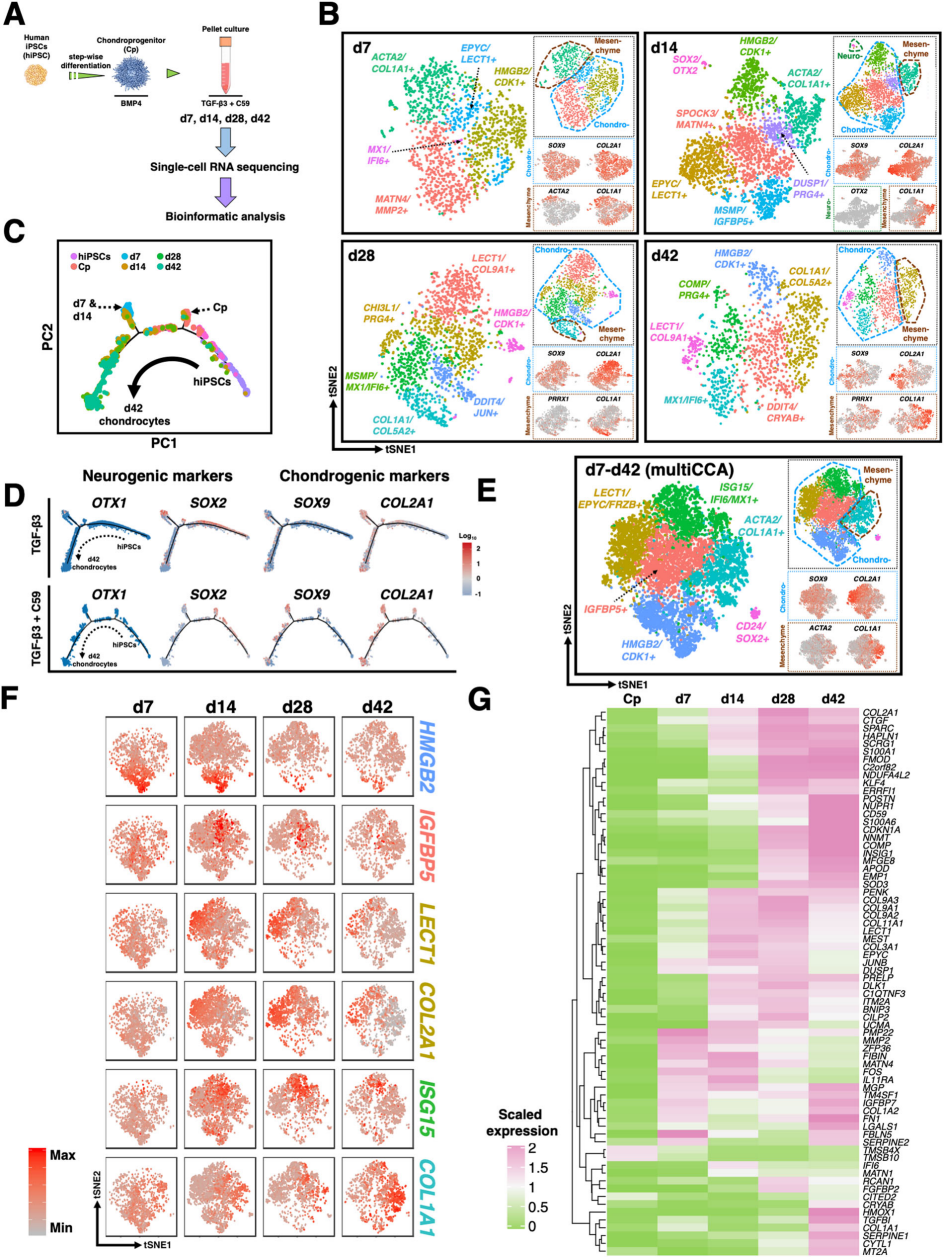
scRNA-seq of pellets with WNT inhibition shows improved chondrogenesis. **A** scRNA-seq was performed on the pellets with WNT inhibition. **B** Chondrocytes and mesenchymal cells were two major populations in C59-treated pellets. Cells that passed quality control were used for tSNE plots; hiPSC: 4798 cells, Cp: 1888 cells, d7: 1682 cells, d14: 3076 cells, d28: 1756 cells, and d42: 1483 cells. **C** Differentiation trajectory of C59-treated pellets. scRNA-seq data with a total of 14,683 cells from the stage of hiPSC, Cp as well as d7, d14, d28, and d42 C59-treated pellets were used to reconstruct the differentiation trajectory. **D** C59-treated pellets exhibited decreased neurogenic markers but increased chondrogenic markers. **E** Multiple CCA alignment of d7–d42 pellets. A total of 7977 cells from d7–d42 timepoints of C59-treated pellets were used to perform CCA alignment. **F** Dynamic changes in gene expression and percentages of chondrocyte subpopulations over time. **G** Heat map of top 20 DEGs at each timepoint for *LECT1/EPYC/FRZB*^+^ early mature chondrocytes.

To determine how WNT inhibition altered cell populations in chondrogenesis and to identify chondrocyte subpopulations, pellets treated with C59 were analyzed using scRNA-seq with a total of 14,683 cells from the stage of hiPSC, Cp as well as d7, d14, d28, and d42 C59-treated pellets (Fig. 5A, B). We found the C59-treated pellets comprised two major cell populations: mesenchyme and chondrocytes. Mesenchyme exhibited high expression of actin (*ACTA2*), *PRRX1*, *COL1A1*, and *COL3A1*. Most importantly, neural cells and melanocytes were significantly decreased with WNT inhibition. The differentiation trajectory of C59-treated chondrogenesis was reconstructed, using scRNA-seq datasets of hiPSC and Cp stages from the previous sequencing (since they did not involve C59 intervention) (Fig. 5C). Compared to the trajectory built from TGF-β3-treated pellets, C59-treated pellets exhibited little, if any, neurogenic markers, but showed enriched expression for chondrogenic markers (Fig. 5D). In pseudotime analysis, we found that C59-treatment led to earlier induction of *ACAN* expression, higher levels of *COL2A1* and *SOX9* expression, and an earlier decrease in *SOX2* expression as compared to pellets treated with TGF-β3 alone (Supplementary Fig. 6A).

Chondrocytes in C59-treated pellets comprised several subpopulations as identified by multiple CCA alignment of d7-d42 timepoints with a total of 7997 cells (Fig. 5E, F, and Supplementary Fig. 6B, C), including one mesenchymal population and four conserved chondrocyte subsets with enriched *COL2A1* and *SOX9* expression. The chondrocyte subset enriched in cell cycling markers, such as high mobility group box 2 and cyclin-dependent kinase 1 (*HMGB2/CDK1*^+^), was defined as proliferating chondrocytes^25^. The second chondrocyte subset was enriched in IGF-binding protein-5 (*IGFBP5*). It has been previously reported that IGFBP5 is highly upregulated in the early differentiating stage^26^. Hence, the *IGFBP5*^+^ chondrocyte subset was defined as a population of early differentiating chondrocytes. The third chondrocyte subset expressed leukocyte cell-derived chemotaxin 1, epiphycan, and frizzled-related protein (*LECT1/EPYC/FRZB*^+^) and had the highest levels of *COL2A1* and *ACAN* expression among other chondrocyte subsets. Therefore, the *LECT1/EPYC/FRZB*^+^ chondrocyte subset was defined as a population of early mature chondrocytes. Finally, we identified a unique chondrocyte subset expressing interferon (IFN)-related genes including ISG15 ubiquitin-like modifier, interferon-alpha inducible protein 6, and MX dynamin-like GTPase 1 (*ISG15/IFI6/MX1*^+^). We observed that 4.6% of *ISG15/IFI6/MX1*^+^ chondrocytes co-expressed terminal hypertrophic differentiation markers *VEGFA* and *MMP13*; thus, we defined the *ISG15/IFI6/MX1*^+^ chondrocyte subset as mature-hypertrophic chondrocytes (Supplementary Fig. 6D).

At early timepoint d7, *HMGB2/CDK1*^+^ proliferating chondrocytes was the main cell population (44.5%) within the pellets (Supplementary Fig. 6C). Interestingly, this population also had the highest numbers of *BMPR1B/ITGA4* double-positive cells, a rare osteochondral progenitor population found in articular cartilage (Supplementary Fig. 6E, F)^14^. When proliferating chondrocytes differentiated toward maturity, potentially facilitated by IGFBP5^26^, *IGFBP5*^+^ early differentiating chondrocytes and *LECT1/EPYC/FRZB*^+^ early mature chondrocytes became dominant (Supplementary Fig. 6C). The enriched expression of *FRZB*, which encodes a secretory WNT inhibitor, in early mature chondrocytes might help stabilize this population by further antagonizing WNT signaling in addition to C59 treatment (Supplementary Fig. 6G). As *LECT1/EPYC/FRZB*^+^ chondrocytes had the highest levels of *COL2A1* and *ACAN* expression, we investigated the DEGs of this particular population at various time points (Fig. 5G). Among several early chondrogenic markers and osteogenic markers, *COL1A2* and *IGFBP7* exhibited biphasic upregulation at both early and later time points of chondrogenesis.

The percentage of *ISG15/IFI6/MX1*^+^ mature-hypertrophic chondrocytes greatly increased at d28 (Supplementary Fig. 6C). Although the downstream IFN regulatory molecules including *STAT1* and *PML* were elevated in this population, we could not detect any type of IFNs which were conventionally believed to be the activators of IFN pathways (Supplementary Fig. 6H). Instead, we observed that *IGFBP3* was enriched in *ISG15/IFI6/MX1*^+^ chondrocytes, whereas *IGFBP5* was highly expressed in early differentiating chondrocytes. In line with the results of previous studies, we also observed that IGFBP3 inhibited expression of *FOS* (*C-FOS*), a possible driver of chondrocyte hypertrophy when it dimerizes with *JUN* (*AP-1*) (Supplementary Fig. 6I)^27^. This result may provide some explanations for the finding that *ISG15/IFI6/MX1*^+^ chondrocytes had variable expression levels of hypertrophic chondrocyte markers (Supplementary Fig. 6J)^28^.

During chondrogenic culture, pellets were generally surrounded by a fibrous layer, resembling the cartilage anlage enclosed by fibroblastic cells (i.e., perichondrium). To determine if the mesenchyme (i.e., *ACTA2/PRRX1/COL1A1*^+^ cells) identified in pellets and the mesenchyme (i.e., *PRRX1*^+^ cells) identified at the Cp stage (monolayer culture) were similar to the perichondrium, we benchmarked these mesenchymal cells, as well as various chondrocyte subpopulations, against previously reported markers of perichondrial cells in rats and humans (Supplementary Fig. 7)^29,30^. We found that *ACTA2/PRRX1/COL1A1*^+^ cells in pellets, but not *PRRX1*^+^ cells at the Cp stage, were enriched in genes of perichondrium, suggesting that the mesenchymal population at the Cp stage and the mesenchymal population in pellets had distinct phenotypes, despite their shared mesenchymal genes such as *COL1A1* and *COL3A1*. The scRNA-seq data of C59-treated pellets were then used to reconstruct the GRN of hiPSC chondrogenesis with minimal presence of off-target cells as shown by WGCNA (Supplementary Fig. 8A).

### Differential gene expression profiles after C59 treatment

**Fig. 6:**
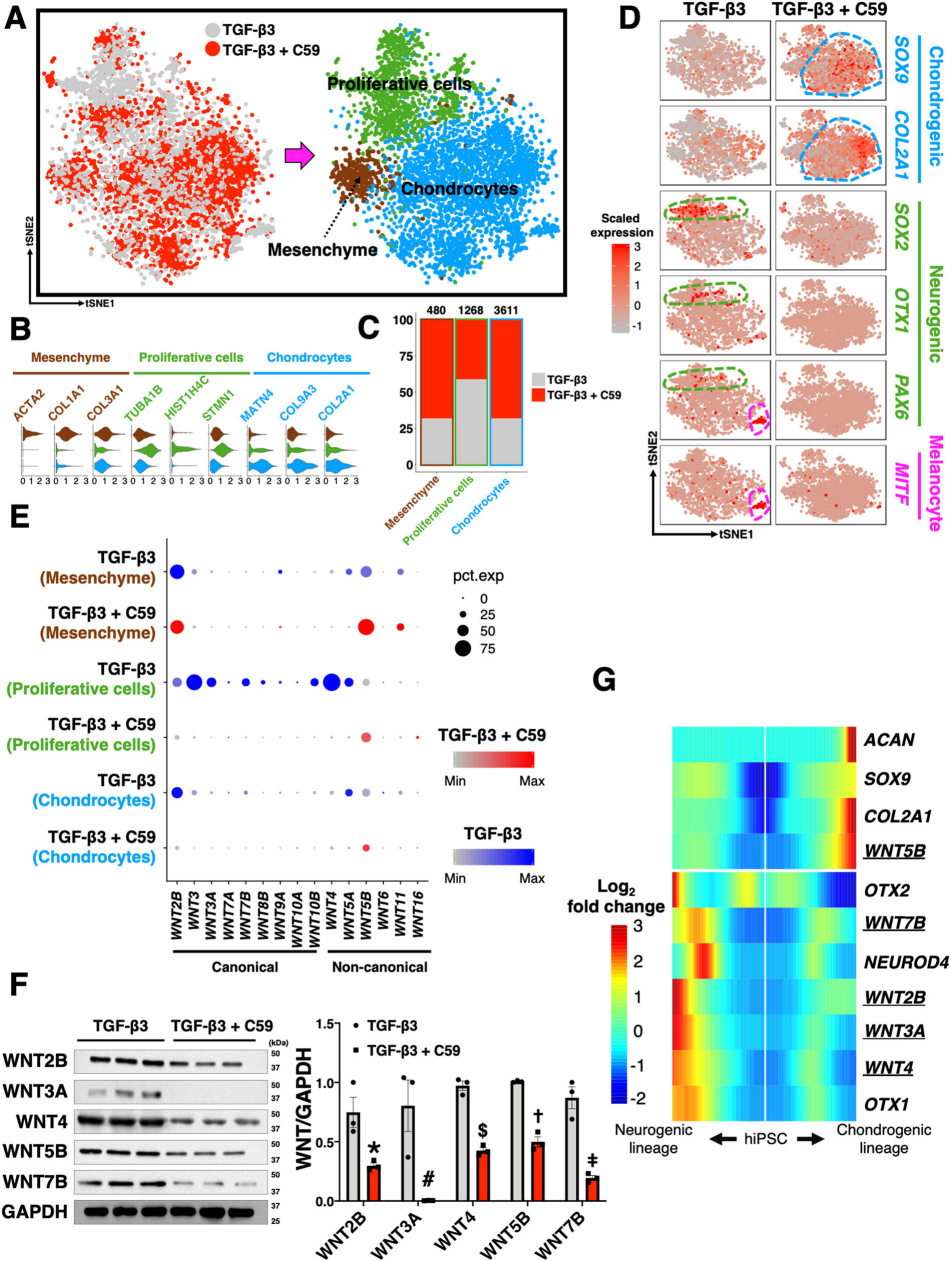
CCA analysis reveals that most WNTs, except WNT5B, were secreted by off-target cells. **A** Three major conserved populations in d14 pellets. A total of 5224 cells from the d14 pellets with or without C59 treatment was analyzed. **B** Violin plots of the specific markers for each conserved population. **C** C59-treated pellets comprised more chondrocytes and mesenchymal cells. **D** Expression levels of chondrogenic markers were higher in C59-treated pellets while expression of neurogenic markers and melanocyte markers was higher in TGF-β3-treated pellets. **E** Dot plot showing proliferative cells (mainly neural cells) from TGF-β3-treated pellets had high expression levels of WNT ligands. WNT inhibition largely decreased expression levels of WNTs in cells. **F** Western blots confirm that WNT inhibition significantly decreased WNTs in cells at protein levels. ^*^p = 0.026, ^#^*p* = 0.021, ^$^*p* = 0.0003, ^†^*p* = 0.00029, ^‡^*p* = 0.021 to its corresponding group. Mean ± SEM. *n* = 3 per treatment condition. Statistical significance was determined by a two-tailed Student’s *t* test for the groups with or without specific WNT inhibition. **G** Most WNTs were upregulated along the lineage of neural cells, where WNT5B was clustered with chondrogenic differentiation in TGF-β3-treated pellets. A total of 2148 cells from the TGF-β3-treated d14 pellets was analyzed and used to generate the heatmap.

Three major conserved populations were identified after CCA alignment of the d14 cells with or without C59 treatment (a total of 5224 cells analyzed): proliferative cells, mesenchyme enriched, and chondrocytes (Fig. 6A, B). C59-treated pellets contained more mesenchyme and chondrocytes at d14, while non-C59-treated (i.e., TGF-β3 only) pellets had more proliferative cells at the same time point (Fig. 6C). Pellets with only TGF-β3 treatment not only showed elevated expression of *MITF* but also had more neural cells which were clustered in proliferative cells (Fig. 6D). Chondrocytes and proliferative cells exhibited similar profiles of upregulated and downregulated DEGs. For instance, both cell populations showed upregulated expression of *COL2A1* and *JUNB*, while exhibiting decreased expression of *SOX4* and several ribosomal genes (Supplementary Fig. 8B). Interestingly, *FRZB* was only upregulated in the chondrocyte population upon C59 treatment.

At d28, pellets treated with C59 exhibited increased expression of *ACAN* and *COMP* compared to the standard-treated pellets (Supplementary Fig. 8C, D). Importantly, we also observed that *IFI6* and *ISG15*, markers for mature-hypertrophic chondrocytes, were downregulated in the C59-treated pellets, suggesting WNT inhibition may decrease chondrocyte hypertrophy during chondrogenesis.

### WNT expression with neurogenesis

To determine the expression patterns of WNTs and to identify the cells responsible for WNT production, we investigated WNT expression levels in multiple cell populations of d14 and d28 pellets (Fig. 6E and Supplementary Fig. 8E; a total of 5224 d14 cells and a total of 3027 d28 cells analyzed, respectively). In TGF-β3-treated pellets, several canonical WNTs, such as *WNT3*, *WNT3A*, and *WNT7B*, as well as noncanonical WNTs, including *WNT4*, were enriched in the proliferative population (where the neural cells clustered), while *WNT2B* and *WNT5B* could be found in proliferative cells, chondrocytes, and mesenchyme. We did not detect *WNT1*, *WNT2*, or *WNT8* in any specimens. Upon C59 treatment, most WNTs showed decreased expression, particularly in proliferative cells. Western blots confirmed that C59-treated pellets had decreased protein levels of WNT2B, WNT3A, WNT4, and WNT7B (Fig. 6F). Interestingly, C59 only moderately inhibited WNT5B. We next plotted these WNT ligands along with neurogenic and chondrogenic markers in pseudotime to investigate their expression patterns. We observed that *WNT2B*, *WNT3A*, *WNT4*, and *WNT7B* clustered with neurogenic markers, whereas *WNT5B* was upregulated along with chondrogenic differentiation, implying that individual WNTs may play distinct roles in regulating chondrogenesis (Fig. 6G).

### WNTs alter GAG/DNA and collagen production

**Fig. 7:**
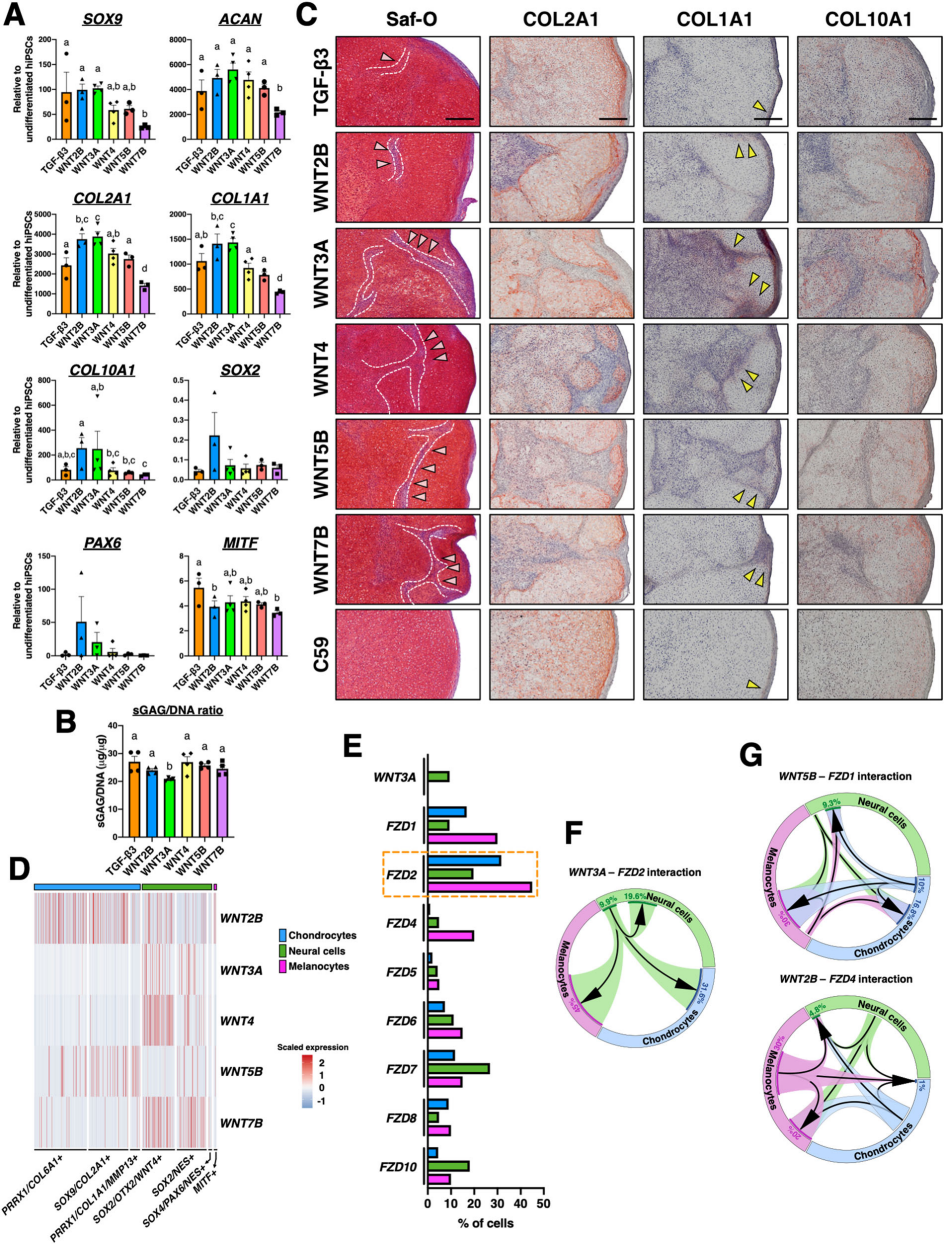
Heterogenous multicellular WNT signaling models. **A**, **B** RT-qPCR and GAG/DNA ratios of pellets treated with various WNTs during pellet culture. Different letters are significantly different from each other (*p* < 0.05). Mean ± SEM. *n* = 3–4 pellets per group. Statistical significance was determined by one-way ANOVA with Tukey’s post hoc test. **C** WNT treatment increased infiltration of off-target cells (pink arrowheads and white dashed lines) into the pellets, decreased COL2A1 staining, but increased COL1A1 (yellow arrowheads) and COL10A1 staining in the pellets. The pellets with C59 treatment exhibited homogenous COL2A1 staining and decreased COL1A1 and COL10A1 staining. Scale bar = 0.2 mm. The experiment was performed twice with similar results. **D** Heatmap showing distinct expression levels of various WNTs in various cellular subpopulations in d14 TGF-β3-treated pellets. A total of 2148 cells from the TGF-β3-treated d14 pellets was analyzed and used to generate the heatmap. **E** Percentage of the cells expressing *WNT3A* and its putative receptors in d14 TGF-β3-treated pellets. **F**, **G** Heterogenous multicellular signaling models in d14 TGF-β3-treated pellets.

As C59 is a pan-WNT signaling inhibitor, it, therefore, remained unknown which WNT ligand had the most severe adverse effect on hiPSC chondrogenesis. To answer this question, we administrated a variety of WNTs during pellet culture (Supplementary Fig. 9A). RT-qPCR analysis showed that only WNT7B significantly decreased chondrogenic markers (*SOX9*, *ACAN*, and *COL2A1*) and osteogenic marker (*COL1A1*) when compared to TGF-β3 only pellets (Fig. 7A). Interestingly, the pellets treated with WNT2B and WNT3A exhibited increased *COL2A1*, *COL1A1*, and *COL10A1* expression versus TGF-β3 pellets. However, only the pellets with WNT3A treatment had a significantly decreased GAG/DNA ratio compared to the pellets with TGF-β3 only treatment (Fig. 7B). WNT2B-treated pellets also showed a trend toward the increasing expression of neurogenic markers (*PAX6* and *SOX2*), although not statistically significant. Furthermore, WNT2B- and WNT7B-treated pellets had significantly lower expression of *MITF* relative to TGF-β3 pellets. We also observed that WNT ligands may not only regulate their own expression but may also modulate the expression of other WNT ligands (Supplementary Fig. 9B).

While all pellets had comparable Saf-O staining, WNT treatment increased off-target cells within the pellets (Fig. 7C). Furthermore, these off-target cells exhibited lower production of COL2A1 compared to chondrocytes. Additionally, pellets treated with WNTs, particularly WNT3A, exhibited higher intensity of COL1A1 and COL10A1 staining, which was observed near off-target cells and perichondrium. On the contrary, C59-treated pellets had low COL1A1 and COL10A1 production, and the staining was mainly at the perichondrium. Together, these results indicate that WNTs increased non-chondrogenic cells and modulated collagen production. The histological images in Fig. 7C were quantified using a published ImageJ protocol (Supplementary Fig. 9C)^31^.

### Heterocellular WNT signaling may regulate chondrogenesis

To investigate which cell populations are the main sources for the endogenous production of specific WNTs during chondrogenesis, a heatmap in which the expression of WNT ligands against multiple cell populations at the d14 timepoint was plotted (Fig. 7D). We found that 30% of melanocytes expressed *WNT2B*, while *WNT3A*, *WNT4*, and *WNT7B* were mainly expressed in neural cells (Supplementary Fig. 9D). *WNT5B* was expressed primarily by chondrocytes (about 10% of the chondrocyte population) providing a possible explanation for the upregulation of *WNT5B* during chondrogenesis. As WNTs are secretory proteins, we next aimed to identify the potential cell populations receiving WNT signaling based on published lists of ligand–receptor pairs^32^. We found that 31.6% of chondrocytes expressed *FZD2*, the highest expression of a WNT receptor in chondrocytes (Fig. 7E). Thus, we created the multicellular signaling for the *WNT3A-FZD2* pair and identified that 9.9% of neural cells expressed *WNT3A* while more than a third of chondrocytes (36.1%) were capable of receiving this ligand (Fig. 7F). In addition, we also observed that although chondrocytes were the major contributor to WNT5B production, melanocytes (30%) might be the main receiving cell type. Furthermore, while 30% of melanocytes may secrete WNT2B, only 1% of chondrocytes expressed *FZD4*, one of the main WNT2B receptors (Fig. 7G).

### BMP/GDF differential expression after C59 treatment

While the precise mechanisms of enhanced chondrogenesis remain to be determined, our CCA analysis showed that six chondrocyte subpopulations and one mesenchymal population were conserved between TGF-β3-treated and C59-treated d14 pellets: (1) *HMGB2/CDK1*^+^ proliferating chondrocytes, (2) *UBE2C/CCNB1*^+^ proliferating chondrocytes, (3) *LECT1/EPYC/FRZB*^+^ early mature chondrocytes, (4) *ISG15/IFI6/MX1*^+^ mature-hypertrophic chondrocytes, (5) *FTL/MT-CO2*^+^ stressed chondrocytes, (6) *BNIP3/FAM162A*^+^ apoptotic chondrocytes, and *ACTA2/PRRX1/COL1A1*^+^ mesenchymal cells (Supplementary Fig. 10A; CCA was performed with a total of 1335 cells from mesenchymal and chondrocyte populations from d14 TGF-β3 pellets and with a total of 3047 cells from mesenchymal and chondrocyte populations from d14 C59 pellets. It is important to note that off-target cells (i.e., neural cells and melanocytes) were exculded from this analysis). Interestingly, C59 treatment differentially influenced the expression of various growth factors and receptors in the TGF-β superfamily essential in regulating chondrogenesis^33^ (Supplementary Note 1, Supplementary Fig. 10B–E, Supplementary Fig. 11A, B, and Supplementary Fig. 12A, B).

## Discussion

The therapeutic applications of hiPSCs for cartilage regeneration or disease modeling have been limited by the low-yield of *bona fide* chondrocytes, accompanied by off-target populations during chondrogenic differentiation. Our GRN analysis revealed two major off-target cell populations, neural cells and melanocytes, which showed high association with WNT4 and WNT2B signaling, respectively. By building heterocellular signaling models, we showed that off-target cells were the main source of several canonical and noncanonical WNT ligands that were implicated in chondrocyte hypertrophic differentiation. Importantly, inhibition of WNT and MITF, the master regulator of melanocyte development, significantly enhanced homogeneity of hiPSC chondrogenesis by decreasing off-target cells, circumventing the need for prospective sorting and expansion of isolated progenitor cells.

An important finding of this study was the identification of distinct subtypes of hiPSC-derived chondrocytes, as shown in depth by the comprehensive transcriptomic profiles of each cell type at various differentiation stages. We also observed that inhibition of WNT signaling during chondrogenesis alters gene expression levels of BMPs/GDFs (e.g., decreasing *BMP4* and *BMP7* levels) in chondrocytes, which is consistent with a recent study demonstrating decreased BMP activity during MSC chondrogenesis due to WNT inhibition^34^. Another intriguing finding is the discovery of *ISG15/IFI6/MX1*^+^ mature-hypertrophic chondrocytes as, without scRNA-seq, this unique population has not been reported before. Although the signature genes of this chondrocyte population (e.g., *STAT-1*) were generally believed to be downstream of IFN-related pathways, we did not detect IFN expression. The high expression of *IGFBP3* in *ISG15/IFI6/MX1*^+^ chondrocytes may provide an explanation for this observation, as IGFBP3 can activate *STAT-1* expression without the presence of IFN molecules in chondrogenesis^35^. In addition, *IGFBP3*-enriched chondrocytes also had decreased expression of *FOS*, essential in driving chondrocytes toward hypertrophy^27^. It has been reported that chondrocyte hypertrophy was largely prevented upon *IGFBP3* knockdown in the ATDC5 line^36^. Thus, low FOS expression in *ISG15/IFI6/MX1*^+^ chondrocytes provides a plausible explanation for their low expression of hypertrophic markers. Nevertheless, the causal relationship between the dual function of *IGFBP3* in chondrocyte hypertrophy and WNT inhibition merits further study.

The finding that melanocytes and neural cells were the major off-target cells implies that some, if not all, progenitors may acquire the phenotype of neural crest cells, a transient stem cell population that can give rise to neurons and melanocytes. This differentiation pathway likely occurs at the Cp stage, where we first observed cell populations expressing several markers of neural crest cells. It is likely that the neural crest cells observed in the current study were also off-target cells (i.e., non-paraxial mesodermal lineage) generated during the early stages of mesodermal differentiation and amplified due to BMP4 treatment at the Cp stage. It has been reported that the *Bmp4-Msx1* signaling axis inhibits Wnt antagonists such as Dkk2 and Sfrp2 in dental mesenchyme in mice^37^, implying that BMP4 treatment may  promote WNT signaling that is essential for the proliferation of neural crest cells.

Additionally, our sorting results showed that supplementation of WNT increased, but inhibition of WNT decreased, the proportion of CD146/CD166^+^ cells, suggesting that WNT signaling is required to maintain progenitors at the Cp stage. This finding is in agreement with a recent study showing that WNT3A supports the multipotency of hMSCs during in vitro expansion^38^. In our recent publication using a CRISPR–Cas9-edited reporter hiPSC line and scRNA-seq techniques, we identified that mesenchymal cells triple-positive for CD146, CD166, and PDGFRβ, but negative for CD45, at the Cp stage showed robust chondrogenic potential but little osteogenic capacity compared to unsorted cells, suggesting that CD146/CD166/PDGFRβ^+^ mesenchymal cells may be a unique Cp population^39^. However, whether the CD146/CD166^+^ progenitor population identified in the current study functions like MSCs with multilineage potential warrants future investigation. Furthermore, as distinct subtypes of hiPSC-derived chondrocytes were defined primarily based on marker genes, the complete functionality of these subsets requires future investigation.

Another important contribution of this study is the construction of the GRN of hiPSC chondrogenesis with the presence of minimal off-target cells, ensuring the hub genes identified are truly governing chondrogenic differentiation. In addition to conventional master TFs such as SOX9, we also identified several additional hub genes associated with chondrogenesis. For instance, the expression levels of complement C1q like 1 (*C1QL1*) were highly correlated with those of *COL2A1* in our model. *C1QL1* encodes a secreted protein with Ca^2+^ binding sites that regulate synaptogenesis in neuronal cells^40^. However, how C1QL1 affects chondrogenesis or if it plays a role in synovial joint innervation is currently unknown. In addition, our finding of the melanogenic GRN during hiPSC chondrogenesis suggests an off-target cell fate decision in differentiation. This result is further corroborated by the study of Yamashita et al.^41^ demonstrating the presence of melanin or lipofuscin on the surface of hiPSC-derived cartilage pellet using rigorous histological staining. Furthermore, we also revealed the significant association between *WNT2B* and *MITF*, providing insights into melanogenesis. Indeed, a recent study proposed genetic variants in *WNT2B* may serve as a biomarker to predict the survival rate of patients with cutaneous melanoma^42^. We also identified *WNT4* as a hub gene in the GRN of neurogenesis and observed that *WNT3A* was enriched in the cell populations expressing neural markers. These results are consistent with the previously identified roles for these WNTs in promoting forebrain development^43,44^.

Heterogenous multicellular signaling models indicate that although most WNTs were produced by off-target cells, these ligands may signal through chondrocytes. It is well recognized that WNT signaling not only blocks *SOX9* expression in limb bud mesenchymal cells but also regulates chondrocyte maturation, driving them toward hypertrophy^45,46^. In agreement with these findings, hiPSC-derived chondrogenic pellets treated with individual WNTs exhibited increased COL10A1 staining. We also demonstrated that blocking endogenous WNT signaling significantly improved chondrogenesis in hMSCs. These findings reveal the potential modulatory effects of off-target cells on chondrocytes through the WNT signaling pathway, indicating that inhibition of WNT has dual beneficial effects on hiPSC chondrogenesis as it not only removes off-target cells but also prevents chondrocyte hypertrophy.

These findings not only identify the mechanisms regulating the heterogeneity in hiPSC chondrogenesis but, more importantly, provide an enhanced chondrogenic differentiation protocol capable of generating homogenous chondrocytes by removing off-target cells without cell sorting. Furthermore, this protocol has been validated in multiple unique lines, demonstrating its robustness and efficiency in deriving chondrocytes from hiPSCs. We also established a comprehensive map of single-cell transcriptome profiles and GRNs governing cell fate decisions during hiPSC chondrogenesis. These findings provide insights into dynamic regulatory and signaling pathways orchestrating hiPSC chondrogenesis, thereby advancing a further step of cartilage regenerative medicine toward therapeutic applications. This approach also provides a roadmap for the use of single-cell transcriptomic methods for the study and optimization of other in vitro or in vivo differentiation processes.

## Methods

Key resources including antibodies, growth factors, culture reagents are listed in Supplementary Table 3.

### hiPSC lines and culture

Three distinct hiPSC lines were used in the current study: STAN, ATCC, and BJFF. STAN line was purchased from WiCell (#STAN061i-164-1), ATCC line was acquired from ATCC (#ATCCACS-1019), and BJFF was obtained from the Genome Engineering and iPSC Core at Washington University in Saint Louis. All three lines were reprogrammed by Sendai virus from human foreskin fibroblasts and confirmed to be karyotypically normal and mycoplasma free. STAN and BJFF hiPSCs were maintained on vitronectin coated 6-well plates (﻿Thermo Fisher Scientific, #A31804) in Essential 8 Flex medium (Thermo Fisher Scientific, #A2858501). ATCC hiPSCs were cultured on CellMatrix Basement Membrane Gel coated 6-well plates (﻿ATCC, #ACS3035) in Pluripotent Stem Cell SFM XF/FF medium (ATCC, #ACS3002). Cells were fed daily and passaged with ReLeSR (STEMCELL Technologies, #05872). All hiPSC lines were maintained below passage 30.

### hMSCs and culture

Discarded and deidentified waste tissue from the iliac crests of adult bone marrow transplant donors was collected in accordance with the institutional review board of Washington University in Saint Louis. Human bone marrow-derived MSCs (hMSCs) were isolated by their physical adherence to plastic culture vessels^47^. Cells were expanded and maintained in an expansion medium consisting of DMEM-low glucose (Thermo Fisher Scientific, #11885092), 1% penicillin/streptomycin (P/S, Thermo Fisher Scientific, #15140-122), 10% lot-selected FBS (Atlanta Biologicals, #S11550), and 1 ng ml^−1^ basic fibroblast growth factor (FGF) (R&D Systems, #233-FB). Three individual donors were used as biologic replicates in subsequent experiments (Supplementary Table 4).

### Mesodermal differentiation

hiPSCs were induced into mesodermal differentiation in monolayer at 40% confluency^7^. Each day, cells were rinsed with a wash medium consisting of 50% IMDM GlutaMAX (IMDM, Fisher Scientific, #31980097) and 50% Ham’s F12 Nutrient Mix (F12, Fisher Scientific, #31765092) to remove the previous medium. hiPSCs were then fed daily to sequentially drive mesodermal differentiation similar to those identified in embryonic development with various sets of growth factors and small molecules supplemented in mesodermal differentiation medium consisting of equal parts of IMDM and F12 with 1% chemically defined lipid concentrate (Gibco), 1% insulin/human transferrin/selenous acid (ITS+, Corning, #354352), 1% P/S (Thermo Fisher Scientific, #15140-122), and 450 μM 1-thioglycerol (Sigma–Aldrich, #M6145). Cells were induced to the anterior primitive streak with 30 ng ml^−1^ of Activin A (R&D Systems, #338-AC), 4 µM CHIR99021 (Stemgent, #04-0004), and 20 ng ml^−1^ human FGF-2 (R&D Systems, #233-FB-025/CF) for 24 h. On the second day, cells were driven to paraxial mesoderm with 2 µM SB-505124 (SB5; Tocris, #3263), 3 µM CHIR99021, 20 ng ml^−1^ human FGF-2, and 4 µM dorsomorphin (DM; Stemgent, #04-0024). Then, cells were treated with 2 µM SB5, 4 µM DM, 1 µM Wnt-C59 (C59; Cellagent Technology, #C7641-2s), and 500 nM PD173074 (Tocris, #3044) to become early somite on the third day. For the fourth through sixth days, cells were driven to the sclerotome with daily feedings of 2 µM purmorphamine (Stemgent, #04-0009) and 1 µM C59. Finally, for six days, cells were driven to the Cp stage with 20 ng ml^−1^ of human bone morphogenetic protein 4 (BMP4; R&D Systems, #314-BP-010/CF) daily (Supplementary Fig. 1A).

At each stage, cells were dissociated using TrypLE (Gibco, #12604013) at 37 °C for 3 min followed by adding an equal part of neutralizing medium consisting of DMEM/F-12, GlutaMAX^TM^ (DMEM/F12; Thermo Fisher Scientific, #10565042) with 10% FBS (Atlanta Biologicals) and 1% P/S. The dissociated cells were either used for bulk RNA-seq, scRNA-seq, chondrogenic differentiation, or fluorescence-activated cell sorting (FACS) as appropriate.

### Chondrogenic differentiation

Cells dissociated at the Cp stage were resuspended at 5 × 10^5^ cells per mL in chondrogenic medium consisting of DMEM/F-12, 1% FBS, 1% ITS+, 55 µM β-mertcaptoethanol, 100 nM dexamethasone (DEX; Sigma-Aldrich, #D4902), 1% NEAA (Gibco, #11140050), 1% P/S, 10 ng ml^−1^ human transforming growth factor-beta 3 (TGF-β3; R&D Systems, #243-B3-010), 50 μg ml^−1^ l-ascorbic acid 2-phosphate (ascorbate; Sigma-Aldrich, #A8960), and 40 μg ml^−1^ l-Proline (proline; Sigma-Aldrich, #P5607). Cells were then centrifuged for 5 min at 300 × *g* to form a pellet. Chondrogenic pellets were cultured at 37 °C for up to the timepoints required for various experiments.

On the day of collection for bulk RNA-seq experiments, 3–4 pellets per experimental group were pooled together and washed once with phosphate-buffered saline (PBS), snap-frozen in 300 µl of Buffer RL (﻿Norgen Biotek), and stored at −80 °C until processing for RNA extraction. At harvesting time points for scRNA-seq experiments, 6–8 pellets per experimental group were pooled and digested with 0.04% Type II collagenase solution in DMEM/F12 for 1 h. Cells were washed once with PBS, resuspended in standard freezing medium, and stored in liquid nitrogen until needed.

### C59 and ML329 treatment for WNT and MITF inhibition

For C59 treatment for WNT inhibition during chondrogenesis, pellets were treated with either 10 ng ml^−1^ TGF-β3 (control group) or a combination of 10 ng ml^−1^ TGF-β3 and 1 μM C59 in a chondrogenic medium from d0 to d42 as appropriate. For C59 and ML329 treatment (ML, Axon Medchem, HY-101464) for WNT and MITF inhibition during chondrogenesis, pellets were treated with either 10 ng ml^−1^ TGF-β3 (control group), a combination of 10 ng ml^−1^ TGF-β3 and 1 μM ML, a combination of 10 ng ml^−1^ TGF-β3 and 1 μM C59, or a combination of 10 ng ml^−1^ TGF-β3, 1 μM ML and 1 μM C59 in chondrogenic medium from d0 to d42 as appropriate.

### WNT ligands treatment during chondrogenesis

For WNT ligands treatment during chondrogenesis, pellets were treated with either 10 ng ml^−1^ TGF-β3 (control group) or a combination of 10 ng ml^−1^ TGF-β3 and 100 ng ml^−1^ individual WNT ligand (WNT2B, WNT3A, WNT4, WNT5B, or WNT7B, all from R&D system) in chondrogenic medium from d0 to d42 as appropriate. For WNT ligands treatment during the Cp stage, cells were supplemented with either 20 ng ml^−1^ BMP4 (R&D Systems, #314-BP-010) alone (control group), a combination of 20 ng ml^−1^ BMP4 and 1 μM C59, or a combination of 20 ng ml^−1^ BMP4 and 100 ng ml^−1^ WNT3A (R&D Systems, #5036-WN-010) in mesodermal differentiation medium from d7 to d12.

### Animal experiments

All animal procedures were approved by the Institutional Animal Care and Use Committee (IACUC) at Washington University in Saint Louis. Male NSG mice (NOD.Cg-*Prkdc*^*scid*^ *Il2rg*^*tm1Wjl*^/SzJ, #005557, Jackson laboratory) at age of 18–20 weeks old were used for human xenograft implantation in the dorsal region (subcutaneous) or in osteochondral defects in the knee joints of mice. Mice were housed under a 12 h light/12 h dark cycle with ambient temperature and humidity. NSG mice were anesthetized with 3% isoflurane in oxygen for all surgical procedures. For subcutaneous implantation, the skin was shaved and sterilized over the implantation site using standard sterile techniques. A mid-scapular incision was made, and a hemostat was inserted into the skin incision to create a pocket for implantation. A d14 hiPSC chondrogenic pellet was then inserted into the pockets. The incision of the skin was closed with 8-0 suture with taper point (PolysorbTM, Covidien, #L-2800). Tissue adhesive was applied to the skin wound area. For implantation in osteochondral defects in the knee, a 3 mm long medial parapatellar incision was made in the left hindlimb, and the knee joint was exposed via lateral dislocation of the patella. An osteochondral defect (1 mm in diameter and 1 mm in depth) in the trochlear groove of the femur was created by a 1 mm micro bone drill (Roboz, #RS-6300A). All debris was removed by sterile PBS washes. Mild hemorrhage from the fat pad was controlled by epinephrine 1:1000 (International Medication Systems, #491590) followed by sterile PBS wash. A d14 hiPSC chondrogenic pellet was implanted into the defect, and the patella was repositioned to its original anatomical location. Mice with osteochondral defects that did not receive pellet implantation were used as a control group. After implantation, the subcutaneous layer and skin were closed with 8-0 suture with a tapered point followed by the application of tissue adhesive to the skin wound area. After surgery, the mice were allowed to move freely within their cages. After 14 and 28 days post implantation, mice were sacrificed for pellet harvest for histological analysis.

### RNA isolation, library preparation, and bulk RNA-seq

To determine transcriptome profiles over the course of differentiation, three hiPSCs lines (ATCC, BJFF, and STAN) as biological replicates at various differentiation stages (6 mesodermal and 5 chondrogenic stages per cell line; i.e., total 33 samples) were collected for bulk RNA-seq. Cell samples were thawed on ice, and pellet samples were homogenized with zirconia beads (BioSpec Products, # 11079110zx) and a miniature bead beater. RNA was then isolated from all samples using the ﻿Total RNA Purification Kit according to the manufacture’s protocol (Norgen Biotek, #37500). RNA was eluted in 20 μl of diethylpyrocarbonate-treated water. The quality and quantity of RNA from each sample was evaluated by RNA Analysis ScreenTape (Agilent, #5067-5576) on a bioanalyzer (Agilent 4200 Tapestation). Only samples with a RIN value larger than 0.8 were submitted to the Genome Technology Access Center (GTAC sequencing core) at Washington University in St. Louis for library preparation and bulk RNA-seq. Libraries were prepared using TruSeq Stranded Total RNA with Ribo-Zero Gold kit (Illumina). Sequencing was performed on a HiSeq2500 instrument (Illumina) (1 × 50 bp reads) with a sequencing depth of 30 million reads per sample.

### Preprocessing of bulk RNA-seq data

Reads were processed using an in-house pipeline and open-source R packages as previously described^48^. Raw reads were first trimmed using Cutadapt to remove low-quality bases and reads^49^. After trimming, processed reads were aligned to the human reference genome GRCh38 (version 90) by STAR^50^, and the number of aligned reads to each annotated genes or transcripts (GENCODE v21) was performed using *featureCounts* from the Subread package (v1.4.6)^51^.

### DEGs and GO enrichment analysis and of bulk RAN-seq data

After quality control, un-normalized gene counts were read into the DESeq2 R package by *DESeqDataSetFromMatrix* function as instructed by the package tutorial^52^. Genes that were expressed by less than ten cells were then removed. Next, we used *DESeq* and *results* functions which implement Wald test in DESeq2 to determine the DEGs between two consecutive differentiation stages. In this process, the estimation of size factors (i.e., controlling for differences in the sequencing depth of the samples), the estimation of dispersion values for each gene, and fitting a generalized linear model were performed. The gene counts were also averaged from three hiPSC lines. Top 20 DEGs between two consecutive stages were selected and visualized using ComplexHeatmap R package. To observe the temporal expression of a given gene for each hiPSC line, the count matrix was regularized-logarithm transformed via *rlog* function first, and we used *plotCounts* function in DESeq2 to visualize the expression pattern of the gene. Furthermore, regularized-logarithm transformed counts were also used for PCA, and PCA plots were visualized by *ggplot* function in ggplot2 R package^53^.

We next performed GO enrichment analysis of the genes in mesodermal and chondrogenic stages using GAGE R package (Generally Applicable Gene-set/Pathway Analysis), whose algorism evaluates the coordinated up- or down-differential expression over gene sets defined by GO terms^13^. Significantly upregulated GO terms with their associated *p* values in biological process, molecular function, and cellular component were plotted by GraphPad Prism (version 8.0; GraphPad Software). Furthermore, GAGE analysis also reveals that 134 out of 205 genes defined by GO term cartilage development (GO:0051216) were significantly increased during our differentiation process. Thus, a heatmap was generated to investigate the expression levels of these genes at various stages using ComplexHeatmap R package^54^.

### 10× chromium platform scRNA-seq

Cells were thawed at 37 °C and resuspended in PBS with 0.04% bovine serum albumin at a concentration of 2000 cells per μl. Cell suspensions were submitted to the GTAC sequencing core at Washington University in St. Louis for library preparation and sequencing. In brief, 10,000 cells per sample were loaded on a Chromium Controller (10× Genomics) for single capture. Libraries were prepared using Single Cell 3′ Library & Gel Bead Kit v2 (#120237 10× Genomics) following the manufacture’s instruction. A single cell emulsion (Gel Bead-In-EMulsions, GEMs) is created by making barcoded cDNA unique to each individual emulsion. A recovery agent was added to break GEM and cDNA was then amplified. A library is produced via end repair, dA-tailing, adapter ligation, post-ligation cleanup with SPRIselect, and sample index PCR. The quality and concentration of the amplified cDNA were evaluated by Bioanalyzer (Agilent 2100) on a High Sensitivity DNA chip (Agilent, #5065-4401). The only cDNA with an average library size of 260–620 bp were used for sequencing. Sequencing was performed by Illumina HiSeq2500 with the following read length: 26 bp for Read1, 8 bp for i7 Index, and 98 bp for Read2. We generally acquired ~180 million reads per library (sample). A species mixing experiment (mouse adipose stem cells and human iPSCs, 1:1 mixture) was also performed prior to running on the actual sample to ensure good quality of single-cell capture (i.e., cell doublet rate < 5%).

### Preprocessing of scRNA-seq data

Paired-end sequencing reads were processed by Cell Ranger (10× Genomics software, version 2.0.0). Reads were aligned to the GRCh38 (version 90) for genome annotation, demultiplexing, barcode filtering, and gene quantification. Cell Ranger also removes any barcode that has less than 10% of the 99th percentile of total unique molecular identifiers (UMI) counts per barcode as these barcodes are considered to be associated with empty droplets. After this quality control, gene barcode matrices for each sample were generated by counting the number of UMIs for a given gene (as a row) in the individual cell (as a column). For each sample, ~1300–2500 cells were captured.

### Unsupervised clustering analysis and annotation

To assess the difference in the composition of cell populations, we performed global unsupervised clustering analysis for our scRNA-seq datasets. First, gene barcode matrices were input into the Seurat R package (version 2.4)^15^. We then removed the low-quality cells with less than 200 or more than 7000 detected genes or if their mitochondrial gene content was more than 5%. Note that the cutoff criteria were adjusted in few cases due to the sequencing depth and the variations in mitochondrial gene content from datasets. Genes that were detected in less than three cells were filtered out. After filtering out low-quality cells or cell doublets, the gene expression was then natural log-transformed and normalized for scaling the sequencing depth to 10,000 molecules per cell. Next, to reduce the variance introduced by unwanted sources, we regressed out variation in gene expression driven by cell cycle stages and mitochondrial gene expression with *vars.to.regress* argument in function *ScaleData* in Seurat. We then used the *FindVariableGenes* function in Seurat to identify highly variable genes across cells for downstream analysis. These steps resulted in (1) a total of 8547 cells with an average of 1882 highly variable genes from stages of hiPSCs, Sclerotome, and Cp stages, (2) a total of 10,648 cells with an average of 2061 highly variable genes from stages of TGF-β3-treated pellets (d1, d3, d7, d14, d28, and d42), and (3) total 7997 cells with average 1886 highly variable genes from TGF-β3+C59-treated pellets (d7, d14, d28, and d42) for downstream analysis. Detailed cell numbers that passed quality control steps for each stage are listed in Supplementary Table 5. Dimensionality reduction on the data was then performed by computing the significant principal components on highly variable genes. We then performed unsupervised clustering by using the *FindClusters* function in Seurat with the resolution argument set to 0.6, and clusters were then visualized in a tSNE plot^55^.

DEGs among each cell cluster were determined using the *FindAllMarkers* function in Seurat. DEGs expressed in at least 25% of cells within the cluster and with a fold change of more than 0.25 in natural log scale were considered to be marker genes of the cluster. To determine the biological functions of the marker genes from a given cluster, we performed GO enrichment analysis by using The DAVID Gene Functional Classification Tool (http://david.abcc.ncifcrf.gov; version 6.8)^56^. By comparing these unique biological GO terms with existing RNA-seq datasets and the literature, we were able to annotate cell clusters. In addition, the top 10 enriched GO terms from the biological function category with associated p values were visualized GraphPad Prism (version 8.0; GraphPad Software).

### Cell cycle analysis of scRNA-seq data

*Cell cycle scoring* function in Seurat was used to determine a cell cycle score on each cell according to its gene expression of G2/M phase (54 genes) and S phase (43 genes) markers^57^. Based on this scoring system, fractions of each cell cluster with a given cell cycle score in total cell population were computed.

### CCA for integrated analysis of multiple scRNA-seq datasets

To compare cell types and to identify their associated DEGs between distinct experimental conditions such as batch effect, C59 treatment, or differentiation stages (i.e., time points), we applied CCA, a computational strategy implemented in Seurat for integrated analysis of multiple datasets. First, the top 1000 highly variable genes from each dataset were selected. We then use the *RunCCA* function or *RunMultiCCA* function (if more than two datasets) to identify common sources of variation resulting from experimental conditions and to merge the multiple objects into a single dataset. We next determined the top principal components of the CCA by examining a saturation in the relationship between the number of principal components and the percentage of the variance explained using the *MetageneBicorPlot* function. By using selected top principal components, we aligned the CCA subspaces with the *AlignSubspace* function, which returns a new dimensional reduction matrix allowing for downstream clustering and DEG analyses. DEG analysis was performed on the cells from different datasets but grouped in the same cluster (i.e., conserved cell types between two conditions) after CCA alignment. The methods for cell clustering, identification of conserved cell types and DEGs, as well as annotation of cell clusters were similar to the ones mentioned previously. DEGs in each conserved cell type in response to differentiation stages or C59 treatment were visualized by ComplexHeatmap R package^54^. In some cases, genes of interest such as WNTs and various lineage markers were also visualized using the *FeatureHeatmap* and *DotPlot* function in Seurat.

### Pseudotemporal ordering and lineage trajectories

We used the Monocle2 R package to reconstruct differentiation trajectories by computing and ordering the sequence of gene expression changes of the cells collected from different time points in an unsupervised manner^16,58^. First, scRNA-seq datasets from different timepoints underwent several quality control steps as mentioned previously. These multiple scRNA-seq datasets were then merged into one single object using the *MergeSeurat* function in Seurat. The merged matrix was then converted into a Monocle object using *importCDS* and *newCellDataSet* functions in Monocle2. We then identified a set of DEGs between the cells collected at the beginning of the process to those at the end using *differentialGeneTest* function with argument qval < 0.01 in Monocle. The dimensions of the dataset were then reduced using the first two principal components with the “DDRTree” method. Next, we used *orderCells* function to order the cells based on the selected DEGs and the trajectory of the cells was visualized by the *plot_cell_trajectory* function in Monocle. The temporal expression of the gene of interests was visualized using the *plot_genes_in_pseudotime* function in Monocle. Additionally, to observe dynamic changes in the expression levels of the genes that were branch dependent (i.e., along with specific lineage), we used *plot_genes_branched_heatmap* function in Monocle to construct a special type of heatmap in which genes that had similar lineage-dependent expression patterns were clustered together.

### WGCNA reconstruction of GRNs and hub genes

We used WGCNA, an algorithm implemented in the WGCNA R package, to reconstruct GRNs and to identify their associated hub genes that regulate cell differentiation^19^. First, the dataset of interest (e.g., a given time point) created in Seurat was converted into a plain matrix for a given gene (in the column) in an individual cell (in a row). The dataset was then cleaned by removing cells with too many missing values using the *goodSamplesGenes* function in WGCNA. Next, we used the *pickSoftThreshold* function in WGCNA to determine the proper soft-thresholding power (*β*) that fits the criterion of the approximate scale-free topology of the network, and an adjacency matrix was then built with soft-thresholding power of eight in our study. Hierarchical clustering and GRN were constructed by using *blockwiseModules* function with arguments *TOMType* set to unsigned, *networkType* set to sign, and *mergeCutHeight* set to 0.25 in WGCNA. Modules containing genes that were highly associated with each other were identified in this process. Gene lists of interesting modules were extracted and submitted to DAVID for GO term analysis to retrieve their biological process and molecular functions. We then identified TFs and TF regulators from the genes based on the GO terms in molecular functions. We then selected the top 100 genes that had the highest weight (i.e., high correlation coefficient) connected to a given TF or TF regulator. Finally, the GRN based on these TFs and TF regulators then underwent cluster analysis using community cluster (GLay)^59^ and was then visualized using Cytoscape^60^. Hub genes for each GRN were identified as genes with high weight (summed correlation coefficients), high degree (summed connectivity, i.e., total numbers genes connected to this specific gene), and high betweenness centrality (BC) measure of the network. The hub gene of a given GRN was visualized by ComplexHeatmap R package^54^.

### Multicellular signaling and ligand-receptor models

To investigate the ligand–receptor interaction in heterogenous multicellular signaling systems, we used a list comprising 2557 human ligand–receptor pairs curated by Database of Ligand–Receptor Partners, IUPHAR, and Human Plasma Membrane Receptome^32,61^. We first quantified the percentage of the cells (i.e., neural cells, melanocytes, and chondrocytes) that expressed a specific WNT ligand and its associated frizzled (FZD) receptors using scRNA-seq datasets. To ensure the ligand and receptors are uniquely expressed, we required that their expression in fold change needs to more than 0.25 on a natural log scale. We then used Circlize R package to visualize the directions of the signaling in the cell type based on connections of ligand–receptor pairs^62^.

### RNA fluorescence in situ hybridization (RNA-FISH)

To validate scRNA-seq findings and to visualize the spatial distribution of *WNTs* and *COL2A1* within pellets, we performed RNA-FISH for *WNT3A*, *WNT4*, and *COL2A1* expression. d28 pellets with or without C59 treatment were harvested (*n* = 3 time point) and snap-frozen in liquid nitrogen. Pellets were cryo-sectioned at 10 μm thick and fixed using 4% paraformaldehyde in PBS on ice for 10 min. Sample pre-treatment and RNA probe hybridization, amplification, and signal development were performed using the RNAscope Multiplex Fluorescent Reagent Kit v1 (Advanced Cell Diagnostics, #320850) following the manufacturer’s instruction. Samples were imaged with multichannel confocal microscopy (Zeiss LSM 880). Tiled images with Z-stacks were taken at 20× magnification to capture the entire pellet. Maximum intensity projection, a process in which the brightest pixel (voxel) in each layer along Z direction is projected in the final 2D image, was performed using Zeiss Zen Blue (version 2.5).

### FACS for progenitors

Cells at the Cp stage with the treatment of BMP4, a combination of BMP4 and WNT3A, or a combination of BMP4 and C59 were dissociated and resuspended in FACS Buffer (PBS^−/−^ with 1% FBS and 1% penicillin/streptomycin/fungizone (P/S/F; Gibco) at approximately 40 × 10^6^ cells per ml. The cells were treated with Human Tru Stain FC X^TM^ (BioLegend, #422302) for 10 min at room temperature. Approximately, 10,000 cells in 100 µl were used for each compensation. Cells were labeled with appropriate antibodies including their associated isotype control (FITC-CD45, #304006; PE/Cy7-CD146, #361008; PE-CD166, #343904, all from BioLegend). Cells were incubated for 30 min at 4 °C and washed with FACS buffer twice. Samples were resuspended in a sorting medium consisting of DMEM/F12 with 2% FBS, 2% P/S/F, 2% HEPES (Gibco), and DAPI (BioLegend, #422801) at 4 × 10^6^ cells per ml and filtered through a 40 µm cell strainer. Cells were stored on ice prior to sorting. Five microliters of all antibodies were used per million cells in 100 µl staining volume; 10 µl of Tru Stain FC X^TM^ was used per million cells in 100 µl staining volume. DAPI was used at 3 µM. An Aria-II FACS machine was used to compensate for the color overlapping and to gate the samples. Data were analyzed using FlowJo software (version 10.5.3).

### Histology

Pellets were collected in 10% neutral buffered formalin for fixation for 24 h. Pellets were then transferred to 70% ethanol, dehydrated, and embedded in paraffin wax. Pellet blocks were sectioned at 8 µm thickness and stained for proteoglycans and cell nuclei according to the Safranin-O and hematoxylin standard protocol.

### Immunohistochemistry

Histologic sections (8 μm thick) of the pellets were rinsed with xylenes three times and rehydrated before labeling. Antigen retrieval was performed with 0.02% proteinase K for 3 min at 37 °C for COL2A1 and COL6A1 and with pepsin for 5 min at room temperature for COL1A1 and COL10A1 followed by peroxidase quench then serum blocking for 30 min at room temperature. Samples were labeled for 1 h with the primary antibody against COL1A1 (1:800 Abcam #90395), COL2A1 (1:10 Iowa #II-II6B3-s), COL6A1 (1:1000 Fitzgerald #70F-CR009X), and COL10A1 (1:200 Sigma #C7974) and for 30 min with the secondary antibody goat anti-mouse (1: 500, Abcam #97021) or goat anti-rabbit (1:500 Abcam #6720) as appropriate. Histostain Plus Kit (Sigma, #858943) was then used for enzyme conjugation for 20 min at room temperature followed by AEC (ThermoFisher, #001111) for 2.5 min (COL2A1 and COL6A1) or 2 min (COL1A1 and COL10A1) at RT. Finally, samples were counterstained with hematoxylin to reveal cell nuclei for 45 sec and mounted with Vector Hematoxylin QS (Vector lab, #H3404). Images were taken by the Olympus VS120 microscope (VS120-S6-W).

### Biochemical analysis of cartilaginous matrix production

Pellets were rinsed with PBS after chondrogenic differentiation and digested at 65 °C overnight in 200 µl papain solution consisting of 125 μg ml^−1^ papain (Sigma, P4762), 100 mM sodium phosphate, 5 mM EDTA, and 5 mM l-cysteine hydrochloride at 6.5 pH. Samples were stored at −80 °C before thawing to measure double-stranded DNA by ﻿Quant-iT PicoGreen dsDNA Assay Kit (ThermoFisher, #P11496) and glycosaminoglycans (GAG) by the 1,9-dimethylmethylene blue assay at 525 nm wavelength^63^. GAG content, as calculated based on the standard curve, was normalized to double-stranded DNA content to obtain the GAG/DNA ratio.

### RT-qPCR

RNA of the pellets was isolated using the ﻿Total RNA Purification Kit according to the manufacture’s protocol (Norgen Biotek, #37500). Reverse transcription of the RNA was performed using ﻿SuperScript VILO Master Mix (Thermo Fisher, # 11755050). ﻿Fast SYBR Green Master Mix (Thermo Fisher, # 4385614) was used for reverse transcription-quantitative polymerase chain reaction (RT-qPCR) according to the manufacturer’s instructions on the ﻿QuantStudio 3 (Thermo Fisher). Gene expression was analyzed using the ΔΔC_T_ method relative to undifferentiated hiPSCs with the reference gene ﻿TATA-box-binding protein (*TBP*). Sequences of primers are listed in Supplementary Table 6.

### Western blots

To examine the effect of C59 on WNT inhibition in the pellets at protein levels, Western blot analysis was performed on d28 pellets with or without C59 treatments. Six to eight pellets per experimental group were pooled and digested with 0.04% Type II collagenase solution in DMEM/F12 for 1 h. Cells were washed once with PBS and lysed in RIPA buffer (Cell Signaling Technology, #9806S) with protease inhibitor (ThermoFisher, #87786) and phosphatase inhibitor (Santa Cruz Biotechnology, #sc-45044). Protein concentration was measured using the BCA Assay (Pierce). Ten micrograms of proteins for each well were separated on 10% sodium dodecyl sulfate-polyacrylamide gel electrophoresis gels with prestained molecular weight markers (Bio-Rad, 161-0374) and transferred to a polyvinylidene fluoride (PVDF) membrane. The PVDF membrane blots were incubated overnight at 4 °C with the following primary antibodies: anti-WNT2B (1:350, Abcam, ab178418), anti-WNT3A (1:1000, Abcam, ab81614), anti-WNT4 (1:500, Abcam, ab91226), anti-WNT5B (1:500, Abcam, ab93134), anti-WNT7B (1:2000, Abcam, ab155313) and anti-GAPDH (1:30000, Proteintech 60004-1-Ig) for loading control, respectively. Affinity purified horseradish peroxidase (HRP)-linked goat anti-rabbit IgG secondary antibody (1:3000, Cell Signaling, #7074) or horse anti-mouse IgG secondary antibody (1:3000, Cell Signaling, #7076) was added and incubated for 45 min at room temperature. Immunoblots were imaged and analyzed using the iBright FL1000 Imaging System (Thermo Fisher). After the WNT proteins were imaged, the blots were then stripped by incubating with restore plus Western blot stripping buffer (ThermoFisher Scientific) at room temperature for 15 min. A full scan of all unprocessed Western blots is provided in the Source Data File.

### Statistical analysis

All data were presented as mean ± SEM. Analyses were performed using SPSS Statistics (version 25) with significance reported at the 95% confidence level. In the current study, the number of pellets per group or treatment condition is technical replicates, while the number of mice per group are biological replicates.

### Reporting summary

Further information on research design is available in the Nature Research Reporting Summary linked to this article.

## Supplementary information

Supplementary Information

Reporting Summary

## Data Availability

We acquired RNA-seq datasets of human primary chondrocytes from a previously published study (NIH Gene Expression Omnibus (GEO) accession number GSE106292)^14^, in which embryonic hind limb bud chondrocytes (age: 6 weeks, *n* = 2), adolescent knee chondrocytes (age: 17 weeks, *n* = 2), adult knee chondrocytes (age: 18–60 years, *n* = 2), and growth plate chondrocytes (age: 14 weeks, 15 weeks, and 18 weeks, *n* = 1 per age). For the datasets obtained from the previously mentioned study, gene expression counts were averaged if there were more than two samples of the same age. We also harvested chondrocytes from human costal cartilage and performed bulk RNA-seq on these samples (age: ~70 years, *n* = 3). However, it was challenging to collect rib cartilage from young healthy donors; thus, aged 70-year-old costal cartilages were used. To compare the difference between the phenotypes of chondrocytes derived from hiPSCs and hMSCs, we also used bulk RNA-seq datasets of hMSC chondrogenesis from our recent study (GEO accession number GSE109503)^47^. For the present study, our bulk RNA-seq and scRNA-seq datasets are available on GEO accession number GSE160787. Source data are provided with this paper.

## Source data

Source Data
