# Supplementary material for: Single cell transcriptomic analysis of human pluripotent stem cell chondrogenesis
Source: Nat Commun. 2021 Jan 13;12:362. doi: 10.1038/s41467-020-20598-y (PMC7806634; doi:10.1038/s41467-020-20598-y)
Supplement: Supplementary file 1 — Supplementary Information [file 41467_2020_20598_MOESM1_ESM.pdf]

**Supplementary Information**

# Single Cell Transcriptomic Analysis of Human Pluripotent Stem Cell Chondrogenesis

Chia-Lung Wu[1,2,5,6], Amanda Dicks[1,2,3,5], Nancy Steward[1,2], Ruhang Tang[1,2], Dakota B. Katz[1,2,3], Yun-Rak Choi[1,2,4], and Farshid Guilak[1,2,3*]

[1]Dept. of Orthopaedic Surgery, Washington University, St. Louis, MO 63110

[2]Shriners Hospitals for Children – St. Louis, St. Louis, MO 63110

[3]Dept. of Biomedical Engineering, Washington University, St. Louis, MO 63110

[4]Dept. of Orthopaedic Surgery, Yonsei University, Seoul, South Korea

[5]Authors contributed equally to this work

[6]Current location: Department of Orthopaedics and Rehabilitation, Center for Musculoskeletal Research, University of Rochester, NY 14627

*Correspondence: Farshid Guilak
guilak@wustl.edu

Center of Regenerative Medicine
Washington University, St. Louis
Campus Box 8233
McKinley Research Bldg, Room 3121
St. Louis, MO 63110-1624

Supplementary Information includes supplementary note, supplementary figures and supplementary tables.

**Supplementary Note 1**

In addition to exogenous TGF-β3 stimulation, endogenous signaling from other members of the TGF-β superfamily (e.g., bone morphogenetic proteins (BMPs), growth and differentiation factors (GDFs)) is also essential for regulating chondrogenesis. To investigate how C59 treatment alters gene expression profiles of these families and their associated receptors (including Type I and Type II receptors), we used CCA to align chondrocytes populations and mesenchymal cells from d14 pellets with or without C59 treatment. We also observed that C59 treatment decreased *BMP2*, *BMP4*, *BMP6,* and *BMP7* gene expression, but it increased *GDF5* and *GDF10* gene expression. For receptors, C59 treatment enhanced expression levels of *BMPR1B* and *ACVR1*, but it decreased *BMPR2*, *ACVR2A*, and *ACVR2B* expression (**Supplementary Fig. 11A-B and 12A-B**). *BMP8A*, *BMP10*, *BMP11*, and *BMP15*, as well as *GDF2*, *GDF4*, *GDF6*, and *GDF8* were not detected in our datasets.

To investigate how C59 treatment affects the percentage of cells expressing genes of interest within a specific chondrocyte subpopulation, we used *BMP4*, *GDF5*, *BMPR1B* (type I receptor), and *BMPR2* (type II receptor) as examples (**Supplementary Fig. 10B-E**). For BMP4, C59-treated pellets had a decreased percentage of *BMP4*-expressing cells within all chondrocyte subpopulations except *ISG15/IFI6/MX1*+ mature-hypertrophic chondrocytes as compared to pellets treated with TGF-β3 only condition. In addition, C59-treated pellets also demonstrated a remarkably increased percentage of *GDF5* and *BMPR1B* expressing cells within all chondrocyte populations versus TGF-β3-treated pellets. Furthermore, C59 decreased the percentage of the cells expressing *BMPR2* in *LECT1/EPYC/FRZB*+ early-mature, *ISG15/IFI6/MX1*+ mature-hypertrophic chondrocytes, *BNIP3/FAM162*+ chondrocytes, and *HMGB2/CDK1*+ and *UBE2C/CCNB1*+ proliferating chondrocytes. Interestingly, it appeared that C59 treatment did not significantly affect the contribution of a chondrocyte subpopulation to the cells expressing a given gene (i.e., the cells expressing the genes of interests mainly came from *LECT1/EPYC/FRZB*+ early-mature chondrocytes regardless of the treatments as presented in the pie charts in **Supplementary Fig. 10B-E**).

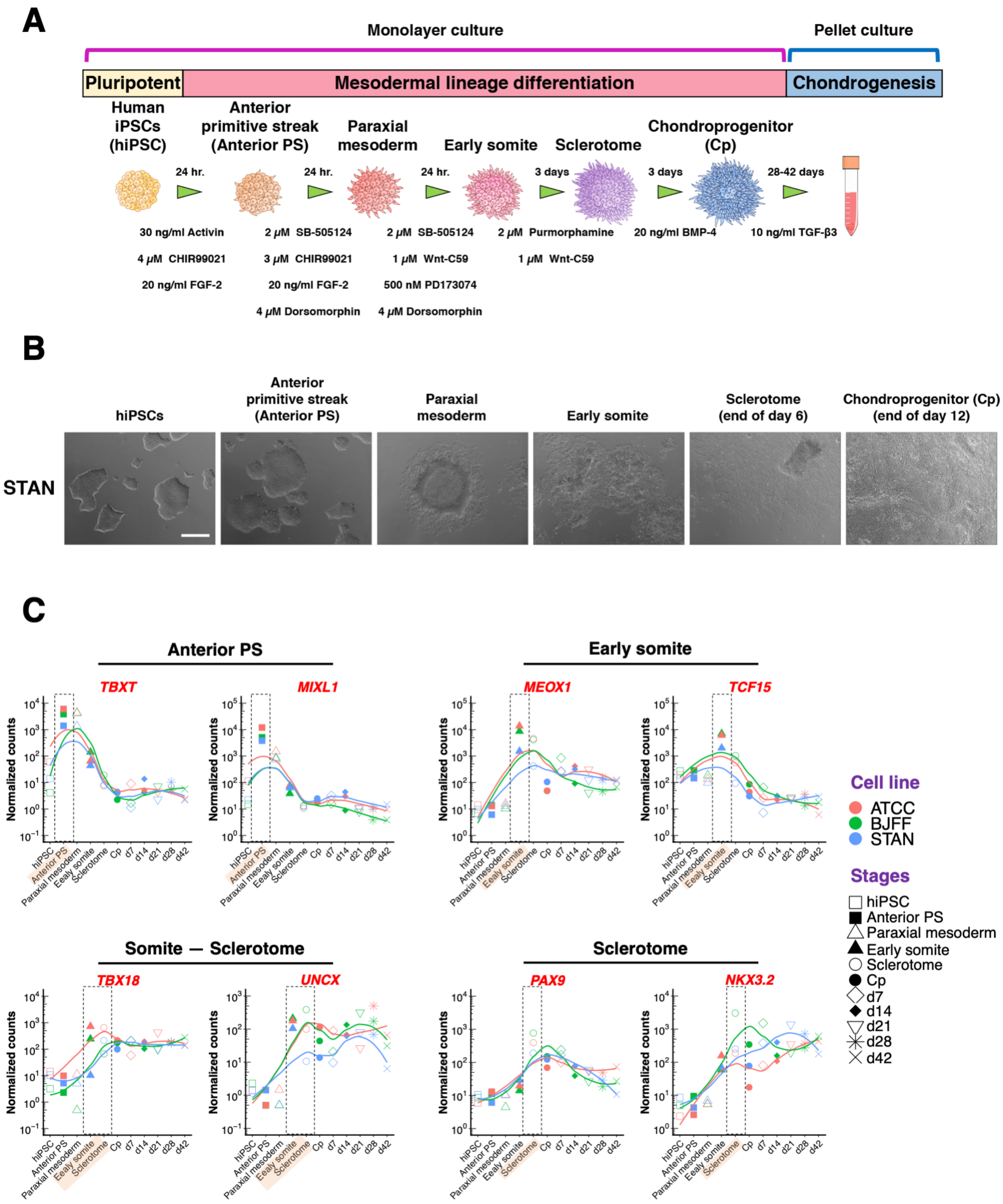


**Supplementary Figure 1. Step-wise differentiation of hiPSCs toward chondrocytes via specification of mesoderm**

(A) Differentiation protocol of hiPSCs into chondrocytes.

(B) Cell morphology at each stage during mesodermal differentiation. Please note that low cell density at hiPSC stage is required to obtain successful mesodermal differentiation. Scale bar = 500 μm.

(C) Up-regulation of stage-specific markers for 3 unique hiPSC lines.

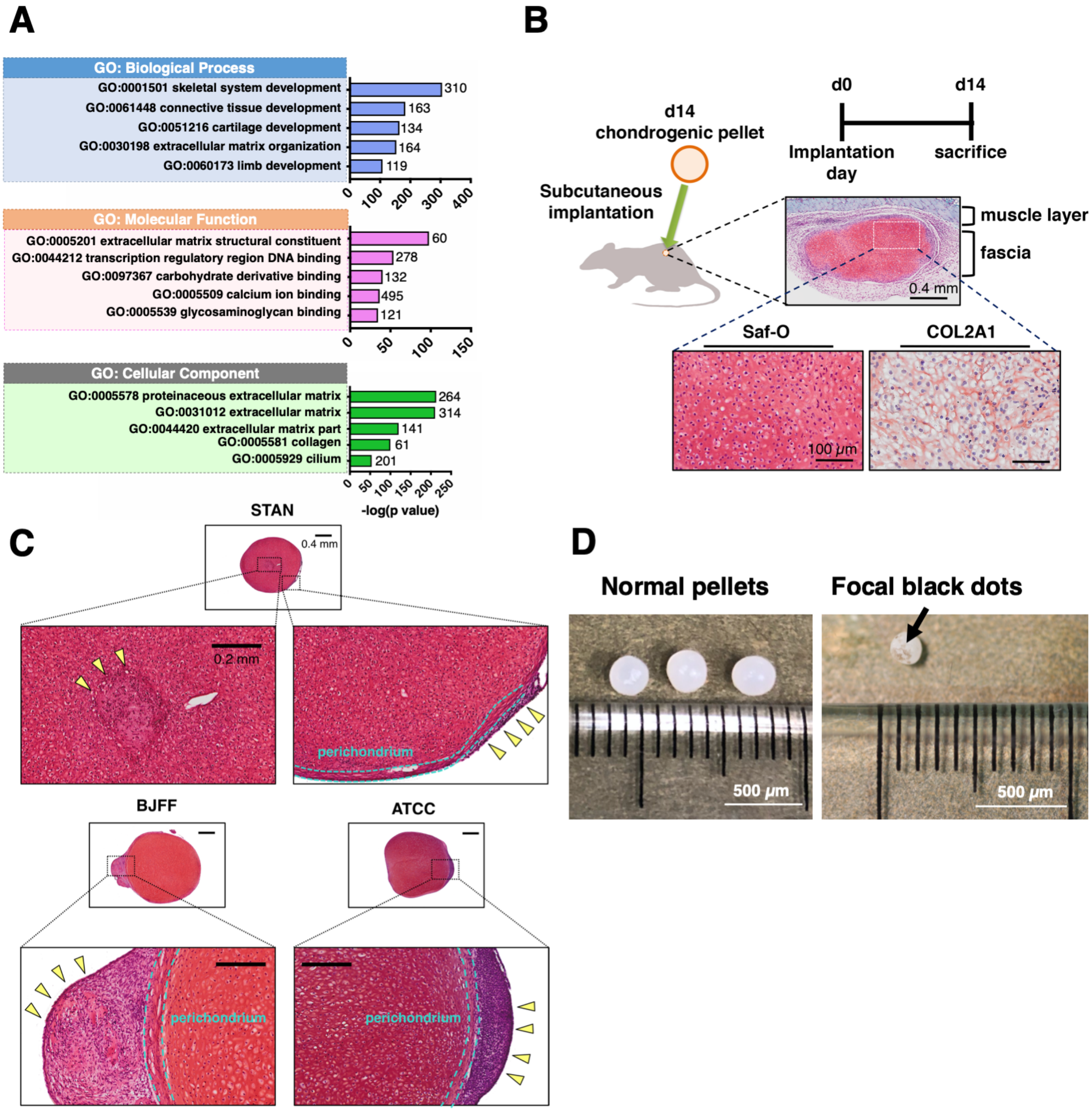


**Supplementary Figure 2. GO enrichment analysis of bulk RNA-seq data and subcutaneous implantation of hiPSC-derived chondrocytes in mice**

(A) GO enrichment analysis of bulk RNA-seq data showing that up-regulated genes were involved in skeletal system and cartilage development.

(B) d14 chondrogenic pellets maintained a cartilage phenotype indicated by intense Saf-O and COL2A1 staining after 14 days of subcutaneous implantation in mice. n = 3 mice.

(C) The off-target cells (mostly located at the edge of perichondrium, yellow arrowheads) were observed in the pellets derived from 3 distinct hiPSC lines.

(D) Focal black dots were occasionally observed on the surface of the pellets.

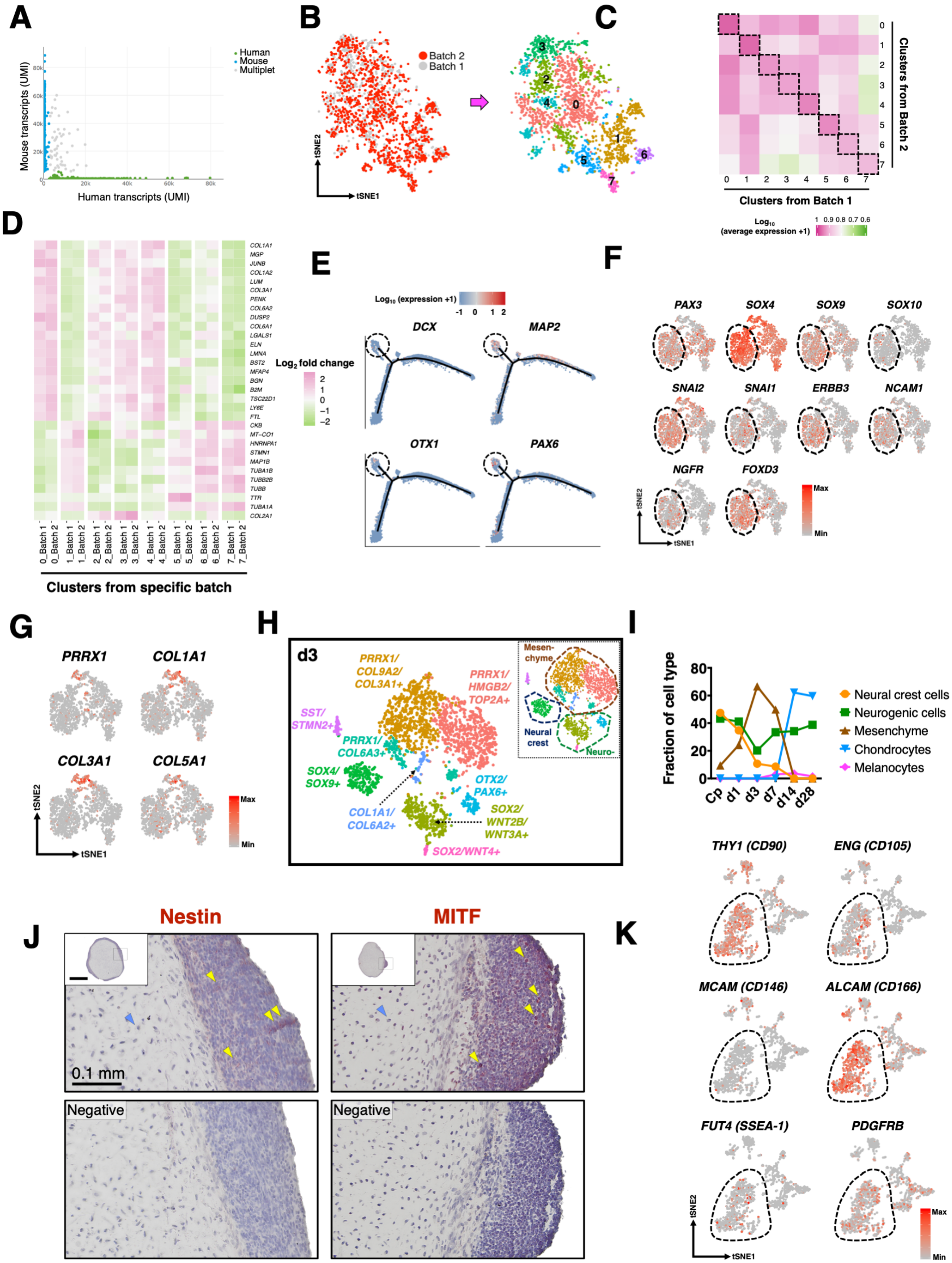


**Supplementary Figure 3. Analysis of scRNA-seq data reveals diverse cell populations in hiPSC-derived chondrogenic pellets**

(A) scRNA-seq of mixed specie samples showing low multiplet rates (< 2.7%).
(B) CCA of scRNA-seq data from d28 chondrogenic pellets from 2 independent experiments (i.e., 2 batches). 8 conserved cell clusters were identified in both batches.
(C) Cells in the same cluster from different batches exhibited high correlation in their gene expression (Spearman's rank coefficient $r_s > 0.87$ for all clusters).
(D) Cells in the clusters from distinct batches demonstrated similar gene expression patterns.
(E) Additional neural cell markers such as *DCX*, *MAP2*, *OTX1,* and *PAX6* were also enriched in the branch of neurogenic differentiation.
(F) *SOX4*+ and *SOX4/SOX9*+ cells at the Cp stage had high expression of neural crest cell markers. A total of 1,888 cells at the Cp stage that passed quality control was analyzed.
(G) Cells that are enriched for *PRRX1, COL1A1, COL3A1,* and *COL5A1* were annotated as "mesenchyme" at the Cp stage. A total of 1,888 cells at the Cp stage that passed quality control was analyzed.
(H) Three major cell populations observed in d3 pellets. A total of 2,485 cells from d3 pellets that passed quality control was used to generate the tSNE plot.
(I) Fraction of major cell types over the course of differentiation (Cp – d28). A total of 11,208 cells from the Cp stage to d28 pellets was analyzed.
(J) IHC against nestin and MITF confirms the presence of neural cells and melanocytes in pellets.
(K) Mesenchymal cells in d14 pellets expressed several conventionally recognized MSC markers. However, whether these mesenchymal cells exhibit multipotency like MSCs requires further investigation. A total of 2,148 cells from d14 pellets was analyzed.

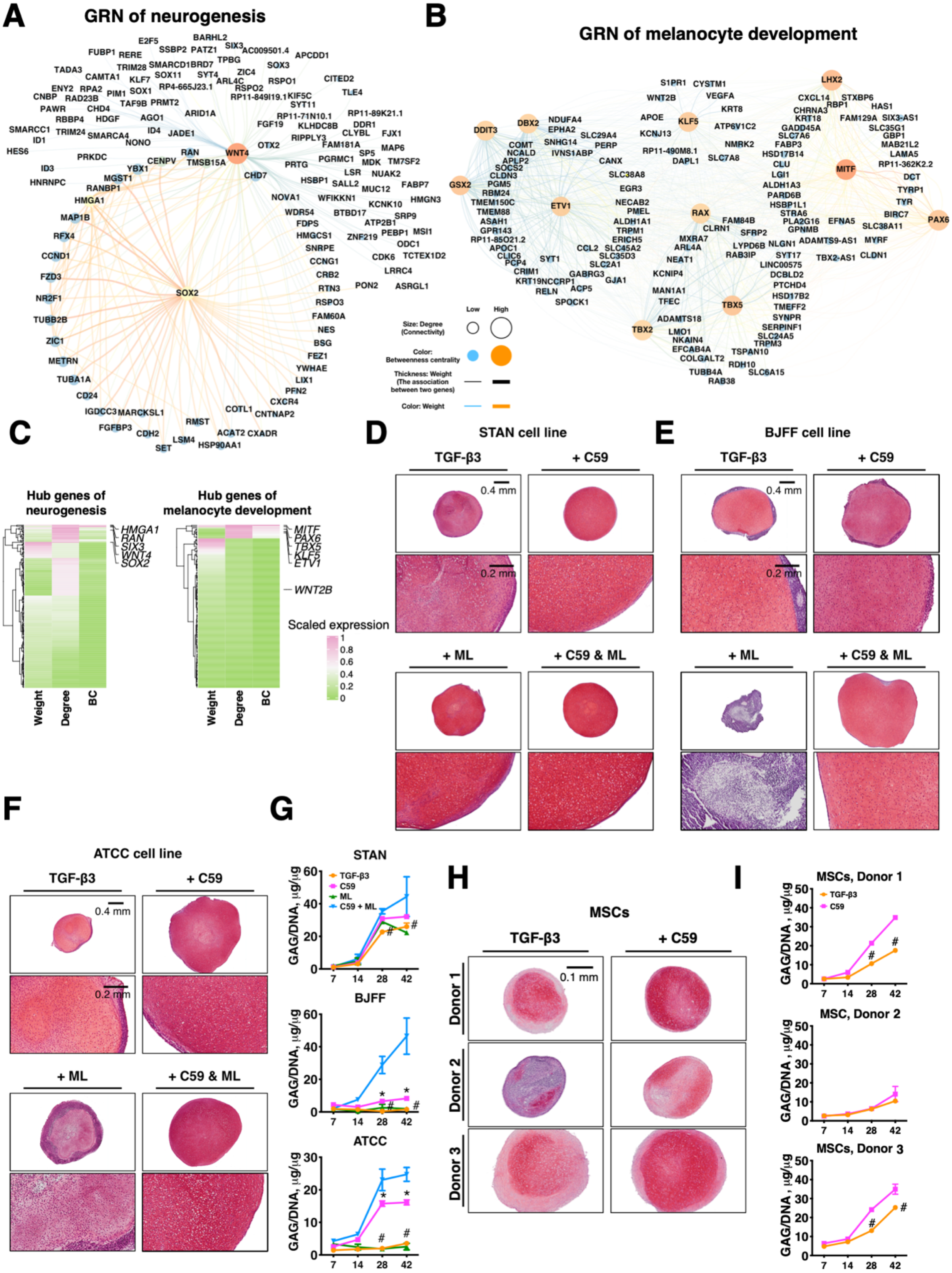


Supplementary Figure 4. WGCNA reconstructed GRNs of neurogenesis and melanogenesis and identified the hub genes in each network

(A-B) GRNs of neurogenesis and melanogenesis. Topological analysis (community cluster) was performed to visualize subnetworks.
(C) *WNT4* was among the hub genes in the GRN of neurogenesis while *WNT2B* was associated with the GRN of melanocyte development.
(D-F) Representative d28 pellet images showing that C59 or a combination of C59 and ML treatment during pellet culture enhanced the homogeneity of chondrogenesis by removing off-target cells. This was validated in 3 unique hiPSC lines.
(G) The pellets treated with C59 or a combination of C59 and ML treatment exhibited significantly increased GAG/DNA ratios compared to the pellets treated with ML and the pellets treated TGF-β3. * C59 vs. TGF-β3 ($p = 0.01$) at a specific timepoint. # C59 + ML vs. TGF-β3 ($p = 0.001$) at a specific timepoint. Mean ± SEM. n = 4 pellets per treatment condition. One-way ANOVA with *Fisher's LSD* was performed at d28 and d42.
(H) hMSCs harvested from 3 distinct donors exhibited increased chondrogenesis when treated with C59 during pellet culture.
(I) hMSCs harvested from donor 1 and donor 3 had significantly increased GAG/DNA ratios when treated with C59 compared to with TGF-β3 alone. # C59 vs. TGF-β3 ($p = 0.01$) at specific time point. Mean ± SEM. n = 4 pellets per treatment condition. Two-tailed Student's t-test was performed at d28 and d42.

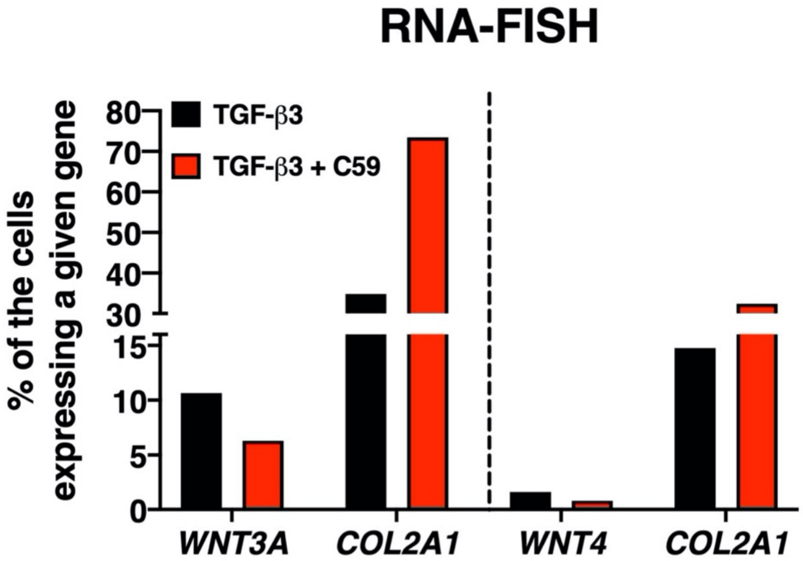


**Supplementary Figure 5. Semi-quantification of RNA-FISH against WNTs and COL2A1.**
C59-treated pellets showed decreased *WNT3A* and *WNT4* expression but increased *COL2A1* RNA-FISH labeling versus TGF-β3-treated pellets.

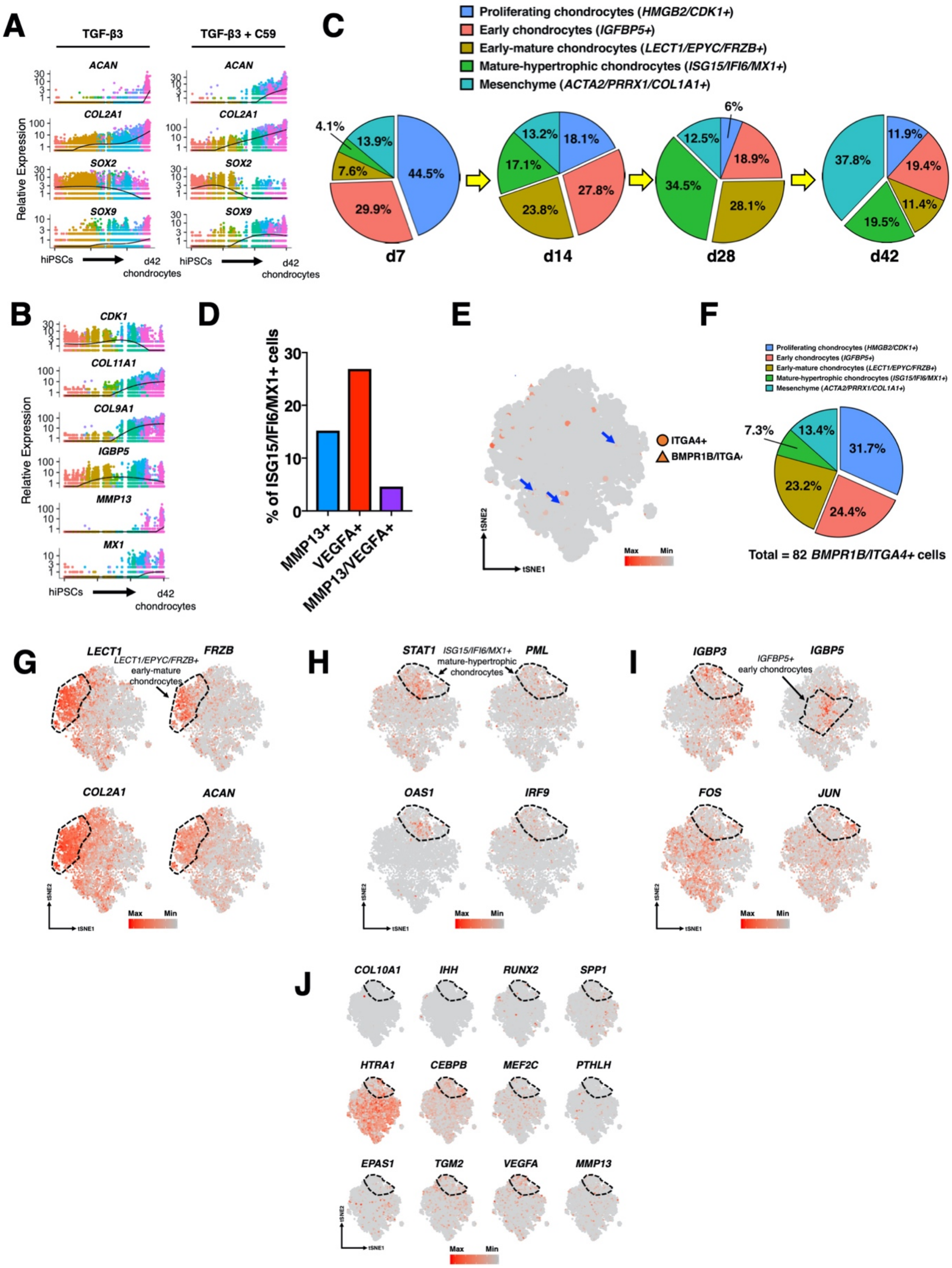


**Supplementary Figure 6. Multiple CCA alignment of d7-d42 pellets reveals that 4 conserved chondrocyte subpopulations and 1 conserved mesenchymal population were observed in C59-treated pellets**

(A) Jitter plots showing that C59-treated pellets had increased expression of *ACAN, COL2A1,* and *SOX9* but decreased *SOX2* versus Standard TGF-β3-treated pellets
(B) Temporal expression profiles of signature genes of each chondrocyte subpopulation. *CDK1* and *IGFBP5* showed transient upregulation while *COL9A1* and *COL11A1* remained up-regulated once activated. *MMP13* and *MX1* showed increased expression levels at later time points.
(C) Dynamic changes in the percentage of the cell population within the pellets over the course of differentiation.
(D) *ISG15/IFI6/MX1*+ chondrocytes contained 4.6% cells expressing both *VEGF*A and *MMP13*
(E-F) *BMPR1B/ITGA4*+ progenitors previously identified in articular cartilage were mostly observed in *HMGB2/CDK1*+ proliferating chondrocytes.
(G) *LECT1/EPYC/FRZB*+ early-mature chondrocytes had the highest levels of *COL2A1* and *ACAN* expression among other chondrocyte subpopulations.
(H) *ISG15/IFI6/MX1*+ mature-hypertrophic chondrocytes expressed several IFN-related genes.
(I) In comparison with *IGFBP5*+ early chondrocytes, *ISG15/IFI6/MX1*+ mature-hypertrophic chondrocytes showed high expression in *IGFBP3* but decreased expression in *FOS*.
(J) The expression of various hypertrophic chondrocyte markers.
For scRNA-seq analysis of C59 treated pellets, total 7,997 cells (from d7-d42) passed quality control and thus were analyzed for this figure.
(E)-(F) A total of 7,977 cells from d7-d42 timepoints of C59-treated pellets was used to performed CCA alignment.

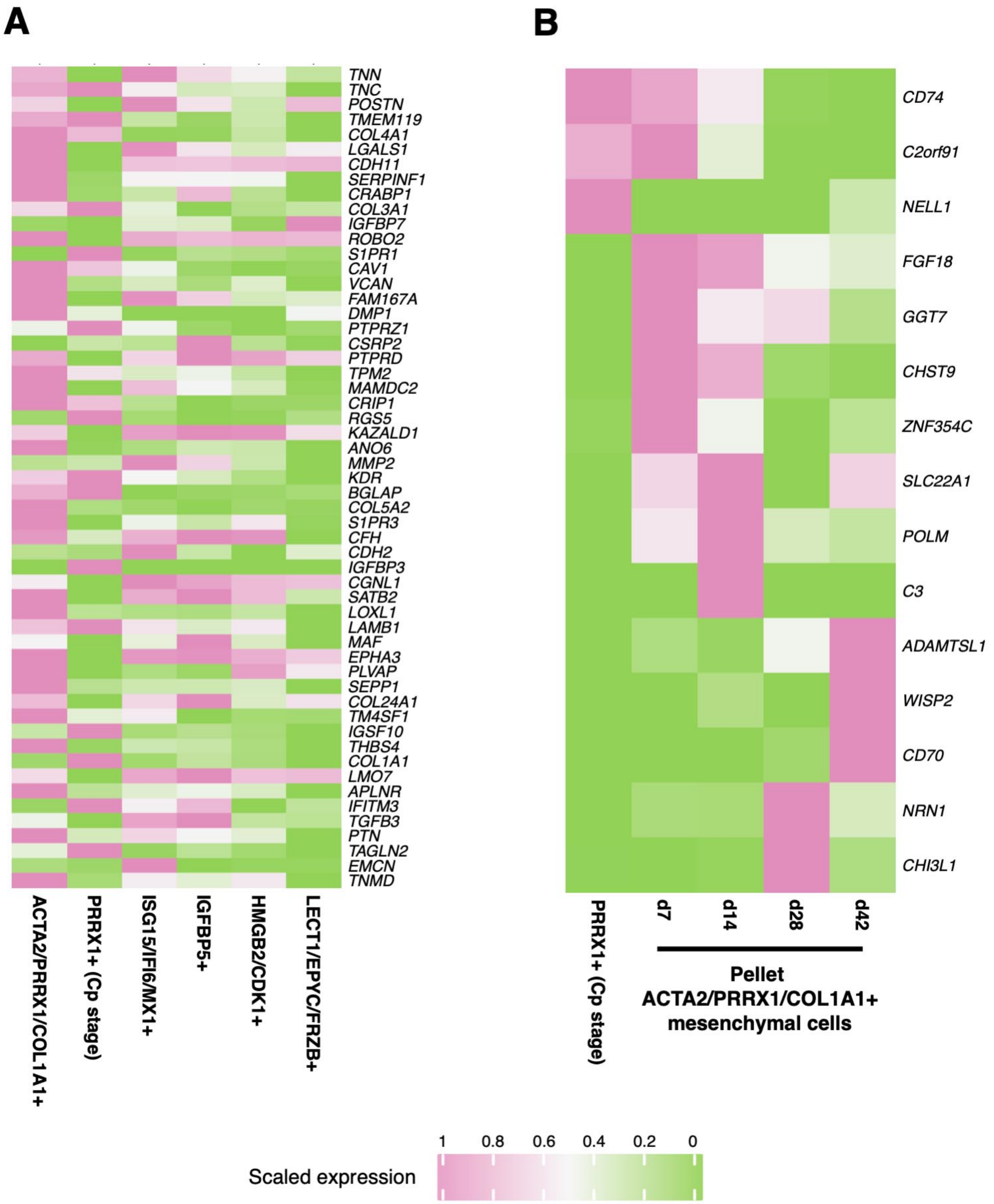


**Supplementary Figure 7. *ACTA2/PRRX1/COL1A1+* mesenchymal cells in the pellets, but not mesenchymal cells at the Cp stage, exhibit similar gene expression profile to perichondrial cells.**
(A) *ACTA2/PRRX1/COL1A1+* mesenchymal cells in the pellets expressed markers of rat perichondrial cells.
(B) *ACTA2/PRRX1/COL1A1+* mesenchymal cells from d7 and d14 pellets were enriched with 8 of 15 differentially expressed genes in the perichondrium-like membrane of the human chondrogenic pellet. Particularly, d7 *ACTA2/PRRX1/COL1A1+* mesenchymal cells had the highest expression of *C2orf91*, *FGF18*, *GGT7*, *CHST9*, and *ZNH354C.* Interestingly, we also observed that there was gradual shift in the gene expression profile of *ACTA2/PRRX1/COL1A1+* mesenchymal cells from d28 to d42. For example, d28 *ACTA2/PRRX1/COL1A1+* mesenchymal cells were enriched in *NRN1* and *CH3L1* while d42 cells had the highest expression of *ADAMTSL1*, *WISP2*, and *CD70*.

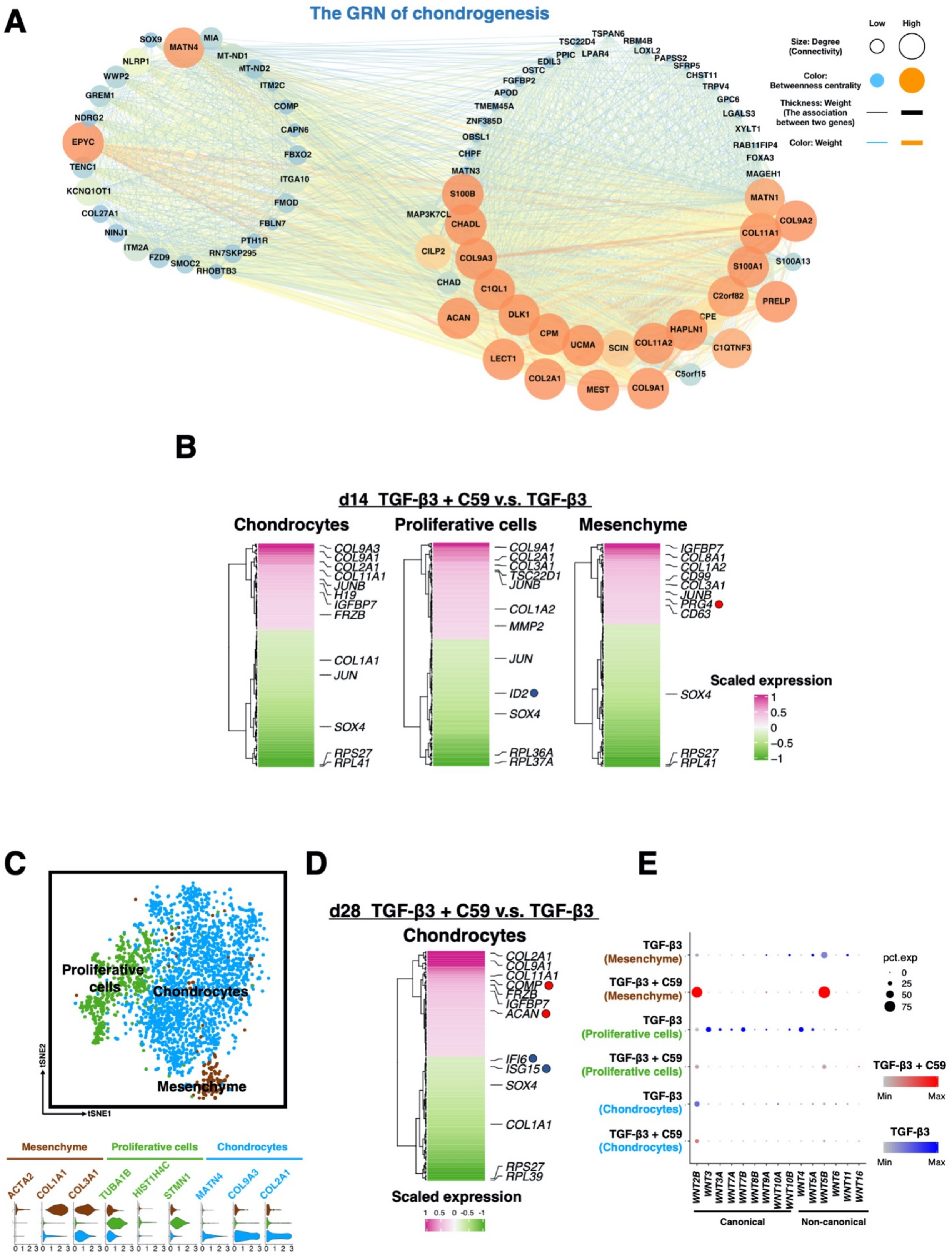


**Supplementary Figure 8. The GRN of hiPSC chondrogenesis**
(A) The GRN and hub genes of hiPSC chondrogenesis.

(B) CCA was used to identify DEGs of each subpopulation between d14 pellets with and without C59 treatment. *ID2*, a neurogenic marker (blue circle), was decreased in proliferative cells in C59-treated pellets, while *PRG4* (red circle) was increased in mesenchymal cells in C59-treated pellets.
(C) CCA alignment of cells from d28 pellets with and without C59 treatment. A total of 3,027 cells from d28 pellets with and without C59 treatment was used to performed CCA alignment.
(D) CCA was used to identify DEGs of chondrocytes between d28 pellets with and without C59 treatment. Markers for mature-hypertrophic chondroocytes, such as *IFI6* and *ISG15* (blue circles), were decreased while *ACAN* and *COMP* (red circles) were increased in C59-treated pellets.
(E) Similar to the WNT expression profiles in d14 pellets, most WNTs were expressed by proliferative cells in the d28 pellets treated with TGF-β3.

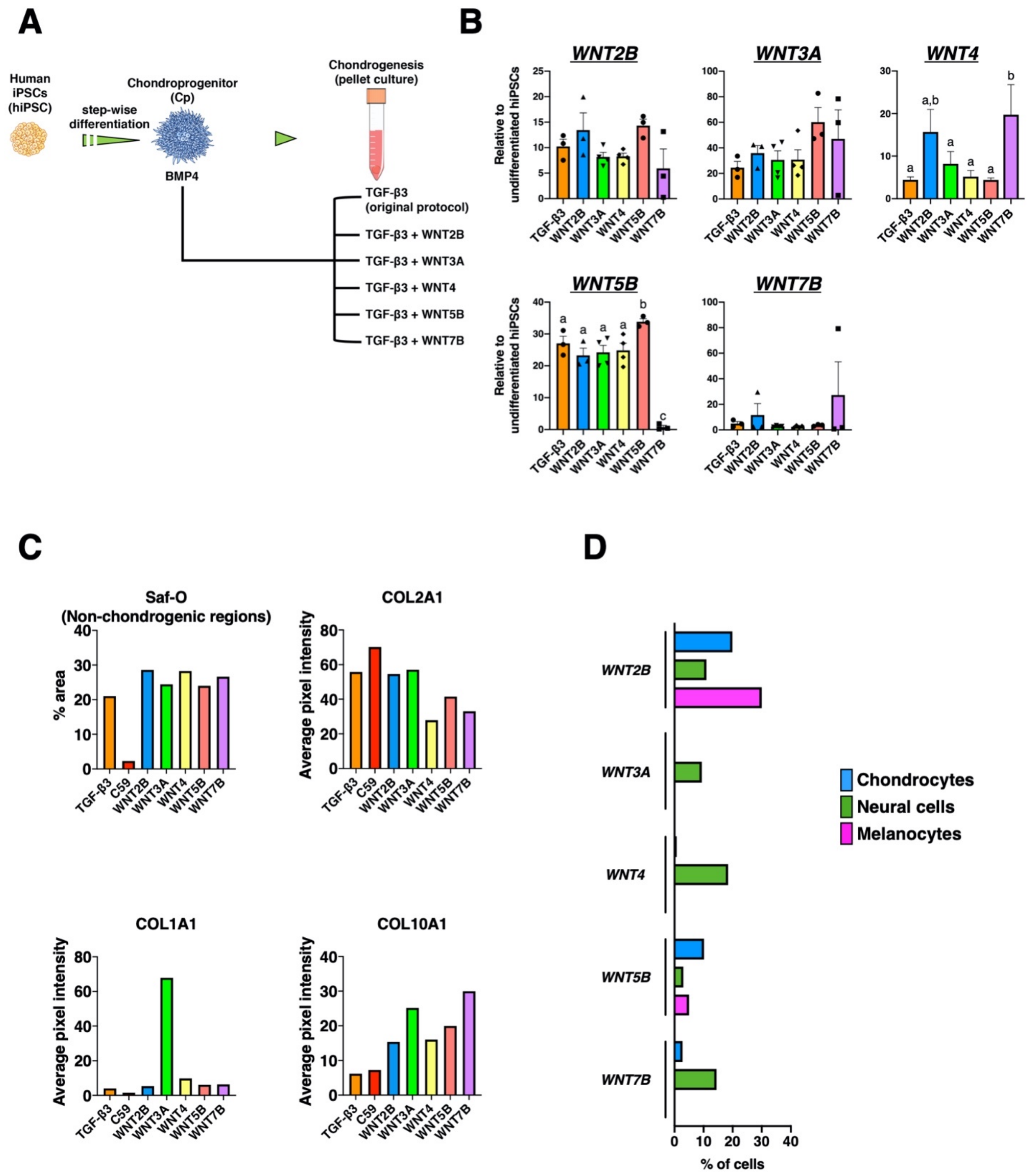


**Supplementary Figure 9.**
(A) Schematic of WNT treatment during chondrogenic pellet culture.
(B) RT-qPCR of d14 pellets treated various WNTs showing that gene expression of WNTs can be modulated by other WNT ligands. Different letters are significantly different from each other ($p < 0.05$). Mean ± SEM. n = 3-4 pellets per group. Statistical significance was determined by one-way ANOVA with Tukey's *post-hoc* test.
(C) Semi-quantification of Saf-O and IHC labeling against various collagens.

(D) Percentage of the cells expressing a variety of WNTs in d14 pellets treated with TGF-β3. For scRNA-seq analysis of d14 TGF-β3 treated pellets, total 2,148 cells passed quality control and thus were analyzed.

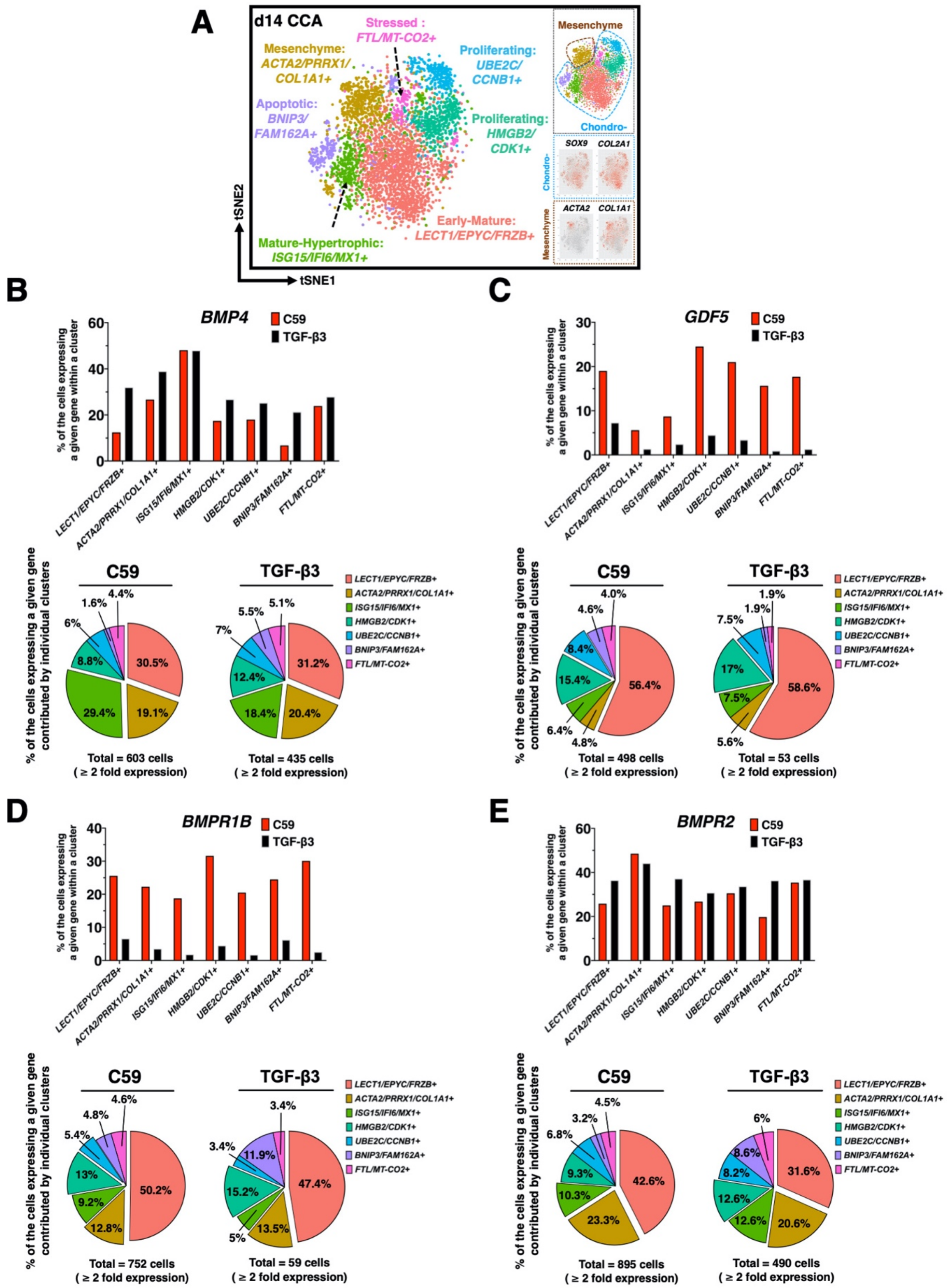


**Supplementary Figure 10. Differential expression of BMPs/GDFs and receptors in response to WNT inhibition**

(A) CCA alignment of chondrocyte and mesenchymal populations from TGF-β3 only and C59 conditions.

(B) C59-treated pellets had a decreased percentage of *BMP4* expressing cells within all clusters except within *ISG15/IFI6/MX1*+ mature-hypertrophic chondrocytes.
(C and D) C59-treated pellets demonstrated a remarkably increased percentage of *GDF5* and *BMPR1B* expressing cells within all clusters versus TGF-β3-treated pellets.
(E) C59 treatment decreased percentage of cells expressing *BMPR2* in *LECT1/EPYC/FRZB*+ early-mature chondrocytes*, ISG15/IFI6/MX1*+ mature-hypertrophic chondrocytes, *BJIP3/FAM162*+ apoptotic chondrocytes, as well as *HMGB2/CDK1*+ and *UBE2C/CCNB1/KPNA2*+ proliferating chondrocytes.
(B-E) Note that C59 treatment did not significantly affect the contribution of a cluster to the cells expressing *BMP4*, *GDF5, BMPR1B,* and *BMPR2* as presented in the pie charts.
For bioinformatic analysis, CCA was performed with a total of 1,335 cells from mesenchymal and chondrocyte populations from d14 TGF-β3 pellets and with a total of 3,047 cells from mesenchymal and chondrocyte populations from d14 C59 pellets.

**A**

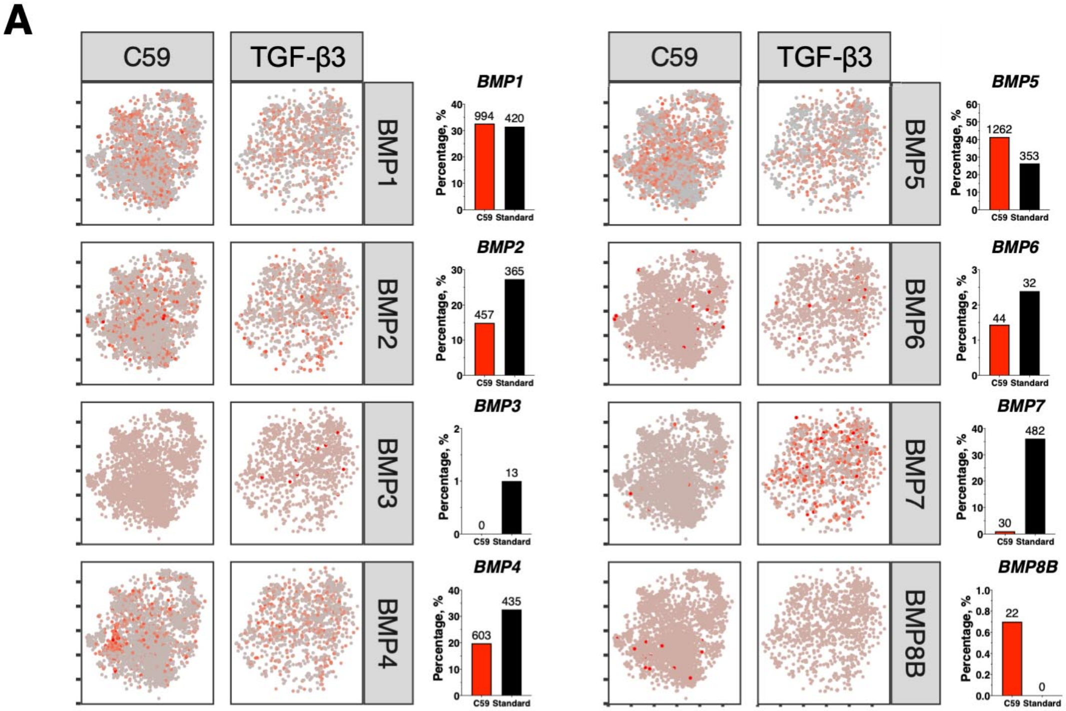


**B**

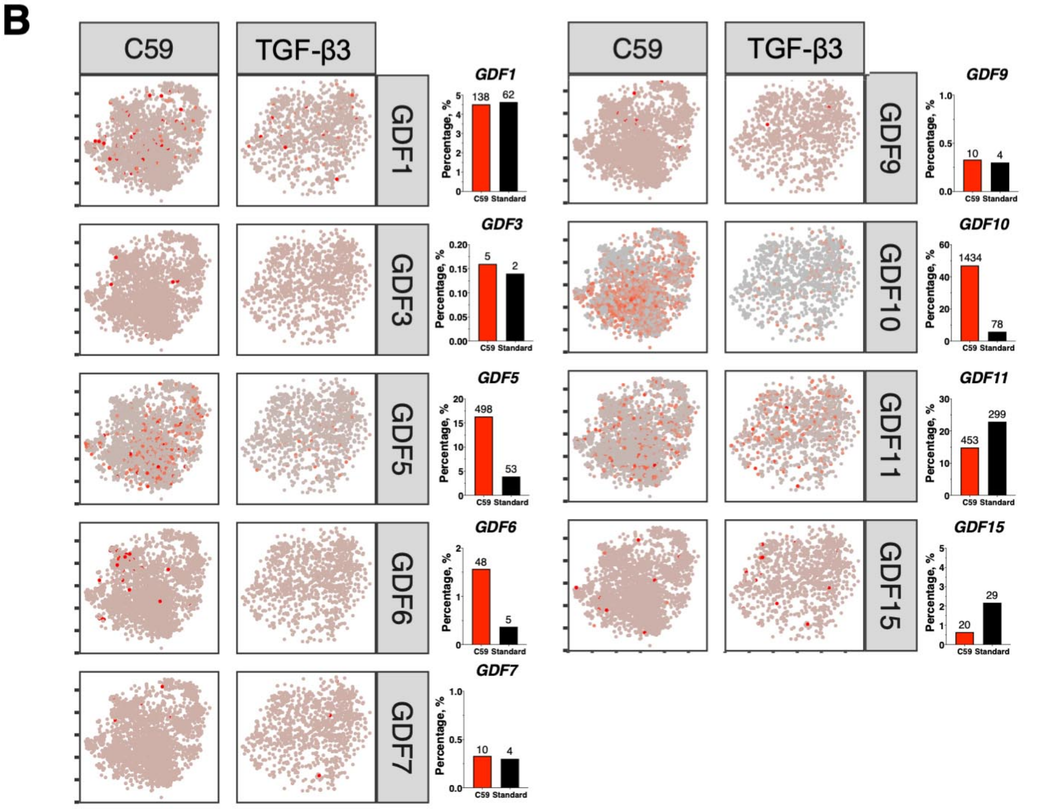


**Supplementary Figure 11. CCA analysis showing differential gene expression with C59 treatment.**

(A) BMP and (B) GDF families in chondrocyte subpopulations due to C59 treatment. Numerical value on top of each bar in the bar graph indicates cell numbers expressing a given gene. For bioinformatic analysis, CCA was performed with a total of 1,335 cells from mesenchymal and chondrocyte populations from d14 TGF-β3 pellets and with a total of 3,047 cells from mesenchymal and chondrocyte populations from d14 C59 pellets

**A**

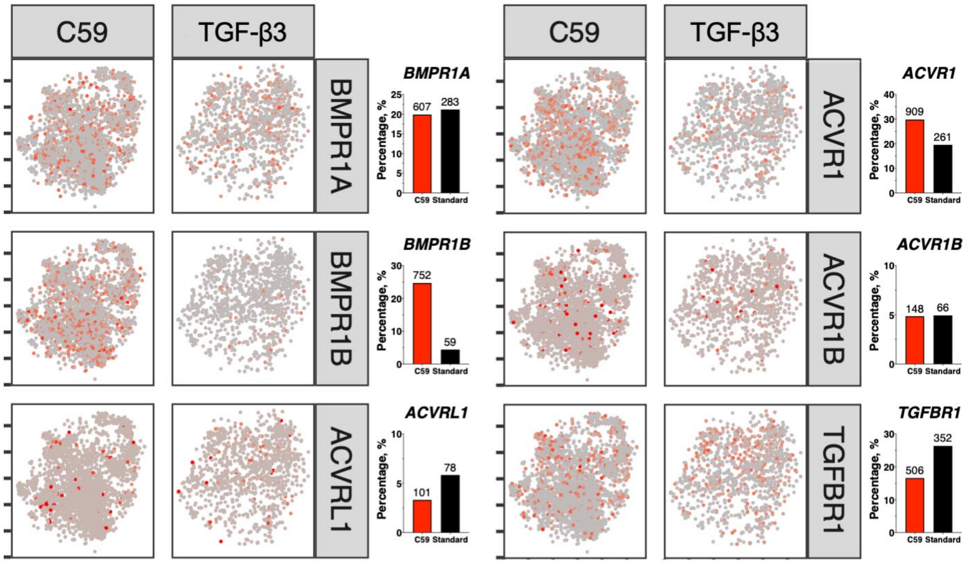


**B**

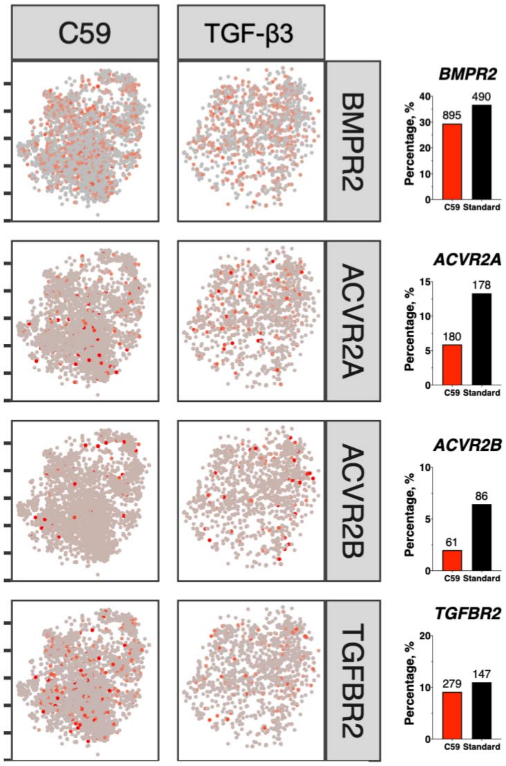


**Supplementary Figure 12. CCA analysis showing differential receptor gene expression with C59 treatment.**

(A) Type I and (B) type II receptors for the BMP/GDF family in chondrocyte subpopulations due to C59 treatment. Numerical value on top of each bar in the bar graph indicates cell numbers expressing a given gene. For bioinformatic analysis, total 2,148 cells from d14 TGF-β3 treated pellets and total 3,076 cells from d14 C59+TGF-β3 treated pellets passed quality control and thus were analyzed for this figure.

**Supplementary Table 1. Related to Figure 1. Top 10 up-regulated genes in fold change in mesodermal phase**

| Ensembl | Symbol | AP vs hiPSC | PM vs AP | ES vs Par | Scl vs ES | ES vs Cp |
|---|---|---|---|---|---|---|
| ENSG00000113722 | ***CDX1*** | **7.47** | 1.28 | -5.77 | -1.90 | -2.03 |
| ENSG00000185155 | ***MIXL1*** | **7.32** | -2.67 | -4.09 | -2.29 | 0.91 |
| ENSG00000222033 | ***LINC01124*** | **7.32** | 0.31 | -5.65 | -2.48 | 2.72 |
| ENSG00000164458 | ***T*** | **7.19** | -0.13 | -5.22 | -2.84 | -1.81 |
| ENSG00000104371 | ***DKK4*** | **7.13** | -2.61 | -5.28 | -0.92 | 1.07 |
| ENSG00000274981 | | **6.99** | -0.44 | -4.38 | -2.01 | -0.10 |
| ENSG00000241345 | ***LOC105375483*** | **6.80** | 1.09 | -5.53 | -1.25 | 2.06 |
| ENSG00000253308 | | **6.75** | 2.33 | -7.08 | -1.82 | 2.23 |
| ENSG00000106038 | ***EVX1*** | **6.51** | 0.01 | -5.38 | -2.44 | 1.48 |
| ENSG00000105991 | ***HOXA1*** | **6.34** | 3.87 | -2.36 | -1.99 | -1.85 |
| ENSG00000049249 | ***TNFRSF9*** | 1.16 | **6.93** | -5.79 | -0.95 | 3.01 |
| ENSG00000120094 | ***HOXB1*** | 2.25 | **6.54** | -3.53 | -3.39 | -0.37 |
| ENSG00000151379 | ***MSGN1*** | 3.11 | **6.23** | -5.27 | -3.42 | -0.60 |
| ENSG00000137252 | ***HCRTR2*** | 0.01 | **6.01** | -2.13 | -0.74 | 1.57 |
| ENSG00000179111 | ***HES7*** | 0.97 | **5.54** | -2.97 | -2.34 | -0.34 |
| ENSG00000253552 | | 2.07 | **5.13** | 0.68 | -2.19 | -0.52 |
| ENSG00000182742 | ***HOXB4*** | 1.54 | **4.93** | 1.00 | -1.24 | -0.41 |
| ENSG00000129654 | ***FOXJ1*** | -2.73 | **4.73** | -2.53 | 1.02 | 1.48 |
| ENSG00000163083 | ***INHBB*** | -2.68 | **4.55** | -0.68 | -2.63 | 1.58 |
| ENSG00000100678 | ***SLC8A3*** | -0.89 | **4.53** | -0.27 | -0.63 | -1.42 |
| ENSG00000147223 | ***RIPPLY1*** | -0.02 | -0.99 | **11.49** | -8.93 | -1.53 |
| ENSG00000005102 | ***MEOX1*** | 0.06 | 0.37 | **9.05** | -1.35 | -5.05 |
| ENSG00000164853 | ***UNCX*** | -1.66 | 0.00 | **8.92** | -0.59 | -1.21 |
| ENSG00000136698 | ***CFC1*** | -2.67 | 2.82 | **8.33** | -4.80 | -1.36 |
| ENSG00000224865 | ***LOC101928782*** | 0.07 | 0.01 | **8.17** | -2.92 | -4.34 |
| ENSG00000168269 | ***FOXI1*** | 1.00 | -0.91 | **8.12** | -4.89 | -3.26 |
| ENSG00000102837 | ***OLFM4*** | 0.99 | -0.16 | **7.77** | -4.75 | -0.94 |
| ENSG00000176692 | ***FOXC2*** | 0.89 | 0.83 | **7.24** | -1.11 | -2.23 |
| ENSG00000176678 | ***FOXL1*** | -0.32 | 0.94 | **7.15** | -2.86 | -1.48 |
| ENSG00000213931 | ***HBE1*** | 1.07 | 0.33 | **7.12** | -1.58 | -0.82 |
| ENSG00000136327 | ***NKX2-8*** | 2.46 | -0.32 | -2.04 | **9.99** | -3.90 |
| ENSG00000184302 | ***SIX6*** | 0.22 | -1.31 | -3.65 | **8.29** | -0.28 |
| ENSG00000008196 | ***TFAP2B*** | 2.42 | 0.63 | -4.51 | **7.30** | 1.47 |
| ENSG00000274021 | | -0.86 | 0.85 | -6.19 | **6.97** | -1.09 |
| ENSG00000064218 | ***DMRT3*** | 0.99 | 0.80 | -2.70 | **6.97** | -3.17 |
| ENSG00000139318 | ***DUSP6*** | -0.39 | 0.72 | -6.78 | **6.96** | -1.03 |
| ENSG00000176165 | ***FOXG1*** | 0.14 | 0.67 | -0.64 | **6.81** | -3.11 |
| ENSG00000244405 | ***ETV5*** | -0.05 | 0.62 | -6.23 | **6.76** | -1.15 |
| ENSG00000152785 | ***BMP3*** | -0.82 | 0.49 | 0.16 | **6.63** | -3.57 |
| ENSG00000178235 | ***SLITRK1*** | -0.99 | 0.33 | -2.34 | **6.62** | -2.86 |
| ENSG00000180828 | ***BHLHE22*** | 2.88 | -1.47 | -2.00 | -3.08 | **7.73** |
| ENSG00000167941 | ***SOST*** | -1.11 | 0.36 | 5.37 | -4.87 | **7.09** |
| ENSG00000188620 | ***HMX3*** | -2.25 | -0.08 | 2.35 | -3.91 | **6.47** |
| ENSG00000164125 | ***FAM198B*** | 0.87 | -0.59 | -0.25 | 1.80 | **6.30** |
| ENSG00000163132 | ***MSX1*** | 4.90 | 0.75 | -2.60 | -1.44 | **6.30** |
| ENSG00000175899 | ***A2M*** | -0.10 | -0.72 | -0.41 | 1.55 | **6.29** |
| ENSG00000165092 | ***ALDH1A1*** | -0.27 | -1.01 | -1.47 | 0.82 | **6.20** |
| ENSG00000109846 | ***CRYAB*** | -1.24 | -0.25 | -0.09 | -0.25 | **6.12** |
| ENSG00000107984 | ***DKK1*** | 5.15 | -0.85 | -3.78 | -2.19 | **6.10** |
| ENSG00000122641 | ***INHBA*** | -1.45 | -1.94 | 1.50 | -0.96 | **6.07** |

**AP**: anterior primitive streak; **PM**: paraxial mesoderm; **ES**: early somite, **Scl**: Sclerotome; **Cp**: Chondroprogenitor

**Supplementary Table 2. Related to Figure 1. Top 10 up-regulated genes in fold change in chondrogenic phase**

| Ensembl | Symbol | d7 vs Cp | d14 vs d7 | d21 vs d14 | d28 vs d21 | d42 vs d28 |
|---|---|---|---|---|---|---|
| ENSG00000124159 | ***MATN4*** | **7.69** | 1.22 | 0.73 | -1.28 | -0.68 |
| ENSG00000181195 | ***PENK*** | **7.65** | 1.16 | -0.23 | -0.15 | -0.51 |
| ENSG00000008441 | ***NFIX*** | **7.08** | -0.36 | 1.15 | -0.69 | 0.43 |
| ENSG00000006611 | ***USH1C*** | **7.01** | -0.34 | -0.37 | -0.64 | 0.73 |
| ENSG00000157554 | ***ERG*** | **6.44** | -0.47 | -0.45 | -0.25 | -0.51 |
| ENSG00000171812 | ***COL8A2*** | **6.19** | -0.01 | 0.68 | -1.48 | 0.21 |
| ENSG00000124134 | ***KCNS1*** | **6.18** | 0.48 | 0.42 | -0.68 | -1.88 |
| ENSG00000121898 | ***CPXM2*** | **5.87** | 0.80 | 1.01 | -0.05 | 0.52 |
| ENSG00000224765 | | **5.75** | -0.37 | 0.53 | -0.83 | -0.15 |
| ENSG00000145708 | ***CRHBP*** | **5.66** | -2.59 | 0.34 | 0.56 | -3.25 |
| ENSG00000249945 | | -2.48 | **5.07** | -4.36 | -0.50 | 0.13 |
| ENSG00000135480 | ***KRT7*** | -2.54 | **4.98** | 1.00 | -0.08 | -0.17 |
| ENSG00000278530 | ***CHMP1B2P*** | -0.11 | **4.80** | -4.40 | -1.15 | -1.39 |
| ENSG00000234787 | ***LINC00458*** | -2.38 | **4.34** | -2.06 | 0.93 | -0.91 |
| ENSG00000182798 | ***MAGEB17*** | -3.16 | **4.24** | -1.13 | -1.02 | 2.03 |
| ENSG00000278840 | | -2.23 | **4.19** | 0.70 | -0.59 | 0.73 |
| ENSG00000253507 | | -0.90 | **4.00** | -2.28 | -0.13 | 0.48 |
| ENSG00000187569 | ***DPPA3*** | 0.15 | **3.96** | -1.73 | 0.95 | -0.79 |
| ENSG00000196767 | ***POU3F4*** | 2.51 | **3.94** | -1.98 | -0.60 | -2.49 |
| ENSG00000101842 | ***VSIG1*** | -1.03 | **3.81** | -1.35 | -0.37 | 2.15 |
| ENSG00000011083 | ***SLC6A7*** | -0.34 | -1.09 | **6.22** | -1.47 | -0.62 |
| ENSG00000205890 | ***LOC100128770*** | -2.25 | 1.60 | **5.78** | -3.33 | -1.20 |
| ENSG00000152213 | ***ARL11*** | -1.54 | -1.42 | **5.10** | -4.03 | 1.23 |
| ENSG00000233841 | ***HLA-C*** | -0.97 | 0.94 | **5.06** | -5.06 | 1.81 |
| ENSG00000117091 | ***CD48*** | 0.74 | -0.73 | **4.77** | -3.40 | -1.04 |
| ENSG00000166869 | ***CHP2*** | -3.55 | 0.82 | **4.70** | -4.51 | 1.21 |
| ENSG00000204121 | | -3.93 | 1.36 | **4.51** | -3.57 | 1.80 |
| ENSG00000224865 | ***LOC101928782*** | 0.13 | -0.54 | **4.48** | -4.29 | 2.13 |
| ENSG00000182912 | | -0.87 | -1.29 | **4.45** | -2.86 | 0.11 |
| ENSG00000188257 | ***PLA2G2A*** | 2.84 | -1.28 | **4.22** | 1.86 | -0.02 |
| ENSG00000213931 | ***HBE1*** | -3.49 | 1.31 | -0.55 | **3.12** | -4.81 |
| ENSG00000207927 | ***MIR302A*** | -0.72 | 0.80 | -1.84 | **2.87** | 0.72 |
| ENSG00000120094 | ***HOXB1*** | -0.17 | 1.40 | -1.90 | **2.82** | -3.71 |
| ENSG00000164746 | ***C7orf57*** | -0.30 | -0.16 | -0.92 | **2.77** | -0.75 |
| ENSG00000183463 | ***URAD*** | -2.49 | -0.82 | 0.69 | **2.68** | -3.05 |
| ENSG00000203635 | | -0.46 | 0.86 | -2.09 | **2.66** | -1.49 |
| ENSG00000131095 | ***GFAP*** | 0.18 | 0.65 | 1.11 | **2.65** | 0.93 |
| ENSG00000255282 | ***WTAPP1*** | -2.90 | -1.23 | 0.75 | **2.62** | -0.60 |
| ENSG00000101276 | ***SLC52A3*** | -1.43 | 1.12 | -1.39 | **2.57** | -0.83 |
| ENSG00000277060 | ***NLRP2*** | -1.14 | 1.39 | -1.19 | **2.56** | -2.86 |
| ENSG00000166828 | ***SCNN1G*** | -2.23 | 0.06 | -0.22 | 0.46 | **4.32** |
| ENSG00000129451 | ***KLK10*** | -2.31 | -0.66 | 1.84 | 0.73 | **3.96** |
| ENSG00000168447 | ***SCNN1B*** | -1.71 | 0.49 | 3.90 | -3.56 | **3.84** |
| ENSG00000176654 | | -1.05 | 2.23 | 1.68 | -4.23 | **3.50** |
| ENSG00000234745 | ***HLA-B*** | 0.48 | -0.20 | 0.68 | -1.09 | **3.48** |
| ENSG00000013588 | ***GPRC5A*** | 0.83 | -0.05 | 1.38 | 0.16 | **3.45** |
| ENSG00000105392 | ***CRX*** | 0.14 | 1.54 | -1.45 | -1.03 | **3.32** |
| ENSG00000137265 | ***IRF4*** | -1.02 | -0.43 | 3.20 | -1.55 | **3.27** |
| ENSG00000123689 | ***G0S2*** | -2.25 | 0.07 | 1.75 | 0.99 | **3.25** |
| ENSG00000161652 | ***IZUMO2*** | -1.81 | -1.56 | 0.78 | 0.27 | **3.02** |

**Supplementary Table 3. Key resources**

| REAGENT or RESOURCE | SOURCE | IDENTIFIER |
|---|---|---|
| **Antibodies** | | |
| COL1A1 | Abcam | Cat#90395 |
| COL2A1 | Iowa Hybridoma Bank | Cat#II-II6B3-s |
| COL6A1 | Fitzgerald Industries | Cat#70F-CR009X |
| COL10A1 | Sigma | Cat#C7974 |
| Goat Anti-Mouse | Abcam | Cat#97021 |
| Goat Anti-Rabbit | Abcam | Cat#6720 |
| Probe- Hs-WNT3A | Advanced Cell Diagnostics | Cat#429431 |
| Probe- Hs-COL2A1 | Advanced Cell Diagnostics | Cat#427871 |
| Probe- Hs-WNT4 | Advanced Cell Diagnostics | Cat#429441 |
| Anti-Nestin antibody | Abcam | Cat#ab105389 |
| Anti-Wnt2B | Abcam | Cat#ab178418 |
| Anti-Wnt5B | Abcam | Cat#ab93134 |
| Anti-Wnt3A | Abcam | Cat#ab81614 |
| Anti-Wnt4 | Abcam | Cat#ab91226 |
| Anti-Wnt7B | Abcam | Cat#b155313 |
| FITC-CD45 | Biolegend | Cat#304006 |
| PE/Cy7-CD146 | Biolegend | Cat#361008 |
| PE-CD166 | Biolegend | Cat#343904 |
| Tru Stain FC X™ | Biolegend | Cat#422302 |
| DAPI | Biolegend | Cat#422801 |
| **Chemicals, Peptides, and Recombinant Proteins** | | |
| Activin | R&D Systems | Cat#338-AC |
| CHIR99021 | Stemgent | Cat#04-0004 |
| FGF2 | R&D Systems | Cat#233-FB-025/CF |
| Dorsomorphin | Stemgent | Cat#04-0024 |
| BMP4 | R&D Systems | Cat#314-BP-010/CF |
| Wnt-C59 | Cellagen Technology | Cat#C7641-2s |
| ML329 | Axon Medchem | CAT#HY-101464 |
| SB-505124 | Tocris | Cat#3263 |
| PD173074 | Tocris | Cat#3044 |
| Purmorphamine | Stemgent | Cat#04-0009 |
| TGF-β3 | R&D Systems | Cat#243-B3-010/CF |
| RNAscope® Fluorescent Multiplex Reagent Kit | Advanced Cell Diagnostics | Cat#320850 |
| RNAscope® Wash Buffer Reagents | Advanced Cell Diagnostics | Cat#310091 |
| Vitronectin | Thermo Fisher Scientific | Cat#A31804 |
| ReLeSR | STEMCELL Technologies | Cat#05872 |

| | | |
|---|---|---|
| Collagenase, Type 2 | Worthington Biochemical | Cat#LS004177 |
| Papain | Sigma–Aldrich | Cat#P4762 |
| Human BMP-4 | R&D Systems | Cat#314-BP-01M |
| Human WNT-4 | R&D Systems | Cat#6076-WN-005 |
| Human WNT-3A | R&D Systems | Cat#5036-WN-010 |
| Mouse WNT-2B | R&D Systems | Cat#3900-WN-025 |
| Human WNT-5B | R&D Systems | Cat#7347-WN-025 |
| Human WNT7B | Abcam | Cat#ab152805 |
| Essential 8 Flex medium | Thermo Fisher Scientific | Cat#A2858501 |
| CellMatrix Basement Membrane | ATCC | Cat#ACS3035 |
| Pluripotent Stem Cell SFM XF/FF medium | ATCC | Cat#ACS3002 |
| ReLeSR | STEMCELL Technologies | Cat#05872 |
| DMEM-low glucose | Thermo Fisher Scientific | Cat#11885092 |
| Penicillin/streptomycin | Thermo Fisher Scientific | Cat#15140-122 |
| Fetal bovine serum | Atlanta Biologicals | Cat#S11550 |
| Fibroblast growth factor | R&D Systems | Cat#233-FB |
| Ham's F-12 | Thermo Fisher Scientific | Cat#31765092 |
| IMDM | Thermo Fisher Scientific | Cat#31980097 |
| DMEM/F-12, | Thermo Fisher Scientific | Cat#10565042 |
| Y-27632 | STEMCELL Technologies | Cat#72304 |
| chemically defined lipid concentrates | Gibco | Cat#11905031 |
| ITS+ | Corning | Cat#354352 |
| 1-thioglycerol | Sigma–Aldrich | Cat#M6145 |
| TrypLE | Gibco | Cat#12604013 |
| β-mercaptoethanol | Thermo Fisher Scientific | Cat#21985023 |
| dexamethasone | Sigma–Aldrich | Cat#D4902 |
| L-proline | Sigma–Aldrich | Cat#P5607 |
| L-ascorbic acid | Sigma–Aldrich | Cat#A8960 |
| NEAA | Gibco | Cat#11140050 |
| **Critical Commercial Assays** | | |
| Total RNA Purification Kit | Norgen Biotek | Cat#37500 |
| Ribo-Zero Gold rRNA Removal kit | illumina | https://www.illumina.com/products/selection-tools/rrna-depletion-selection-guide.html |
| Single Cell 30 Library and Gel Bead Kit V2 | 10x Genomics | Cat#120237 |
| Chromium single cell chip kit V2 | 10x Genomics | Cat#120236 |

| | | |
|---|---|---|
| Agilent High Sensitivity DNA Kit | Agilent | Cat#5067-4626 |
| Quant-iT PicoGreen dsDNA Assya Kit | Thermo Fisher Scientific | Cat#P11496 |
| **Software and Algorithms** | | |
| R v3.5 | R project | https://www.r-project.org |
| Cell Ranger v3 | 10x Genomics | https://support.10xgenomics.com/ |
| DESeq2 | (Love et al., 2014)[47] | https://bioconductor.org/packages/release/bioc/html/DESeq2.html |
| Seurat v2.4 | (Butler et al., 2018)[15] | https://satijalab.org/seurat/ |
| Monocle2 | (Qiu et al., 2017)[62] | https://github.com/cole-trapnell-lab/monocle-release |
| WGCNA | (Langfelder et al., 2008)[20] | https://cran.r-project.org/web/packages/WGCNA/index.html |
| Circlize | (Gu et al., 2014)[48] | https://github.com/jokergoo/circlize |
| Cytoscape 3.6.1 | (Shannon et al., 2003)[49] | https://www.cytoscape.org/ |
| ComplexHeatmap | (Gu et al., 2016)[50] | https://www.bioconductor.org/packages/release/bioc/html/ComplexHeatmap.html |
| **Other** | | |
| Polysorb™, suture | Covidien | Cat#L-2800 |
| Micro bone drill | Roboz | Cat#RS-6300A |
| Epinephrine 1:1000 | International Medication Systems | Cat#491590 |
| Zirconia beads | BioSpec Products | Cat#11079110zx |

**Supplementary Table 4. Subject details**

| Donor ID | Study ID | Cell type | GenoType | Age, Sex | Characterization |
|---|---|---|---|---|---|
| PHBI-BA-060 | STAN | iPSC | wt/wt | 1 yr, M | Ref[8] |
| SCRC-1041 | ATCC | iPSC | wt/wt | Newborn, M | Ref[48] |
| BJFF.6 | BJFF | iPSC | wt/wt | Newborn, M | Ref[49] |
| hMSC3 | Donor 1 | MSC | wt/wt | 51, F | This study |
| hMSC4 | Donor 2 | MSC | wt/wt | 38, M | This study |
| hMSC6 | Donor 3 | MSC | wt/wt | 26, F | This study |

**Supplementary Table 5. Numbers of the cells passed quality control (QC) for each stage and associated highly variable genes**

| | Detected cells | Cells passed QC | Median genes/cell | Highly variable genes |
|---|---|---|---|---|
| **Monolayer culture** | | | | |
| hiPSC | 6258 | 4798 | 1688 | 1651 |
| Sclerotome | 2237 | 1861 | 3107 | 2240 |
| Cp | 2536 | 1888 | 3443 | 1754 |
| **Sum** | 11031 | 8547 | 8238 | 5645 |
| **Average** | 3677 | 2849 | 2746 | 1882 |
| **TGF-β3 chondrogenic pellets** | | | | |
| d1 | 2418 | 2216 | 3447 | 2097 |
| d3 | 2810 | 2485 | 3357 | 1835 |
| d7 | 1369 | 1200 | 4049 | 2302 |
| d14 | 2266 | 2148 | 3784 | 2001 |
| d28 | 1321 | 1271 | 2900 | 2178 |
| d42 | 1355 | 1328 | 2324 | 1955 |
| **Sum** | 11539 | 10648 | 19861 | 12368 |
| **Average** | 1923 | 1775 | 3310 | 2061 |
| **TGF-β3 + C59 chondrogenic pellets** | | | | |
| d7 | 2191 | 1682 | 4030 | 1733 |
| d14 | 3461 | 3076 | 2718 | 1693 |
| d28 | 1881 | 1756 | 2714 | 1991 |
| d42 | 1926 | 1483 | 3003 | 2126 |
| **Sum** | 9459 | 7997 | 12456 | 7543 |
| **Average** | 2365 | 1999 | 3116 | 1886 |

**Supplementary Table 6: qRT-PCR Primer Sequences.** Primers are listed as 5' to 3'.

| Gene | Forward Sequence | Reverse Sequence |
|---|---|---|
| *ACAN* | CACTTCTGAGTTCGTGGAGG | ACTGGACTCAAAAAGCTGGG |
| *COL1A1* | TGTTCAGCTTTGTGGACCTC | TTCTGTACGCAGGTGATTGG |
| *COL2A1* | GGCAATAGCAGGTTCACGTA | CTCGATAACAGTCTTGCCCC |
| *COL10A1* | CATAAAAGGCCCACTACCCAAC | ACCTTGCTCTCCTCTTACTGC |
| *MITF* | AGTTGCTGGCGTAGCAAGAT | AAAGTCAACCGCTGAAGAGC |
| *PAX6* | GAGTGCCCGTCCATCTTTG | GTCTGCGCCCATCTGTTGCTTTTC |
| *SOX2* | TACAGCATGATGCAGGACCA | CCGTTCATGTAGGTCTGCGA |
| *SOX9* | CGTCAACGGCTCCAGCAAGAACAA | GCCGCTTCTCGCTCTCGTTCAGAAGT |
| *TBP* | AACCACGGCACTGATTTTCA | ACAGCTCCCCACCATATTCT |
| *WNT2B* | GTGTCCTGGCTGGTTCCTTA | AGCTGGTGCAAAGGAAAGAA |
| *WNT3A* | CCTGCACTCCATCCAGCTACA | GACCTCTCTTCCTACCTTTCCCTTA |
| *WNT4* | GATGTGCGGGAGAGAAGCAA | ATTCCACCCGCATGTGTGT |
| *WNT5B* | CTGCCTTTCCAGCGAGAATT | AGGTCAAATGGCCCCCTTT |
| *WNT7B* | CCCCCTCCCTGGATCATGCACA | GCCACCACGGATGACAGTGCT |
