# Supplementary material for: Single cell transcriptomic analysis of human pluripotent stem cell chondrogenesis
Source: Nat Commun. 2021 Jan 13;12:362. doi: 10.1038/s41467-020-20598-y (PMC7806634; doi:10.1038/s41467-020-20598-y)
Supplement: Supplementary file 2 — Reporting Summary [file 41467_2020_20598_MOESM2_ESM.pdf]

nature research

Corresponding author(s): Farshid Guilak

Last updated by author(s): Oct 20, 2020

Reporting Summary

Nature Research wishes to improve the reproducibility of the work that we publish. This form provides structure for consistency and transparency in reporting. For further information on Nature Research policies, see our Editorial Policies and the Editorial Policy Checklist.

Statistics

For all statistical analyses, confirm that the following items are present in the figure legend, table legend, main text, or Methods section.

n/a	Confirmed	
[ ]	[x]	The exact sample size (*n*) for each experimental group/condition, given as a discrete number and unit of measurement
[ ]	[x]	A statement on whether measurements were taken from distinct samples or whether the same sample was measured repeatedly
[ ]	[x]	The statistical test(s) used AND whether they are one- or two-sided<br>*Only common tests should be described solely by name; describe more complex techniques in the Methods section.*
[ ]	[x]	A description of all covariates tested
[ ]	[x]	A description of any assumptions or corrections, such as tests of normality and adjustment for multiple comparisons
[ ]	[x]	A full description of the statistical parameters including central tendency (e.g. means) or other basic estimates (e.g. regression coefficient) AND variation (e.g. standard deviation) or associated estimates of uncertainty (e.g. confidence intervals)
[ ]	[x]	For null hypothesis testing, the test statistic (e.g. *F*, *t*, *r*) with confidence intervals, effect sizes, degrees of freedom and *P* value noted<br>*Give P values as exact values whenever suitable.*
[x]	[ ]	For Bayesian analysis, information on the choice of priors and Markov chain Monte Carlo settings
[x]	[ ]	For hierarchical and complex designs, identification of the appropriate level for tests and full reporting of outcomes
[ ]	[x]	Estimates of effect sizes (e.g. Cohen's *d*, Pearson's *r*), indicating how they were calculated

Our web collection on statistics for biologists contains articles on many of the points above.

Software and code

Policy information about availability of computer code

Data collection	R version 3.6; Seurat version 2.4; Monocle version 2; WGCNA version 1.68
Data analysis	R version 3.6; Seurat version 2.4; Monocle version 2; SPSS version 25; WGCNA version 1.68; CytoScape version 3.6; FlowJo version 10.5.3.

For manuscripts utilizing custom algorithms or software that are central to the research but not yet described in published literature, software must be made available to editors and reviewers. We strongly encourage code deposition in a community repository (e.g. GitHub). See the Nature Research guidelines for submitting code & software for further information.

Data

Policy information about availability of data

All manuscripts must include a data availability statement. This statement should provide the following information, where applicable:

- Accession codes, unique identifiers, or web links for publicly available datasets
- A list of figures that have associated raw data
- A description of any restrictions on data availability

We acquired RNA-seq datasets of human primary chondrocytes from a previously published study (NIH Gene Expression Omnibus (GEO) accession number GSE106292), in which embryonic hind limb bud chondrocytes (age: 6 weeks, n = 2), adolescent knee chondrocytes (age: 17 weeks, n = 2), adult knee chondrocytes (age: 18-60 years, n = 2), and growth plate chondrocytes (age: 14 weeks, 15 weeks, and 18 weeks, n = 1 per age). For the datasets obtained from the previous mentioned study, gene expression counts were averaged if there were more than 2 samples with the same age. We also harvested chondrocytes from human costal cartilage and performed bulk RNA-seq on these samples (age: ~70 years, n = 3). However, it was challenging to collect rib cartilage from young healthy donors; thus, aged 70-year-old costal cartilages were used. To compare the difference between the phenotypes of chondrocytes derived from hiPSCs and hMSCs, we also used bulk RNA-seq datasets of hMSC chondrogenesis from our recent study (GEO accession number GSE109503). Our bulk RNA-seq and scRNA-seq data are in the process of being uploaded to GEO.

Field-specific reporting

Please select the one below that is the best fit for your research. If you are not sure, read the appropriate sections before making your selection.

- [x] Life sciences
- [ ] Behavioural & social sciences
- [ ] Ecological, evolutionary & environmental sciences

For a reference copy of the document with all sections, see nature.com/documents/nr-reporting-summary-flat.pdf

Life sciences study design

All studies must disclose on these points even when the disclosure is negative.

Sample size	For single cell RNA sequencing (scRNA-seq), sequencing of mixed species was performed prior to the experimental samples to ensure the quality of sequencing with low cell multiplet rate (2.7%) (Fig. S3A). Furthermore, individual samples are collected by by time points (d1, d3, d7, d14, d28, d42) post-chondrogenic induction due to prohibitive cost of the sequencing per sample. However, two batches of d28 samples were collected from independent experiments for scRNA-seq. Canonical correlation analysis (CCA) was used to align cells from the 2 batches16 (Fig. S3B). The cells in the same cluster from different batches exhibited high correlation in their gene expression (Spearman's rank coefficient rs > 0.87 for all clusters) (Fig. S3C). Furthermore, genes that were highly conserved in one particular cluster (using cluster 0 as an example) showed similar expression patterns in the clusters from distinct batches, suggesting that our differentiation is highly reproducible.<br><br>For biochemical analysis of chondrogenic pellets, power analysis is performed for sample size estimation first based on our previously published studies(Wu et al., Annals of the rheumatic diseases. 2015;74(11):2076-83), in which the effect size is using Cohen's criteria(Cohen J. Statistical power analysis for the behavioral sciences. 2nd. Hillsdale, NJ: erlbaum; 1988.). With an alpha = 0.05, power = 0.80, effect size = 0.8, the projected sample size for this study is approximately n = 26 each group. However, based on our previous (Wu et al., Arthritis & Rheumatology. 2017;69(9):1772-83.) and others' published articles (Wangler et al. Osteoarthritis and Cartilage. 2019; 27(7):1094-1105), n = 3-4 pellets per group is sufficient to detect statistical significance at the 95% confidence level.
Data exclusions	For quality control and to determine the heterogenous composition of cell populations, gene barcode matrices were input into the Seurat R package (version 2.4). Low-quality cells, defined as cells expressing less than 200 genes, more than 7,000 genes, and greater than 5% mitochondrial gene content, were removed. Note that these cutoff criteria need to be adjusted for individual studies, as gene expression levels can be cell type-dependent. Genes that were detected in less than three cells were also removed. Next, to reduce the variance introduced by "unwanted" sources, we regressed out variation in gene expression driven by cell cycle stages and mitochondrial gene expression with the vars.to.regress argument in the function ScaleData in Seurat. This exclusion criteria were ore-established based on the instruction provided on the Seurat website. All the scRNA-seq data analyses were performed after data filtering and cleaning (after i.e., quality control steps).
Replication	For single cell RNA sequencing (scRNA-seq), two batches of d28 samples were collected from independent experiments for scRNA-seq to evaluate the data reproducibility. Sequencing experiments were generally performed in biological duplicates. The high correlations between two batches of our d28 samples suggests that our experiments is highly reproducible. Furthermore, multiple independent cell lines were used for the current study to ensure the validity of our protocol and experiments. The chondrogenesis of all the independent cell lines will performed independently and the results are highly reproducible.
Randomization	The collected/harvested samples was randomized and allocated into experiments.
Blinding	The collected/harvested samples was randomized and allocated into experiments. The Researchers were blinded to group allocation. However, the bioinformatic analyses of the scRNA-seq samples were not randomized as it is required to know the time points of samples in order to construct differentiation trajectory.

Reporting for specific materials, systems and methods

We require information from authors about some types of materials, experimental systems and methods used in many studies. Here, indicate whether each material, system or method listed is relevant to your study. If you are not sure if a list item applies to your research, read the appropriate section before selecting a response.

Materials & experimental systems

n/a	Involved in the study	
[ ]	[x]	Antibodies
[ ]	[x]	Eukaryotic cell lines
[x]	[ ]	Palaeontology and archaeology
[ ]	[x]	Animals and other organisms
[x]	[ ]	Human research participants
[x]	[ ]	Clinical data
[x]	[ ]	Dual use research of concern

Methods

n/a	Involved in the study	
[x]	[ ]	ChIP-seq
[ ]	[x]	Flow cytometry
[x]	[ ]	MRI-based neuroimaging

Antibodies

Antibodies used	COL1A1 Abcam Cat#90395 (1:800)<br>COL2A1 Iowa Hybridoma Bank Cat#II-II6B3-s (1:10)<br>COL6A1 Fitzgerald Industries Cat#70F-CR009X (1:1000)<br>COL10A1 Sigma Cat#C7974 (1:200)<br>Goat Anti-Mouse Abcam Cat#97021 (1:500)<br>Goat Anti-Rabbit Abcam Cat#6720 (1:500)<br>Anti-Nestin antibody Abcam Cat#ab105389 (1:300)<br>Anti-Wnt2B Abcam Cat#ab178418 (1:350)<br>Anti-Wnt5B Abcam Cat#ab93134(1:500)<br>Anti-Wnt3A Abcam Cat#ab81614 (1:1000)<br>Anti-Wnt4 Abcam Cat#ab91226 (1:500)<br>Anti-Wnt7B Abcam Cat#b155313 (1:2000)<br>FITC-CD45 Biolegend Cat#304006 (5 µl per million cells in 100 µl staining volume)<br>PE/Cy7-CD146 Biolegend Cat#361008 (5 µl per million cells in 100 µl staining volume)<br>PE-CD166 Biolegend Cat#343904 (5 µl per million cells in 100 µl staining volume)<br>Tru Stain FC XTM Biolegend Cat#422302 (10 ul per million cells in 100 µl staining volume)<br>DAPI Biolegend Cat#422801 (3 µM used for flow cytometry)<br>Affinity purified horseradish peroxidase(HRP)-linked goat anti-rabbit IgG secondary antibody (1:3000, Cell Signaling, #7074),<br>Horse anti-mouse IgG secondary antibody (1:3000, Cell Signaling, #7076)<br>anti-GAPDH Proteintech Cat #60004-1-Ig (1:30000)
Validation	All the antibodies used were validated by the manufactures and the validating data was provided on their websites. Furthermore, we also used both positive and negative staining controls to further validate each antibody in our experiments.

Eukaryotic cell lines

Policy information about cell lines

Cell line source(s)	Three distinct human induced pluripotent stem cells (hiPSC) lines were used in the current study: STAN, ATCC, and BJFF. STAN line was purchased from WiCell (#STAN061i-164-1), ATCC line was acquired from ATCC (#ATCCACS-1019), and BJFF was obtained from the Genome Engineering and iPSC Core at Washington University in Saint Louis. All three lines were reprogrammed by Sendai virus from human foreskin fibroblasts.
Authentication	All cell lines used are evaluated to have pluripotency using terotomera formation and confirmed to be karyotypically normal.
Mycoplasma contamination	All cell lines used are mycoplasma free.
Commonly misidentified lines<br>(See ICLAC register)	No commonly misidentified cell lines were used in the study.

Animals and other organisms

Policy information about studies involving animals; ARRIVE guidelines recommended for reporting animal research

Laboratory animals	male NOD mice (NOD.Cg-Prkdcscid Il2rgtm1Wjl/SzJ, #005557, Jackson laboratory) at age of 18-20 weeks-old were used for human xenograft implantation in the in the dorsal region (subcutaneous) or in osteochondral defects in the knee joints of mice. Mice were housed under a 12hr light/12hr dark cycle with ambient temperature and humidity.
Wild animals	No wild animals were used in the study.
Field-collected samples	No field collected samples were used in the study.
Ethics oversight	All animal procedures were approved by Institutional Animal Care and Use Committee (IACUC) at Washington University in Saint Louis.

Note that full information on the approval of the study protocol must also be provided in the manuscript.

Flow Cytometry

Plots

Confirm that:

- [x] The axis labels state the marker and fluorochrome used (e.g. CD4-FITC).
- [x] The axis scales are clearly visible. Include numbers along axes only for bottom left plot of group (a 'group' is an analysis of identical markers).
- [x] All plots are contour plots with outliers or pseudocolor plots.
- [x] A numerical value for number of cells or percentage (with statistics) is provided.

Methodology

Sample preparation	Cells at the Cp stage with treatment of BMP4, a combination of BMP4 and WNT3A, or a combination of BMP4 and C59 were dissociated and resuspended in FACS Buffer (PBS-/- with 1% FBS and 1% penicillin/streptomycin/fungizone (P/S/F; Gibco) at approximately 40 x 106 cells/ml. The cells were treated with Human Tru Stain FC XTM (BioLegend, #422302) for 10 min at room temperature. Approximately 10,000 cells in 100 µl were used for each compensation. Cells were labeled with appropriate antibodies including their associated isotype control (FITC-CD45, #304006; PE/Cy7-CD146, #361008; PE-CD166, #343904, all from BioLegend). Cells were incubated for 30 minutes at 4ºC and washed with FACS buffer twice. Samples were resuspended in sorting medium consisting of DMEM/F12 with 2% FBS, 2% P/S/F, 2% HEPES (Gibco), and DAPI (BioLegend, #422801) at 4 x 106 cells/ml and filtered through a 40 µm cell strainer. Cells were stored on ice prior to sorting.
Instrument	Aria-II FACS machine (BD Biosciences)
Software	FlowJo software (version 10.5.3)
Cell population abundance	> 99% of parent cells are positive for the markers of interest in post-sort, indicating the validity of the sorting.
Gating strategy	Determination of FSC/SSC was relatively straightforward as all cells formed one dense and clear cluster in FSC/SSC plot.

- [x] Tick this box to confirm that a figure exemplifying the gating strategy is provided in the Supplementary Information.
