# Supplementary material for: Single cell transcriptomic analysis of human pluripotent stem cell chondrogenesis
Source: Nat Commun. 2021 Jan 13;12:362. doi: 10.1038/s41467-020-20598-y (PMC7806634; doi:10.1038/s41467-020-20598-y)
Supplement: Supplementary file 3 — Source Data [file 41467_2020_20598_MOESM3_ESM.pdf]

Source Data File

WNT2B

WNT2B antibody

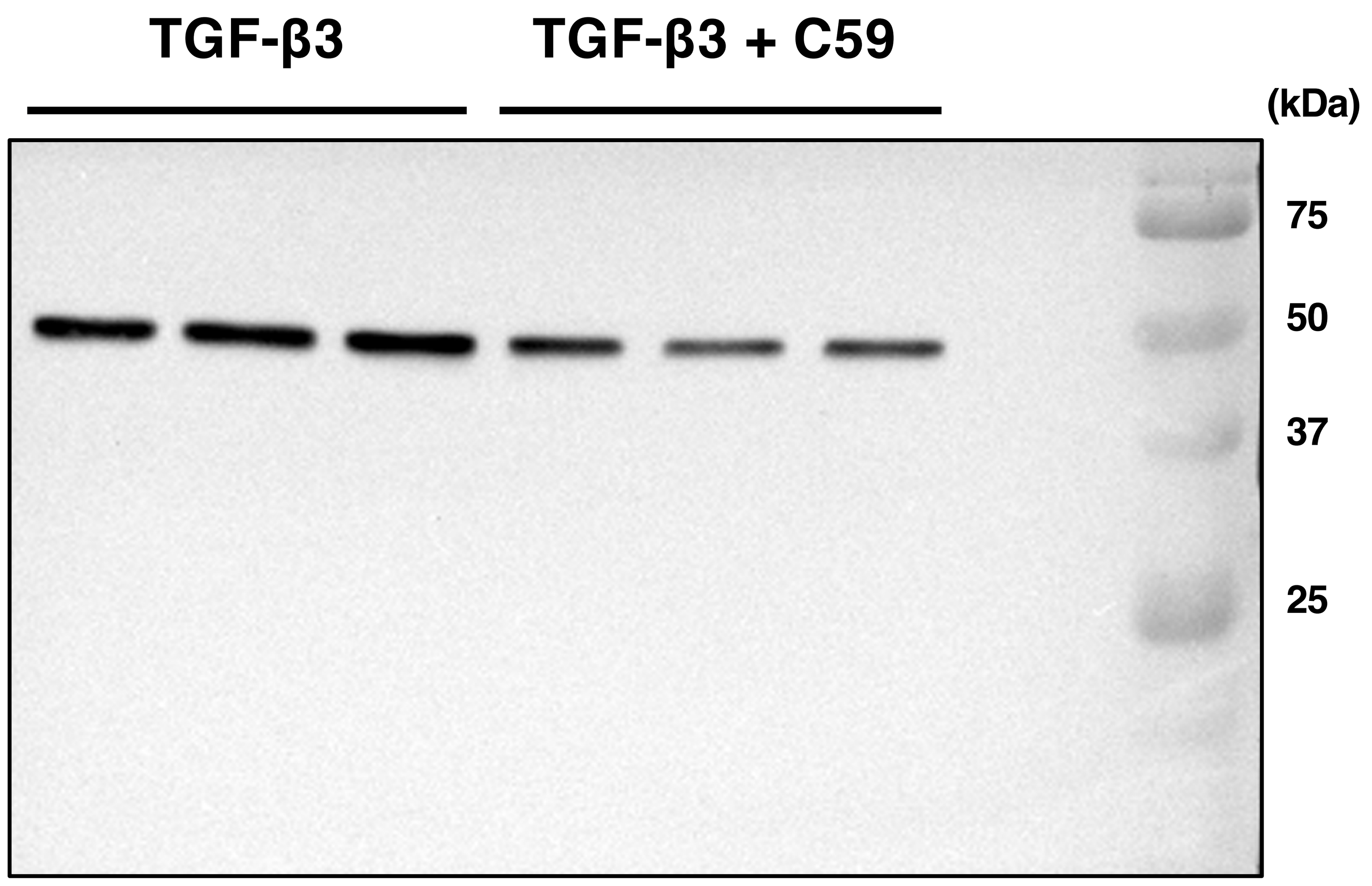

GAPDH antibody

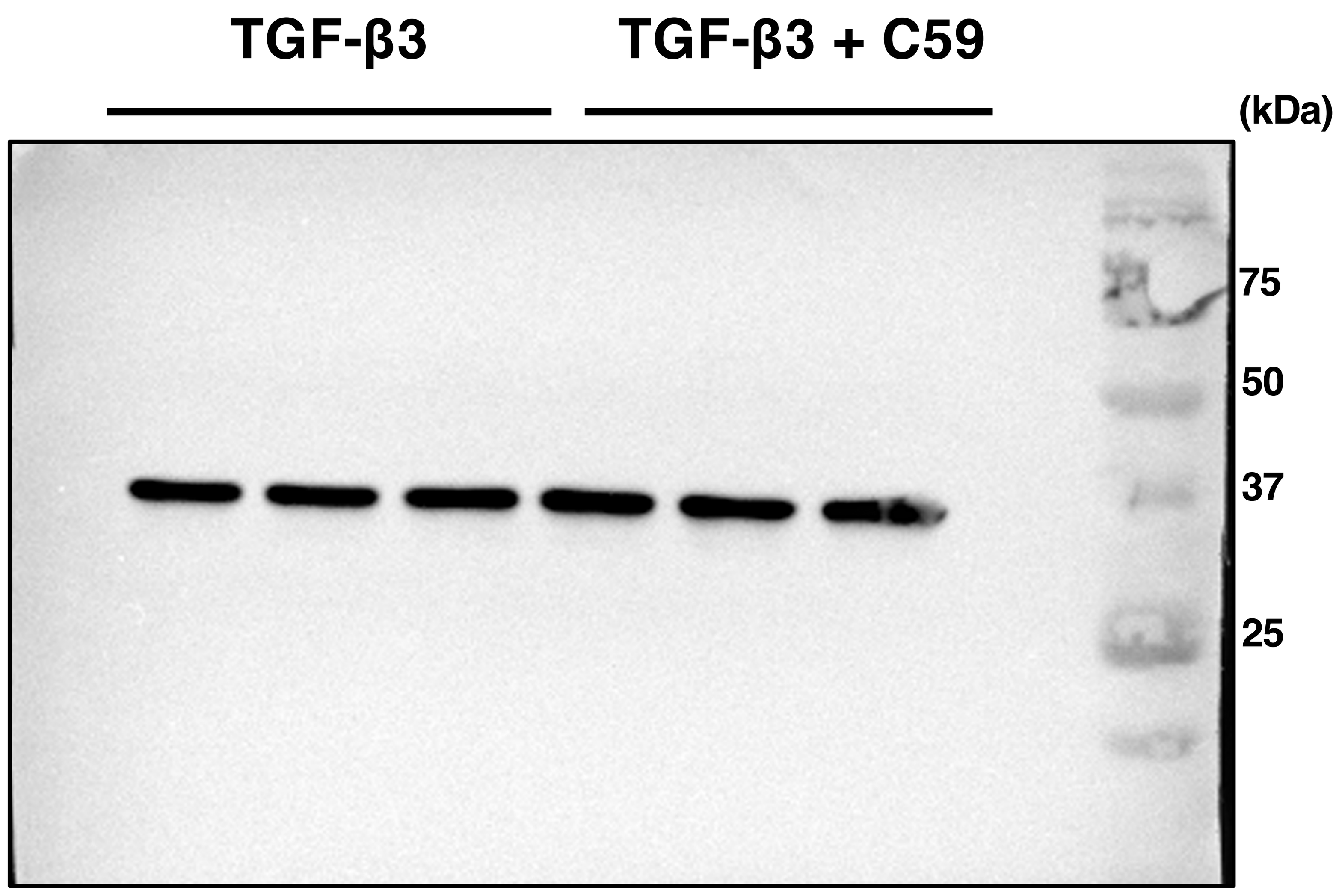

WNT3A

WNT3A antibody

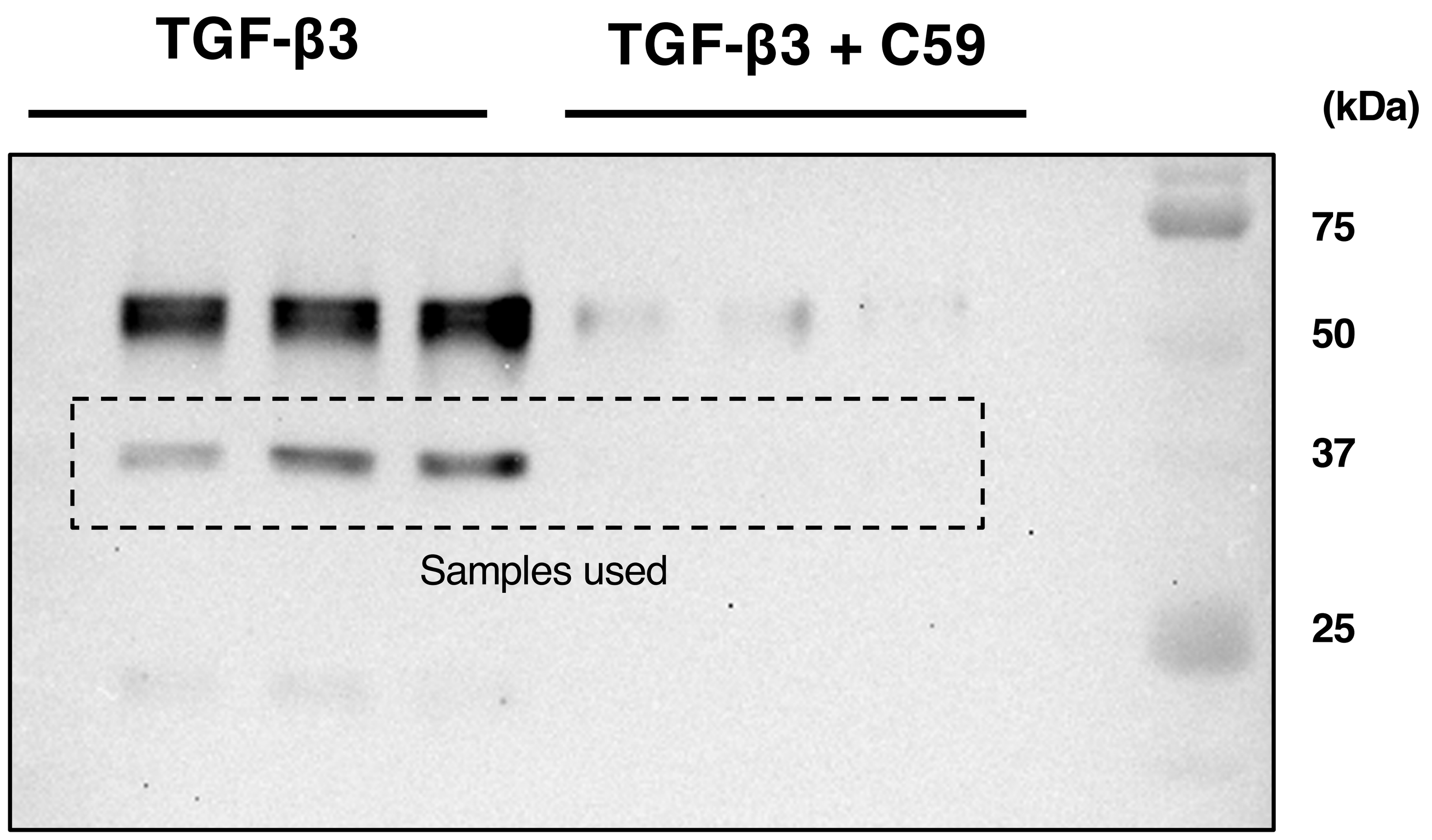

GAPDH antibody

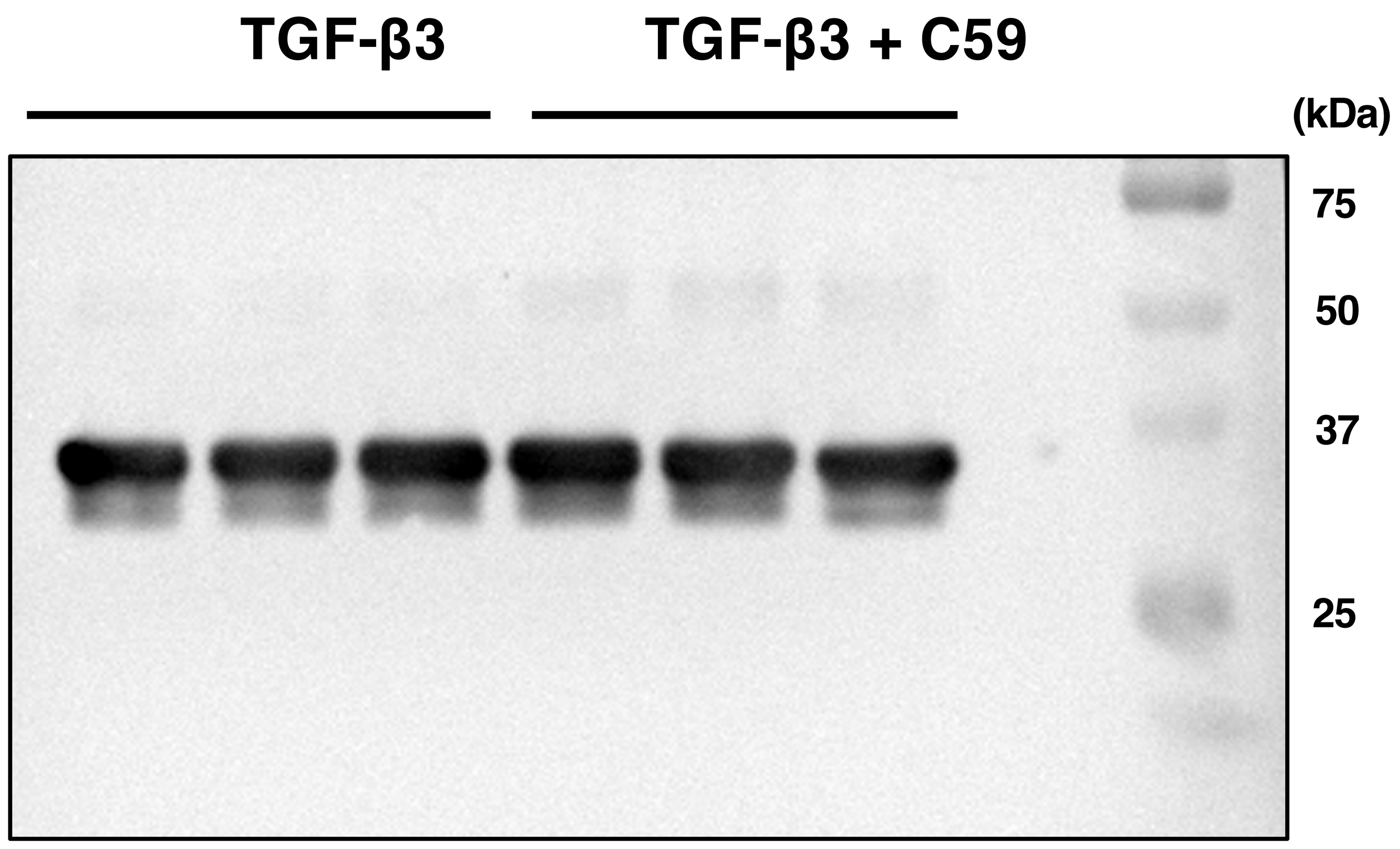

WNT4

WNT4 antibody

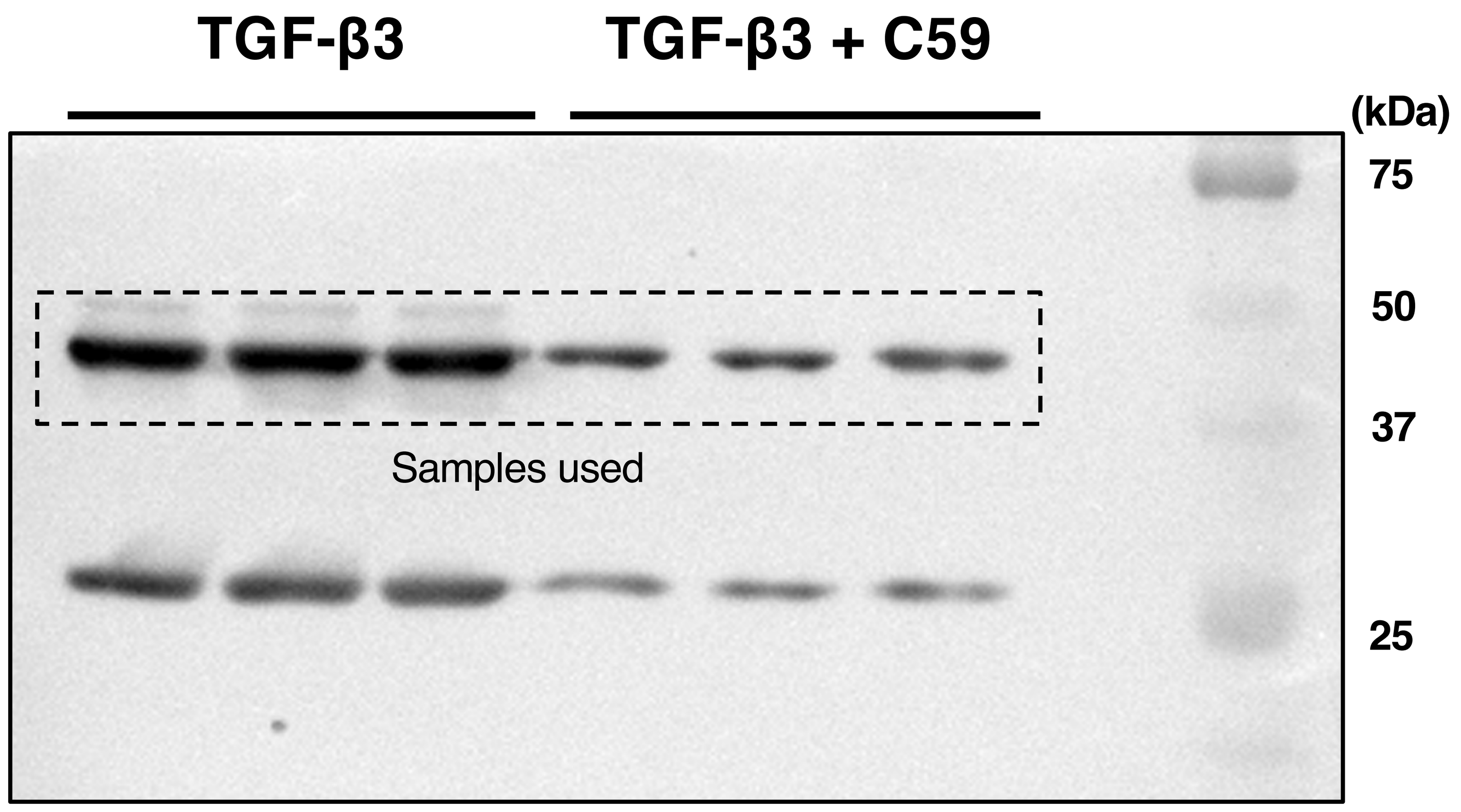

GAPDH antibody

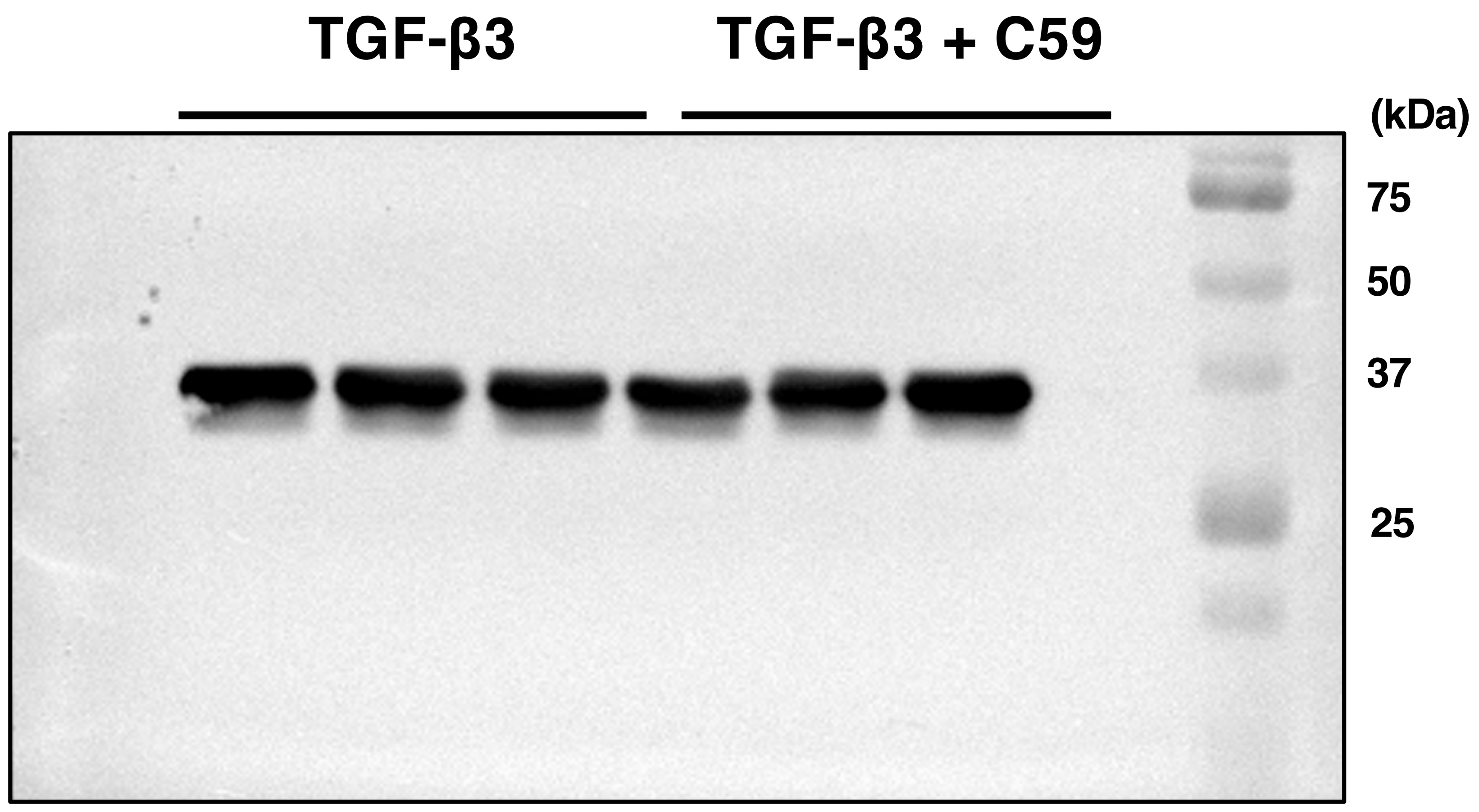

WNT5B

WNT5B antibody

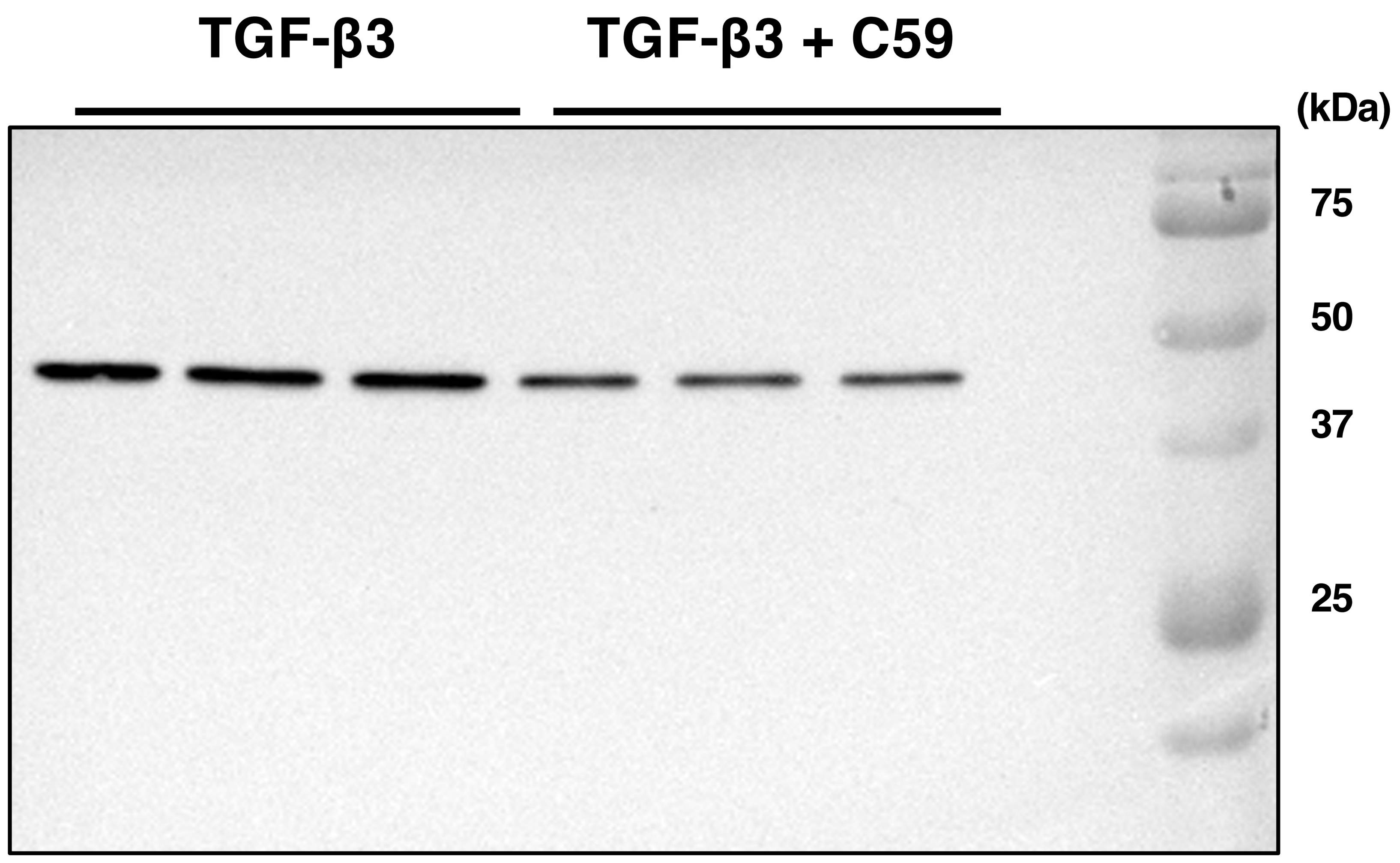

GAPDH antibody

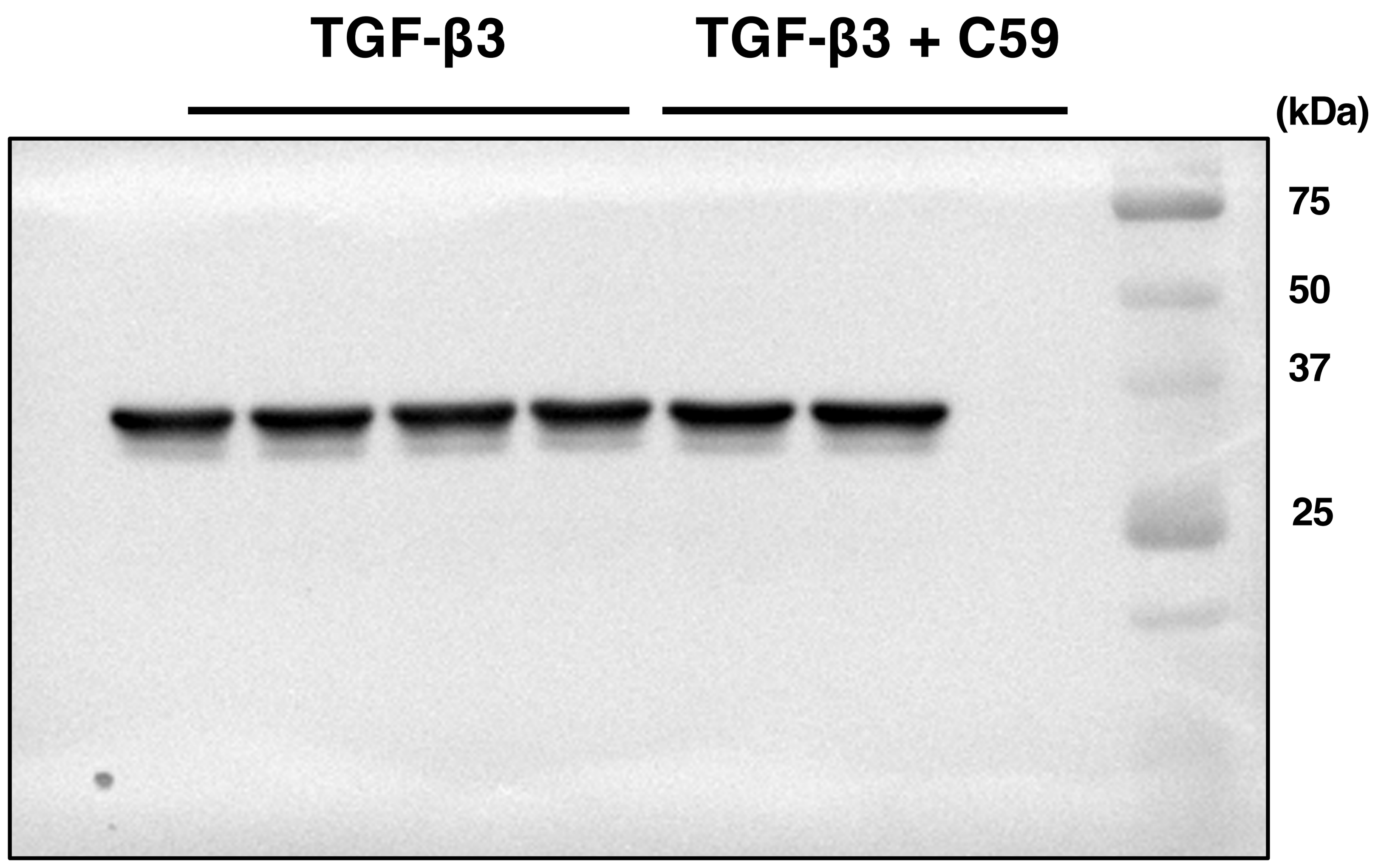

WNT7B

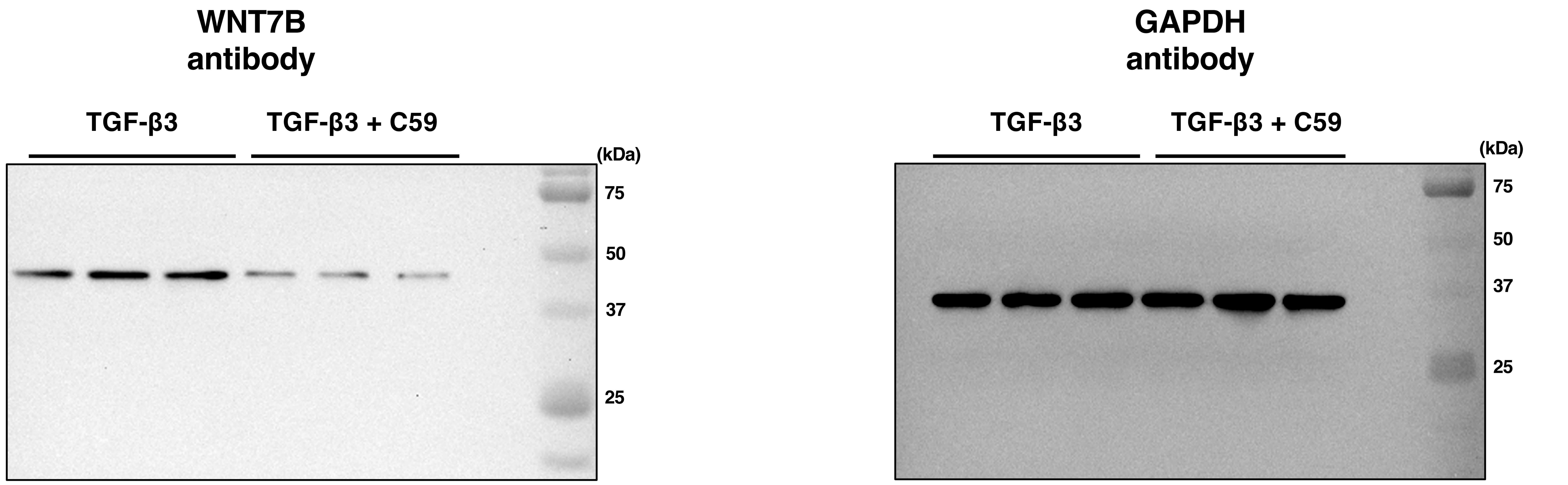
WNT7B
antibody
TGF-β3
TGF-β3 + C59
(kDa)
75
50
37
25
GAPDH
antibody
TGF-β3
TGF-β3 + C59
(kDa)
75
50
37
25
